# Mapping the causal landscape of polycystic ovary syndrome: A systematic Mendelian randomization evidence synthesis and the MR-PCOS atlas platform

**DOI:** 10.1097/MD.0000000000050366

**Published:** 2026-08-21

**Authors:** Sanwei Du, Dandan Dai, Wenhao Pan, Junlin Zhou, Kexin Wang

**Affiliations:** a Department of Gynecology and Obstetrics, Dongguan Hospital of Guangzhou University of Chinese Medicine, Dongguan, China; b Department of Internal Medicine, The Shenzhen Clinical College, Guangzhou University of Chinese Medicine, Shenzhen, China; c Department of Cardiology, Guangzhou University of Chinese Medicine, Guangzhou, China; d Department of Neurology, The First Clinical Medical College, Guangzhou University of Chinese Medicine, Guangzhou, China; e Department of Orthopedics, Clifford Hospital, Guangzhou University of Chinese Medicine, Guangzhou, China.

**Keywords:** causality, Mendelian randomization, polycystic ovary syndrome, risk factors, web application

## Abstract

Mendelian randomization (MR) studies have increasingly investigated potential predicted antecedents and consequences of polycystic ovary syndrome (PCOS), yet published evidence remains scattered across diverse exposures, outcomes, and analytical frameworks. This study aimed to systematically organize published PCOS-related MR evidence and develop an interactive platform to support evidence retrieval, comparison, and interpretation. We searched PubMed, Web of Science, Embase, and the Cochrane Library from inception to June 1, 2026. Original MR Studies were eligible if PCOS was either the exposure or the outcome, with extractable effect estimates. Each causal pathway was extracted separately for direction, definitions, effect estimates, primary MR method, Genome-Wide Association Study sources, ancestry, sample size, PCOS phenotype, sensitivity analyses, and sample overlap. Methodological appraisal followed Strengthening the Reporting of Observational Studies in Epidemiology-Mendelian Randomization. Evidence was summarized by direction, variable category, quality, and consistency. An interactive MR-PCOS Atlas was developed using R Shiny. Of 728 records identified, 306 original MR studies were included, yielding 1081 extractable causal pathways (763 with PCOS as outcome, 318 as exposure). Evidence concentrated in physiological traits and diseases/clinical phenotypes (312 and 299 pathways, respectively). Methodological quality was high in 13 studies, moderate in 279, and low in 14. Evidence most frequently addressed metabolic, endocrine, reproductive, and comorbidity pathways, while lifestyle, gut microbiota, immune phenotypes, and interventions remained limited. The MR-PCOS Atlas enables interactive browsing and comparison of published findings. This systematic synthesis provides a structured map of genetically supported PCOS associations and highlights areas of consistency, uncertainty, and methodological heterogeneity. Findings should be interpreted considering PCOS definitions, Genome-Wide Association Study sources, ancestry, and MR assumptions. The MR-PCOS Atlas serves as an evidence resource for retrieval and hypothesis generation, not for direct clinical recommendations.

## 1. Introduction

Polycystic ovary syndrome (PCOS) is a common endocrine-metabolic disorder affecting 5%–10% of women of reproductive age worldwide,^[1]^ characterized by oligo-ovulation/anovulation, hyperandrogenism, insulin resistance, and polycystic ovarian morphology.^[2]^ No curative treatment currently exists, and long-term management is required.^[3]^ Beyond genetic, intrauterine, and lifestyle factors, gut microbiota has also been implicated in its development,^[3]^ though the pathogenesis remains unclear.

PCOS is associated with increased risks of infertility, pregnancy complications, and miscarriage, as well as metabolic syndrome, depression, type 2 diabetes, anxiety, and cardiovascular disease (CVD).^[4]^ Patients also commonly present with metabolic abnormalities: elevated body mass index (BMI), hyperinsulinemia, hyperandrogenemia, and endothelial dysfunction,^[5,6]^ and have been linked to carcinogenic processes.^[7–9]^

Clarifying what precedes and what follows from PCOS is thus a prerequisite for improving its prevention and long-term management. However, observational epidemiological designs cannot, on their own, establish causality, since the associations they detect may simply reflect confounding. Mendelian randomization (MR) offers an alternative: made possible by large-scale genome-wide association studies (GWAS), this approach treats genetic variants as instrumental variables (IVs) from which causal links between exposures and outcomes can be inferred. Because these variants are assigned essentially at random at conception, MR is largely shielded from the confounding that limits conventional observational studies.^[10]^ Building on GWAS summary statistics, MR therefore provides a useful framework for probing possible causal links between the gut microbiome and PCOS, with the microbial exposures specified as IVs so that their causal effects can be estimated.^[11]^

However, findings for the same exposure often differ across populations as PCOS-related MR studies rapidly accumulate, complicating evidence synthesis. This study aimed to systematically organize and categorize published PCOS-related MR studies and develop a publicly accessible web application enabling rapid access to evidence on PCOS antecedents and consequences, thereby supporting evidence retrieval, comparison, and hypothesis generation for precision management.

## 2. Methods

This work is a systematic review and secondary analysis built entirely on previously published GWAS summary statistics; it did not involve the collection of new data from human participants or animals, nor any personally identifiable information. Ethical approval and informed consent were therefore not applicable to this study.

### 2.1. Definition of MR

This systematic review was not prospectively registered in PROSPERO or other registries, as it focuses on MR studies of genetic associations rather than clinical interventions or epidemiological observational studies that typically require prospective registration. Nonetheless, a detailed analysis plan, including the research question, eligibility criteria, search strategy, data extraction items, and planned evidence synthesis approach, was predefined before the initiation of data extraction. The analysis plan is available from the corresponding author upon reasonable request.

As an analytic strategy, MR uses genetic variants, most often single nucleotide polymorphisms (SNPs), as proxies for an exposure of interest, allowing the causal effect of that exposure on an outcome to be estimated. Because alleles are allotted essentially at random during meiosis under Mendel’s laws, this design approximates a naturally occurring randomized trial and is therefore comparatively resistant to confounding and reverse causation relative to conventional observational designs. Even so, MR is not free of limitations, since its estimates can still be distorted by weak instruments, horizontal pleiotropy, population stratification, selection bias, phenotype misclassification, and sample overlap.^[12–14]^ Depending on the source of the exposure and outcome data, MR studies are generally categorized as single-sample or two-sample designs. Two-sample MR, the more commonly used of the 2, typically draws on publicly available GWAS summary datasets: for example, UK Biobank, FinnGen, BioBank Japan, and IEU-OpenGWAS; such data are commonly retrieved and analyzed through resources such as MR-Base and the TwoSampleMR package.^[15–18]^ Valid genetic instruments must satisfy 3 core assumptions. The first is relevance: the selected SNPs need to show a robust association with the exposure, conventionally set at *P* < 5 × 10^−8^, although looser thresholds of 5 × 10^−7^ or 5 × 10^−6^ are sometimes adopted when instrument numbers are otherwise too limited.^[19]^ The second is independence, meaning the instruments must not be related to known confounders: a property that can be screened with tools such as LDtrait and probed further through sensitivity analyses.^[20]^ The third is exclusion restriction, requiring that the instruments influence the outcome solely through the exposure under study rather than through alternative biological routes, a condition typically evaluated against the existing literature together with supplementary analyses.^[21]^ When these assumptions are reasonably satisfied, MR can provide more credible causal inference in settings where randomized trials are infeasible or ethically challenging.

### 2.2. Identification of PCOS-related causal pathways

This study followed the Preferred Reporting Items for Systematic Reviews and Meta-Analyses 2020 reporting standard throughout its design and write-up. Records were retrieved from PubMed, Web of Science Core Collection, Embase, and the Cochrane Library, combining controlled-vocabulary and free-text terms built around 2 core concepts, “PCOS” and “MR.” Term lists were first broadened using MeSH, Emtree, and related synonyms, after which the search syntax was tailored to the rules of each individual database. Searches spanned from each database’s inception through June 1, 2026. The strategy used in PubMed was as follows: ((“PCOS”[Title/Abstract] odds ratio (OR) “Polycystic Ovarian Syndrome”(Title/Abstract] OR PCOS[Title/Abstract] OR “Ovary Syndrome, Polycystic”[Title/Abstract] OR “Syndrome, Polycystic Ovary”[Title/Abstract] OR “Ovarian Syndrome, Polycystic”[Title/Abstract] OR “Sclerocystic Ovarian Degeneration”[Title/Abstract] OR “Ovarian Degeneration, Sclerocystic”[Title/Abstract] OR “Sclerocystic Ovary Syndrome”[Title/Abstract] OR “Stein-Leventhal Syndrome”[Title/Abstract] OR “Stein Leventhal Syndrome”[Title/Abstract] OR “Syndrome, Stein-Leventhal”[Title/Abstract] OR “Sclerocystic Ovaries”[Title/Abstract] OR “Ovary, Sclerocystic”[Title/Abstract] OR “Sclerocystic Ovary”[Title/Abstract]) AND (“MR”[Title/Abstract] OR “MR”[Title/Abstract] OR “Mendelian Randomization Analysis”[Title/Abstract] OR “two-sample MR”[Title/Abstract] OR “two sample MR”[Title/Abstract] OR “bidirectional MR”[Title/Abstract] OR “multivariable MR”[Title/Abstract] OR “mediation MR”[Title/Abstract] OR “genetic IV”[Title/Abstract])).

All search results were imported into NoteExpress reference management software for deduplication. The complete search strategies for PubMed, Web of Science Core Collection, Embase, and the Cochrane Library are provided in Supplementary Table S1, Supplemental Digital Content 1. Study selection consisted of 2 stages: title-and-abstract screening and full-text assessment. Title-and-abstract screening was performed based on the title and abstract, and full-text assessment was performed based on the complete article. Data extraction and methodological quality appraisal were also independently conducted by 2 researchers using prespecified forms, with disagreements resolved by a third researcher. We screened for retracted or corrected publications using the Retraction Watch database (http://retractionwatch.com/) and cross-checked the included studies against the database. When multiple reports from the same underlying analysis or the same GWAS dataset were identified, we retained the most complete or most recent version and documented the excluded duplicates in the supplementary materials. The inclusion criteria were as follows: original MR studies; PCOS located at one end of at least one causal pathway, as either the exposure or the outcome; and extractable PCOS-related effect estimates with 95% confidence intervals (CIs). The exclusion criteria were non-original MR studies, such as reviews, systematic reviews, meta-analyses, protocols, editorials, or commentaries; studies that did not use MR methods; studies in which PCOS was not located at either end of an MR causal pathway; and studies from which valid effect estimates could not be extracted. We restricted the search to studies published in English. No other language restrictions were applied. In addition to the database searches, we manually screened the reference lists of all included studies and relevant systematic reviews to identify additional eligible records that may not have been captured by the primary electronic searches.

For each included study, we extracted the first author, publication year, journal, DOI/PubMed identifier, MR type, the role of PCOS in the causal pathway, exposure and outcome names, effect estimates, 95% CIs, *P* values, primary MR method, GWAS data sources for the exposure and outcome, ancestry, sample size, PCOS definition or diagnostic criteria, sensitivity analysis methods, and information on sample overlap. If a study reported multiple PCOS-related causal pathways, each pathway was extracted separately. If only pooled results were reported without pathway-specific effect estimates, these results were not counted in the total number of extractable causal pathways.

To address the influence of differences in MR study quality on evidence interpretation, we further developed a methodological quality appraisal framework based on Strengthening the Reporting of Observational Studies in Epidemiology-Mendelian Randomization (STROBE-MR)-related principles. This framework focused on the instrument selection threshold, linkage-disequilibrium clumping parameters, weak-instrument assessment, and evaluation of horizontal pleiotropy, heterogeneity testing, robustness or directionality analyses, multivariable or mediation MR adjustment, multiple-testing correction, and overall completeness of methodological reporting. Each study was preliminarily rated as high, moderate, low, or uncertain quality.

For evidence synthesis, we summarized the evidence in a structured manner according to the direction of the causal pathway, the category of the non-PCOS variable, and evidence consistency. For the same causal relationship repeatedly evaluated by multiple independent MR studies, we compared the direction of effect, statistical significance, sample source, ancestry, and methodological quality, and classified the evidence as consistent evidence, suggestive evidence, conflicting evidence, null evidence, or insufficient evidence.

We restricted the main analysis to peer-reviewed full-text original MR studies because reliable extraction of MR design, GWAS data sources, effect estimates, CIs, and sensitivity analyses was required. Grey literature, conference abstracts, and preprints were not included in the main synthesis. This restriction was applied to improve the methodological verifiability and reproducibility of the extracted evidence, although it may have led to the omission of some very recent or unpublished MR findings.

The extracted information focused on causal pathways. According to the role of PCOS, causal pathways were classified as “antecedent → PCOS” or “PCOS → consequence.” Non-PCOS variables were further categorized into 9 groups: metabolites, physiological traits, interventions, diseases or clinical phenotypes, proteins or genes, microbiota, nutrition-related metabolites, immune-cell phenotypes, and lifestyle factors. We also extracted the PCOS definitions or phenotype information used in each included MR study, including diagnostic criteria, self-reported PCOS, definitions based on International Classification of Diseases codes or electronic health records, FinnGen-defined PCOS, UK Biobank-defined PCOS, GWAS consortium-defined PCOS, and overall PCOS when available. We assessed the feasibility of phenotype-specific evidence synthesis. Because most original MR studies used summary-level GWAS data for overall PCOS or database-defined PCOS and did not provide phenotype-specific estimates, formal subgroup evidence synthesis by PCOS phenotype was not performed.

For statistical reporting, we preferentially extracted the effect estimate from the primary MR model specified by the original study. If the primary model was not explicitly defined, the inverse-variance weighted (IVW) estimate was used as the main estimate when available. Effect estimates were reported on their original scales, including ORs, beta coefficients, hazard ratios, or risk ratios. Beta coefficients were not converted to ORs unless the original article explicitly stated that they were on the log-odds scale. Effect estimates and their 95% CIs were standardized to 2 decimal places, and *P* values were standardized for tabular presentation. No formal multiple-testing correction (e.g., Bonferroni or false discovery rate adjustment) was applied to the overall evidence synthesis, as this review is exploratory in nature and intended to systematically map the available evidence landscape. However, we reported whether the original studies applied multiple-testing correction and noted the correction status for each included study in the supplementary tables to facilitate interpretation of the credibility of individual findings.

When multiple MR studies used the same or overlapping GWAS data sources (e.g., multiple analyses based on UK Biobank, FinnGen, or the same PCOS GWAS consortium), we retained the most recent or most comprehensive analysis for the same exposure-outcome pair and flagged overlapping evidence in the extracted data table. Duplicate reports derived from the identical underlying analytic sample were excluded from the pathway count, and the reasons for exclusion were documented in the supplementary materials.

### 2.3. Methodological quality appraisal

Building on the STROBE-MR principles introduced above, our appraisal tool scored each study across ten domains: the *P*-value threshold used for instrument selection (typically < 5 × 10^−8^, with any relaxation noted), the linkage-disequilibrium clumping parameters applied, the number of SNPs serving as instruments, whether instrument strength was checked (e.g., via reported *F*-statistics), whether horizontal pleiotropy was evaluated (e.g., MR-Egger intercept, MR-PRESSO), whether heterogeneity was tested (e.g., Cochran’s *Q*), whether robustness or directionality was probed (e.g., leave-one-out analysis, Steiger filtering, bidirectional MR), whether multivariable or mediation MR adjustment was carried out, whether correction for multiple-testing was applied, and how clearly the PCOS phenotype was defined, alongside the overall completeness of methods reporting. Each eligible study was scored independently by 2 reviewers using a shared, standardized extraction form; disagreements were first discussed between the 2 reviewers and, where consensus could not be reached, referred to a third reviewer. A rating of high quality was given when nearly all core items were adequately reported with no major concerns; moderate quality when the core MR design and some sensitivity checks were present but several key items were missing; low quality when central MR assumptions, sensitivity analyses, or outcome reporting were clearly inadequate; and uncertain quality when the source article simply did not provide enough information to judge. These ratings were used only to help interpret how credible and consistent the evidence was across causal pathways, not to exclude studies from the synthesis.

For evidence synthesis, we performed a narrative synthesis following the principles of the Synthesis Without Meta-analysis (SWiM) reporting guideline. Given the heterogeneity in exposure definitions, outcome measures, PCOS phenotypes, and MR analytical methods across studies, a meta-analytic approach was not feasible. Instead, we structured the synthesis by grouping evidence according to the role of PCOS in each causal pathway (PCOS as outcome versus PCOS as exposure) and by the category of the non-PCOS variable (metabolites, physiological traits, interventions, diseases or clinical phenotypes, proteins or genes, microbiota, nutrition-related metabolites, immune-cell phenotypes, and lifestyle factors). For each causal relationship, we compared the direction of effect, statistical significance, sample source, ancestry, and methodological quality across studies, and classified the evidence as consistent evidence, suggestive evidence, conflicting evidence, null evidence, or insufficient evidence.

### 2.4. Development of the MR-PCOS Atlas platform

Based on the Shiny package,^[22]^ we developed the MR-PCOS Atlas platform. The platform includes 2 modules. The first module was designed to help users rapidly identify PCOS-related MR studies under specified conditions, such as MR studies published in 2026. The second module was designed to facilitate comparison of MR findings for different indicators within the same category in relation to PCOS, or for PCOS in relation to different indicators within the same category. The Shiny application was deployed on a server to facilitate user access.

## 3. Results

### 3.1. Identification of PCOS-related causal pathways

**Figure 1.**
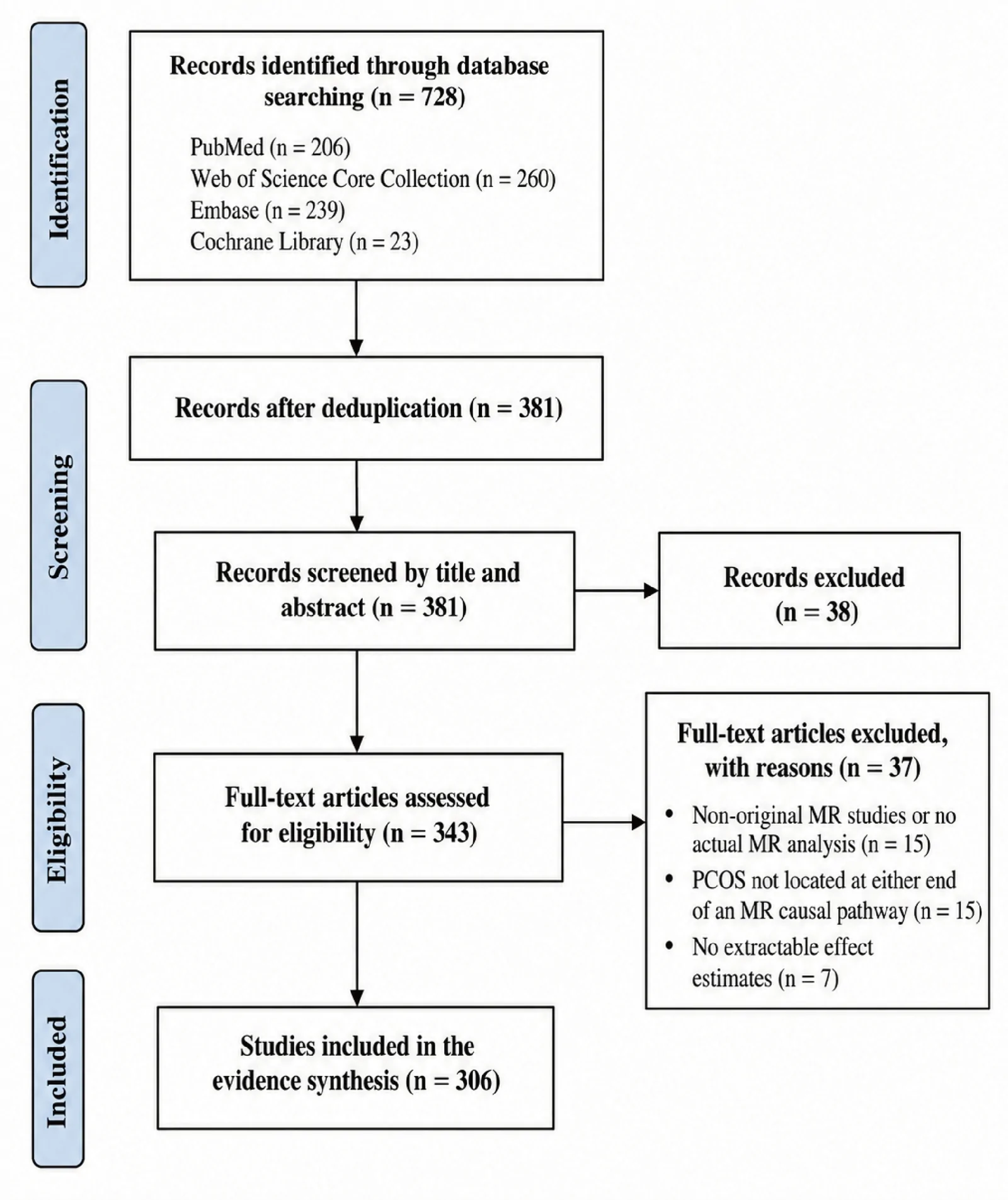
PRISMA flow diagram of study selection. MR = Mendelian randomization, PCOS = polycystic ovary syndrome, PRISMA = Preferred Reporting Items for Systematic Reviews and Meta-Analyses.

**Figure 2.**
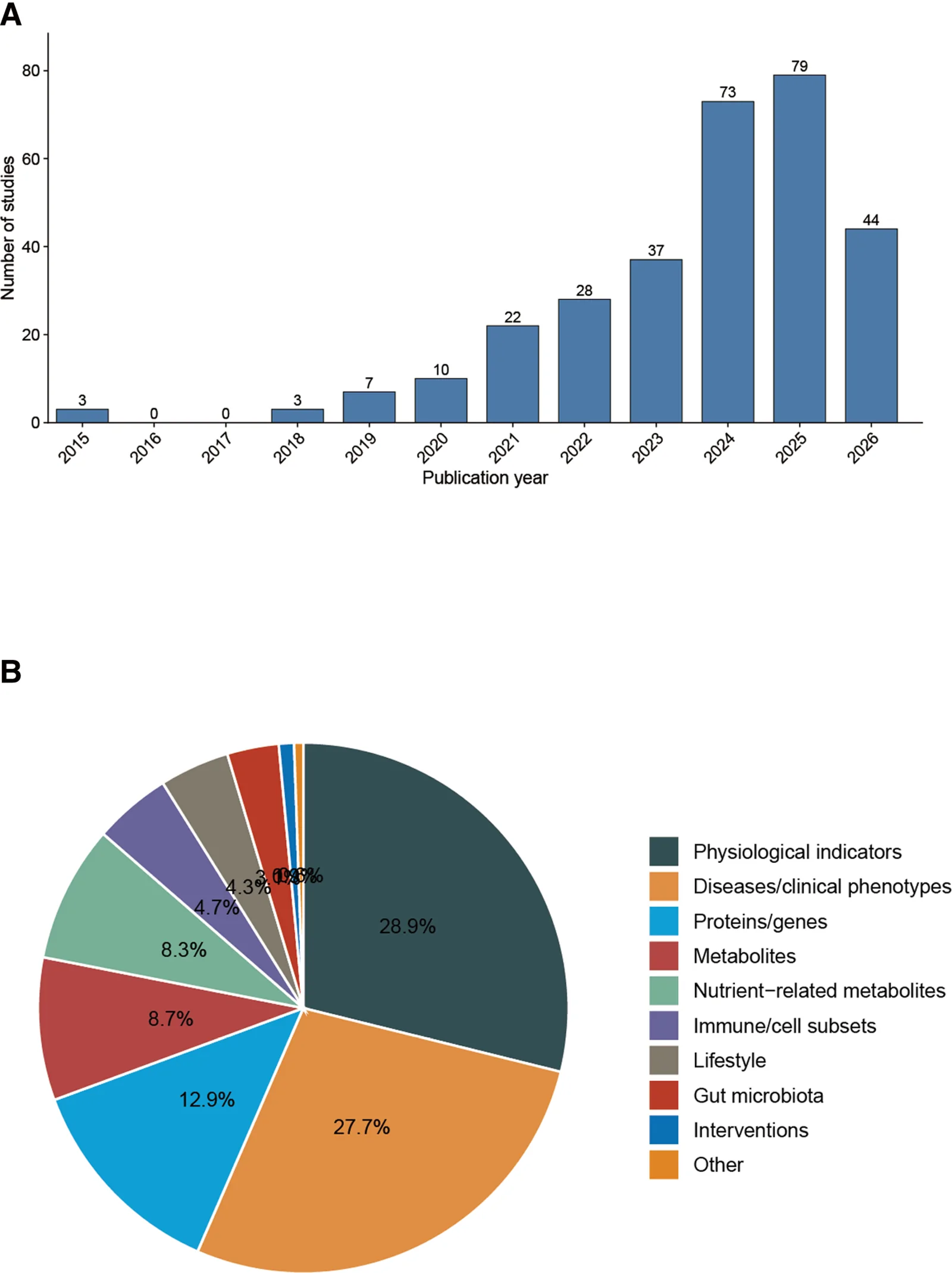
General characteristics of the included studies. (A) Annual publication volume of Mendelian randomization studies on PCOS from 2019 to 2025. (B) Causal categories of MR analyses on PCOS, classified by exposure-outcome domains. PCOS = polycystic ovary syndrome, MR = Mendelian randomization.

Based on the prespecified search strategy, we identified 728 records from PubMed, Web of Science Core Collection, Embase, and the Cochrane Library, including 206 from PubMed, 260 from Web of Science, 239 from Embase, and 23 from the Cochrane Library. After all records were imported into NoteExpress and duplicates were removed, 381 records entered title-and-abstract screening. At this stage, 38 records that clearly did not meet the inclusion criteria were excluded, leaving 343 articles for full-text assessment. After full-text assessment, 37 articles were further excluded: 15 were non-original MR studies or did not actually perform MR analyses, 15 were MR studies in which PCOS was not located at either end of the causal pathway, and 7 did not provide extractable valid effect estimates. Finally, 306 original PCOS-related MR studies were included. The articles excluded at the full-text stage and the specific reasons for exclusion are shown in Supplementary Table S2, Supplemental Digital Content 2, and the study selection process is shown in Figure 1. The annual number of included studies increased rapidly and peaked in 2025, with 79 studies (Fig. 2A). From the 306 included studies, we extracted 1081 usable PCOS-related MR causal pathways. PCOS was evaluated as the outcome in 763 pathways and as the exposure in 318 pathways. All extractable PCOS-related MR causal pathways and their effect estimates are provided in Supplementary Table S3, Supplemental Digital Content 3. According to the category of the non-PCOS variable, the current evidence was concentrated mainly in physiological traits and diseases or clinical phenotypes, including 312 and 299 causal pathways, respectively (Fig. 2B). These findings suggest that the current PCOS-MR evidence primarily addresses metabolic, reproductive endocrine, and comorbidity-related pathways, whereas evidence related to lifestyle factors, gut microbiota, immune-cell phenotypes, and interventions remains comparatively limited.

### 3.2. Methodological quality of the included MR studies

Based on the STROBE-MR-informed methodological appraisal framework, among the 306 included studies, 13 were rated as high quality, 279 as moderate quality, and 14 as low quality; no study was rated as uncertain quality. Overall, most studies clearly reported the primary MR method and performed at least 1 sensitivity analysis. However, reporting of sample overlap, multiple-testing correction, and PCOS phenotype definitions was inconsistent. These methodological features were used to interpret the consistency and credibility of evidence across different causal pathways, rather than as criteria for study exclusion. Detailed study-level methodological quality appraisal is provided in Supplementary Table S4, Supplemental Digital Content 4.

### 3.3. MR analyses of proteins/genes and PCOS

#### 3.3.1. Innate immune receptors and inflammasome-related genes

He et al reported that genetically increased levels of genes such as CD93, CYBB, NLRP1, and NOD2 were associated with a higher risk of PCOS.^[23]^ CYBB encodes a NADPH oxidase subunit and is involved in reactive oxygen species generation, whereas NLRP1 and NOD2 may contribute to inflammasome assembly. Excessive activation may promote local low-grade ovarian inflammation, follicular cell apoptosis, and subsequent insulin resistance. In contrast, He et al also reported a protective association for PTER.^[23]^ Its potential roles in suppressing inflammation and improving cell survival may help explain the opposite direction of association compared with the above risk genes.

#### 3.3.2. Circulating cytokines and their receptors

Through an integrated analysis of protein GWAS data, Li et al suggested that genetically increased interleukin (IL)6R was associated with a lower risk of PCOS, indicating that partial antagonism of classical IL-6 signaling may reduce the burden of chronic inflammation.^[24]^ Chen et al reported positive associations of genetically increased IL-2 and VEGF levels with PCOS, whereas IL-7, IL15RA, and CXCL11 showed negative associations.^[25]^ Jiang et al further reported that increased CCL4 and decreased CXCL11 were associated with higher disease risk.^[26]^ Taken together, these findings indicate that CXCL11, a chemokine family member, showed protective associations in 2 studies, whereas CCL4 appeared to be a risk-associated factor, suggesting functional heterogeneity among chemokine subtypes in local ovarian immune-cell recruitment. Tian et al also observed a modest positive association between OSM and PCOS.^[27]^ This cytokine may increase STAT3 signaling activity and potentially amplify insulin resistance and ovarian fibrosis. Thus, heterogeneity within cytokine profiles reflects the importance of fine regulation of inflammatory pathways: although these molecules are all related to inflammation, the genetic effect directions of different receptor-ligand axes are not consistent.

#### 3.3.3. Metabolism- and oxidative stress-related proteins

Zhang et al reported that genetically upregulated CDCP1, GLRX2, and KIRREL2 were associated with an increased risk of PCOS.^[28]^ GLRX2 is involved in glutathione and iron-sulphur cluster homeostasis, and its overexpression may contribute to oxidative phosphorylation dysfunction, whereas abnormal expression of KIRREL2 in pancreatic β-cell secretory pathways may impair insulin release. In an MR analysis of modifiable risk factors, Zhao et al noted that lipid metabolic disturbances were closely related to multiple protein factors, providing a broader metabolic context for these findings.^[29]^ Using epithelial-mesenchymal transition data, Liu et al suggested that key metabolic-structural genes may regulate ovarian stromal stiffness and inflammatory susceptibility,^[30]^ indicating potential coupling between metabolism and tissue remodeling.

#### 3.3.4. Hormone-binding and transport-related proteins

Guo et al reported that genetically higher sex hormone-binding globulin (SHBG) levels were associated with a significantly lower risk of PCOS.^[31]^ As a major carrier of sex steroids, increased SHBG may reduce free androgen levels and attenuate the hyperandrogenic phenotype of polycystic ovaries. In a plasma protein cohort, Li et al also identified several SHBG-regulating candidate proteins with indirect protective associations with PCOS.^[32]^ These findings suggest that hormone carrier proteins may be more than diagnostic biochemical markers and could represent potential therapeutic targets, although this requires further experimental and clinical validation. Increasing SHBG or modulating its upstream regulatory network may indirectly help correct hyperandrogenism and metabolic imbalance.

#### 3.3.5. Insulin signaling pathway genes

Ioannidis et al reported that the IRS1 Arg972 allele was associated with PCOS risk through higher fasting insulin levels.^[33]^ Although the INSR His1058C/T polymorphism did not reach statistical significance in that study, the overall pattern suggested that genetic alterations in insulin signaling and insulin resistance may represent important nodes in PCOS-related pathways. Compared with innate immune genes, these loci are more closely related to energy metabolism than to inflammation, but they may converge through the shared pathway of insulin resistance.

#### 3.3.6. Hemoglobin and hypoxia response

Using two-sample MR combined with multi-omics analyses, Wang et al reported a positive association between higher hemoglobin levels and PCOS, with significant enrichment of the HIF-1 signaling pathway.^[34]^ Higher hemoglobin levels may increase oxygen transport capacity, but may also be linked to compensatory micro-hypoxic responses in organs, activating HIF-1α-mediated glycolytic shifts and echoing the mitochondrial functional imbalance suggested by GLRX2. This finding broadens the scope of PCOS research and suggests that systemic oxygen sensing may also be involved in PCOS-related pathophysiology.

#### 3.3.7. Ribosomal and cell growth-regulatory genes

Through summary-data-based Mendelian randomization analysis, Sun et al reported pleiotropic associations between ribosomal protein genes, such as RPS26, and PCOS.^[35]^ Ribosomal proteins are involved in protein synthesis and p53-dependent cell-cycle checkpoints, and abnormal expression may contribute to arrested follicular growth. Compared with the inflammatory and metabolic genes described above, ribosomal proteins may act more strongly at the level of cell proliferation, providing another mechanistic dimension for growth regulation within the ovarian microenvironment.

Integrating the immune, metabolic, hormonal, and hypoxia-related pathways suggests a possible positive feedback loop linking inflammation, insulin resistance, and androgen excess. Upregulation of inflammasome-related genes and risk-associated chemokines may impair insulin signaling; insulin resistance may further enhance ovarian steroidogenesis; and low SHBG together with hyperandrogenism may amplify systemic inflammation. In parallel, oxidative stress and hypoxia responses driven by GLRX2- and hemoglobin-related genes may promote reactive oxygen species accumulation at the cellular level and further activate the NLRP1 inflammasome, forming an interlinked cycle of metabolic and immune imbalance. Protective genes, such as PTER, IL6R, CXCL11, and SHBG, may interrupt this loop by suppressing inflammation or improving hormonal balance, thereby showing associations in the opposite direction.

### 3.4. MR analyses of diseases/clinical phenotypes and PCOS

#### 3.4.1. Immune-mediated diseases

Across 2 published lines of evidence, a positive association between rheumatoid arthritis (RA) genetic liability and PCOS has been repeatedly reported. Using the IVW method, Wang et al observed that each standard deviation (SD) increase in genetic risk for RA was associated with an approximately 7% higher risk of PCOS (OR, 1.069).^[36]^ Xing et al obtained a similar estimate using another independent dataset (OR, 1.08).^[37]^ Neither study detected substantial heterogeneity or horizontal pleiotropy, suggesting that RA-related systemic inflammation or autoantibodies may interfere with follicular development. In contrast, other common autoimmune diseases showed null or weak associations. Chen et al performed a subtype-specific analysis of 9 type 1 diabetes subtypes but did not identify a significant genetically predicted association with PCOS.^[38]^ The authors proposed that, despite null statistical findings, ovarian autoimmune inflammation related to type 1 diabetes remains biologically plausible and requires confirmation in larger samples. Similarly, the ORs for systemic lupus erythematosus and polymyositis were close to 1 in 2 studies, with CIs crossing the null, supporting a limited influence on PCOS risk.^[36]^

#### 3.4.2. Mood disorders

Ling et al reported that genetic liability to depression or dysthymia was associated with a 43% higher risk of PCOS (OR, 1.43), with reverse causation not supported.^[39]^ This finding links mood regulation with reproductive endocrinology and suggests that the hypothalamic-pituitary-ovarian axis may be influenced by psycho-inflammatory pathways. In contrast, Jiang et al found that the weak positive association between PCOS and depression tended to disappear after adjustment for BMI in multivariable MR.^[40]^ The differences between these studies suggest that obesity or insulin resistance may partly mediate psycho-reproductive interactions, although the overall evidence still points to a positive association with liability to depression. Compared with RA, the OR for the depression phenotype was larger, suggesting that psychological-neuroendocrine mechanisms may have a more pronounced contribution to PCOS risk than immune inflammation alone.

Ling et al assessed the effects of female reproductive disorders on depression or dysthymic disorder and did not identify a PCOS-depression causal pathway.^[39]^ In contrast, Jiang et al., using analyses in the same direction, suggested that obesity may mediate a weak positive effect of PCOS on depression.^[40]^ The main difference between the 2 studies lies in mediator adjustment: Ling et al directly tested the PCOS-depression association, whereas Jiang et al included BMI in multivariable MR. If obesity is a necessary mediator, a simple model may yield null findings, whereas accounting for BMI may reveal an effect, suggesting that omission of the mediation pathway may partly explain the inconsistency.

#### 3.4.3. Thyroid dysfunction

Zhao et al systematically evaluated the effects of several thyroid-related indicators, including free thyroxine (FT4), thyroid-stimulating hormone (TSH), hyperthyroidism, and hypothyroidism, on PCOS. All IVW-derived ORs ranged from 0.88 to 1.20, with *P* values > .05, indicating no overall significant genetically predicted relationship.^[41]^ Although the authors observed in the reverse analysis that PCOS tended to be associated with lower TSH levels, thyroid function as an exposure did not increase susceptibility to PCOS. Another bidirectional MR study in the same field also did not report positive findings.^[42]^ Compared with RA and depression, the direction of effect for thyroid phenotypes remains unclear, and current evidence suggests a limited direct contribution to PCOS.

Zhao et al reported no unidirectional genetically predicted association between FT4, TSH levels, and PCOS, and also found no evidence that PCOS causally affects thyroid function in the reverse direction.^[41]^ However, Zhang et al reported a positive causal association between subclinical hypothyroidism and PCOS, and suggested that genetic liability to PCOS may modestly increase the risk of thyroid disease.^[42]^ The inconsistency in direction between the 2 studies may be attributable to several factors. First, population differences may have contributed: the former included only European populations, whereas the latter integrated some mixed-population GWAS data in addition to European data, and differences in genetic structure may have affected IV effect estimates. Second, exposure definitions differed: Zhao et al used 4 exposures, including continuous FT4/TSH levels and diagnosed hyperthyroidism and hypothyroidism, whereas Zhang et al combined analyses of hyperthyroidism/hypothyroidism with variation in TSH and FT4 within the normal reference range, increasing exposure heterogeneity.

#### 3.4.4. Infectious and other disease phenotypes

Using large consortium datasets, Si et al reported that 3 coronavirus disease 2019 phenotypes: susceptibility, hospitalization, and severe disease were not protective or risk factors for PCOS.^[43]^ Given the lack of a direct biological overlap between pulmonary infection events and ovarian function, this conclusion is consistent with expectations. Beyond this, there is currently insufficient MR evidence to support a significant causal effect of other infectious diseases on PCOS.

### 3.5. MR analyses of physiological traits and PCOS

#### 3.5.1. Endocrine traits

A two-sample MR study based on GWAS data from European populations showed that each SD increase in genetically predicted serum testosterone was associated with an approximately 60% higher risk of PCOS (IVW: OR, 1.606; 95% CI, 1.088–2.372; *P* = .019). Weighted median and MR-Egger estimates were directionally consistent, supporting the robustness of the association. The investigators further stratified analyses by PCOS subtype and found minimal differences in effect estimates under the Rotterdam, NIH, and self-reported diagnostic definitions, suggesting that the association between androgens and PCOS may not depend strongly on diagnostic criteria. Previous animal and clinical studies have long debated whether elevated testosterone is a cause or consequence, whereas MR findings tend to support the former interpretation. Katherine S. Ruth et al suggested that lifelong modestly higher testosterone exposure may contribute to ovarian dysfunction rather than merely representing a secondary phenomenon of ovarian pathology.^[40]^

SHBG is a key carrier protein determining free testosterone levels. Several MR studies have examined the directional relationship between SHBG and PCOS. Multi-omics studies have further provided evidence for causal pathways linking SHBG with insulin-glucose metabolic proteins. Using MR-integrated proteomic analysis in visceral adipose tissue, Yurong Zhang et al found that lower SHBG co-occurred with upregulated mitochondrial oxidative phosphorylation, suggesting that energy metabolic pathways may mediate its protective association.^[44]^

Anti-Müllerian hormone (AMH) is regarded as a serum marker of follicular reserve. Using genetic instruments constructed from 7 loci, a two-sample MR study did not observe a significant causal effect of AMH on PCOS. Results from multiple robustness methods were consistent, with no evidence of heterogeneity or horizontal pleiotropy.^[45]^ This finding suggests that elevated AMH in clinical settings may be more likely to reflect the accumulation of small antral follicles in the ovary rather than a causal driver of disease.

#### 3.5.2. Metabolic traits

BMI is the most widely used measure of obesity, and multiple MR studies have consistently suggested a positive genetically predicted association with PCOS. Using large-scale genomic data from European women, Zhao et al reported that each SD increase in BMI was associated with a 22% relative increase in PCOS risk (OR, 1.22; 95% CI, 1.15–1.31), with consistent results across IVW, weighted median, and MR-Egger methods.^[29]^ This estimate was directionally consistent with the OR of 1.15 (95% CI, 1.08–1.23) reported by Venkatesh et al in a parallel analysis of multiple obesity traits, suggesting that overall adiposity itself may substantially increase susceptibility to PCOS.^[46]^ Two-sample MR results in Asian women showed a similar trend. Yalin Zhao et al suggested that genetically predicted higher BMI was stably and positively associated with PCOS in East Asian populations, providing additional evidence for cross-ancestry consistency.^[47]^

Abdominal fat accumulation is considered a metabolically more harmful phenotype than overall obesity. In female-specific MR, each SD increase in waist-to-hip ratio (WHR) was associated with an 18% higher risk of PCOS (OR, 1.18; 95% CI, 1.06–1.32).^[29]^ Venkatesh et al further reported significant effects for both WHR and BMI-adjusted WHR, whereas their directions differed from that of hip circumference, suggesting that central fat distribution independent of BMI may have a stronger pathogenic relevance to PCOS.^[46]^ De Silva et al suggested that central obesity may explain the clinical heterogeneity of PCOS better than BMI alone and recommended combined assessment using multiple anthropometric indicators in future MR studies.^[48]^

Within the same analytical framework, the genetic effect of waist circumference did not reach statistical significance, whereas the direction of association between hip circumference and PCOS differed from that of waist circumference.^[46]^ This result suggests that abdominal and gluteofemoral fat may differ metabolically and may not contribute to PCOS in the same way.

Zhao et al^[29]^ showed that each SD increase in whole-body fat mass was associated with a 19% higher risk of PCOS (OR, 1.19; 95% CI, 1.11–1.26), and the corresponding increase for trunk fat mass was 17% (OR, 1.17; 95% CI, 1.10–1.25). These findings suggest that, beyond fat distribution, total fat mass itself should not be overlooked as a risk-related factor, and trunk fat may partly reflect visceral adiposity burden that could affect the insulin-ovarian axis.

#### 3.5.3. Anthropometric traits

A two-sample MR analysis based on large-scale GWAS summary data showed that each 1-year delay in age at menarche was associated with a significantly lower risk of PCOS. Ma et al constructed genetic instruments using 190 SNPs significantly associated with age at menarche and, after removing 4 heterogeneous loci, obtained an IVW estimate of OR = 0.84 (95% CI, 0.75–0.95; *P* = .005). Weighted median and MR-Egger estimates were directionally consistent.^[49]^ This result suggests that later activation of the reproductive axis may delay chronic low-grade inflammation related to estrogen fluctuations, thereby reducing stress on the ovarian-insulin axis at the immunological level. Notably, both Cochran *Q (P* = 9.6 × 10^−5^) and the MR-PRESSO global test (*P* = 2 × 10^−5^) indicated some heterogeneity, suggesting that different genetic pathways may contribute differently to endocrine-immune interactions.

Perinatal nutrition may influence innate immune phenotypes through developmental programming. However, after using fetal genetically predicted birthweight signals and separating maternal and fetal effects, Dong Liu et al (2023) did not observe a significant causal association in either the initial or replication MR analyses.^[50]^ Sensitivity analyses for sample overlap did not alter the conclusions, suggesting that body weight alone may be insufficient to trigger subsequent immune imbalance-ovarian dysfunction pathways. The authors speculated that associations between low birthweight and PCOS in previous observational studies may be more likely attributable to nonlinearity or environmental interactions than to a direct genetic-phenotypic causal relationship.

Childhood fat accumulation is thought to contribute to “inflammatory memory” through processes such as macrophage polarization and adipose tissue chemokine secretion. Dobbie et al integrated two-step MR with systematic evidence synthesis and analyzed genetic data from 30,016 patients with PCOS and 130,362 controls, reporting an OR of 4.99 (95% CI, 3.74–6.67) for the association between childhood obesity and adult PCOS.^[51]^ This estimate was substantially larger than that reported for adult obesity, suggesting that early adipose infiltration may exacerbate insulin-like growth factor signaling disturbances in the ovarian microenvironment through persistent pro-inflammatory cytokine networks. Rapid adolescent growth combined with sex hormone fluctuations may make immune homeostasis highly plastic. Although MR evidence for this stage remains limited, a subgroup analysis from the same project by Dobbie et al suggested an OR of 23.1 for severe adolescent obesity (BMI ≥ 40 kg/m^2^) and PCOS, compared with an OR of 3.85 for overweight, indicating a marked dose-response pattern.^[51]^ Commonly used obesity indicators in adults have also been evaluated by MR. Based on data from East Asian women, Zhao et al^[47]^ found that each 1-SD increase in BMI was associated with a 15% higher risk of PCOS (OR, 1.15; 95% CI, 1.08–1.23; *P* = 3.24 × 10^−5^). At the inflammatory-immune level, obesity-induced visceral adipose inflammation may activate the NLRP3 inflammasome and promote IL-1β release, further increasing the luteinizing hormone/follicle-stimulating hormone ratio and forming a metabolic-endocrine-immune positive feedback loop. In addition, Brower et al used bidirectional MR and found no evidence that PCOS reversely promotes generalized obesity, supporting a pathway in which obesity precedes PCOS rather than the reverse.^[52]^

Across body-size indicators at different developmental stages, childhood and adolescent obesity showed the strongest genetically supported associations with PCOS, with effect estimates much larger than those for adult obesity. This pattern is consistent with the “critical-period immune programming” hypothesis: chronic low-grade inflammation triggered by excessive early-life energy intake may have long-term effects on hematopoietic progenitor cells, mucosal immune barriers, and even the hypothalamic-pituitary-ovarian axis, thereby creating a background for later hyperandrogenism and impaired follicular development, which remains a hypothesis that warrants testing in prospective cohort or experimental studies. In contrast, birthweight did not show an independent causal effect, suggesting that reliance on body weight alone as a single morphological indicator may be insufficient to characterize fetal-origin immune-metabolic risk. In addition, the protective association of later age at menarche with PCOS again emphasizes the coupling between reproductive-axis activation and systemic inflammatory status. Later sexual maturation may not only delay the onset of insulin resistance but may also be associated with maintenance of Th1/Th2 balance before puberty, thereby reducing the inflammatory burden in the ovarian microenvironment. This finding provides a possible perspective for explaining some lean PCOS cases: even in the absence of obvious obesity, earlier menarche may increase disease risk through immune-endocrine pathways.

#### 3.5.4. Effects of other physiological traits

AMH is widely regarded as an important endocrine marker reflecting ovarian preantral follicle reserve. Harshal Deshmukh et al conducted a two-sample MR study using IVs selected from a large AMH GWAS. The IVW method showed that each SD genetically increased AMH level corresponded to a log-OR for PCOS of −0.067 (95% CI, −0.905 to 0.771; *P* = .875). Other robust methods, including weighted median and MR-Egger, showed consistent directions and no statistical significance, suggesting no causal effect of AMH on PCOS.^[45]^ The investigators further used heterogeneity testing and MR-PRESSO to assess the possibility of horizontal pleiotropy, and the results remained stable. This finding contrasts with the clinical observational pattern of “high AMH accompanying PCOS,” suggesting that AMH may be more likely a disease-associated change rather than an etiological factor.

Low serum vitamin D status often coexists with various endocrine disorders. Based on 90 significantly associated SNPs, Bingrui Gao et al performed bidirectional MR analyses of vitamin D and PCOS. Fixed-effect IVW analysis suggested that genetically increased 25 (OH) D concentrations were associated with an approximately 25% lower risk of PCOS (OR, 0.750; 95% CI, 0.587–0.959; *P* = .022). Weighted median and MR-Egger estimates were directionally consistent but not statistically significant. In multivariable MR, the protective genetically predicted association persisted after adjustment for BMI, fasting glucose, and fasting insulin, reducing the likelihood that obesity or glucose metabolic indicators fully explained the association.^[53]^ The funnel plot was symmetrical, MR-PRESSO detected no outliers, and the likelihood of horizontal pleiotropy appeared low. Overall, this study supports the possibility that adequate vitamin D status may have biological relevance for primary prevention of PCOS.

#### 3.5.5. MR analyses of metabolites and PCOS

A longitudinal comparison of the patterns and effect sizes of different metabolite classes in PCOS suggests that lipid metabolic disturbance occupies a central position across multiple studies. Ma reported that higher phosphatidylcholine and phosphatidylinositol levels were associated with a significantly increased risk of PCOS, whereas a specific sphingomyelin subtype (d38:2) showed a protective association, indicating that different subtypes within the same lipid class may have opposite effects.^[54]^ This “subtype differentiation” may help explain inconsistent findings in earlier lipidomics studies. Guo further reported significant activation of a shared metabolic network involving phosphatidylcholine and SOGPI in PCOS, suggesting that phospholipid-related pathways may influence insulin signaling through changes in the membrane lipid microenvironment.^[55]^ Wang^[56]^provided genetic evidence for an overall causal role of blood lipid metabolites in disease and noted that increased sphingolipid levels often accompanied downregulation of fatty acid pathways, supporting a bidirectional pattern of “protective sphingolipids versus risk-associated fatty acids.”

Causal evidence for amino acids and their derivatives has also expanded. Lu reported that genetically increased phenylacetic acid was significantly and positively associated with PCOS risk.^[57]^ In a large-scale metabolomic MR study, Chen identified 27 serum metabolites with protective associations and 43 with risk-increasing associations. Among them, metabolites related to the valine-leucine-isoleucine biosynthesis pathway were mostly risk-associated, supporting a link between branched-chain amino acid accumulation and insulin resistance.^[58]^

Findings for carbohydrate metabolites showed both quantitative and qualitative differences. Lu found that genetically predicted higher glucose concentrations were associated with a lower risk of PCOS, whereas glyceric acid was significantly associated with increased risk, suggesting that different nodes in glucose metabolism may have heterogeneous effects on the ovarian microenvironment.^[57]^

In addition to endogenous metabolites, exogenous compounds and their derivatives should not be overlooked. By integrating MR with toxicological evidence, Lu suggested that metabolites of endocrine-disrupting chemicals such as bisphenol A may have strong risk-increasing associations with PCOS, highlighting the importance of the environmental exposure-endocrine-metabolic axis.^[57]^

### 3.6. MR analyses of PCOS and diseases/clinical phenotypes

#### 3.6.1. Metabolism-related diseases

Regarding diabetes, although PCOS and type 2 diabetes share pathophysiological features such as marked insulin resistance, MR evidence for a direct causal relationship remains inconsistent. In a study that systematically evaluated the causal effects of PCOS on multiple diseases, Du et al did not identify a clear causal association between genetic liability to PCOS and type 2 diabetes. Gestational diabetes mellitus (GDM) also shares several features with PCOS, including insulin resistance. However, MR studies assessing the causal relationship between PCOS and GDM have produced relatively consistent findings. Guan Guixue et al did not find a definitive causal relationship between PCOS and GDM in a two-sample MR analysis. Another study by Ma et al reached a similar conclusion, showing no causal effect of PCOS on GDM occurrence (IVW-derived OR, 0.976; 95% CI, 0.904–1.053). These findings suggest that the frequent clinical co-occurrence of PCOS and GDM may not result from a direct pathogenic effect of PCOS, but may instead be driven by shared genetic backgrounds, environmental factors, or complex biological pathways.

Obesity is one of the core clinical features of PCOS and an important confounder when assessing PCOS-related comorbidities. Determining whether PCOS is a direct cause of obesity is important for understanding its long-term health risks. Du et al specifically assessed the causal effects of PCOS on different obesity classes and found no direct causal link between genetically predicted PCOS and class I obesity (BMI ≥ 30 kg/m^2^), class II obesity (BMI ≥ 35 kg/m^2^), or class III obesity (BMI ≥ 40 kg/m^2^). However, the relationship between PCOS and body mass may be more complex. In a study of PCOS and psychiatric disorders, Jiang et al (2021) found that the association between genetically instrumented PCOS and depression was likely mediated by BMI. This finding indirectly suggests that PCOS may influence other health outcomes through BMI-related pathways, which contrasts with the direct causal analyses described above.

Regarding dyslipidemia and other metabolic indicators, clinical observations suggest that patients with PCOS often exhibit an atherogenic lipid profile and insulin resistance. Although insulin resistance is considered a core pathophysiological feature of PCOS, the included MR studies did not provide clear evidence that PCOS directly increases fasting insulin or glucose levels. Hypertension, another key component of metabolic syndrome, has also received considerable attention. Du et al likewise found no causal relationship between PCOS and hypertension (OR, 0.999; 95% CI, 0.994–1.004), although substantial heterogeneity was observed in that analysis. Notably, thyroid function is closely connected with systemic metabolic status. An MR study by Zhao et al examined the effect of PCOS on thyroid function and found an association between PCOS and lower TSH levels. This finding suggests that PCOS may have a potential causal influence on the hypothalamic-pituitary-thyroid axis, whose changes may have broad systemic metabolic implications.

#### 3.6.2. CVDs

Several MR studies have broadly explored the causal relationships between PCOS and VDs. Zhang et al found no significant causal associations between genetically predicted PCOS and a range of CVDs, including atrial fibrillation, arrhythmia, coronary artery disease, heart failure, hypertension, ischemic stroke, myocardial infarction, peripheral artery disease, peripheral vascular disease, and venous thromboembolism. The study also noted that associations between PCOS and CVD observed in previous observational studies often weakened or disappeared after adjustment for BMI, further highlighting the value of MR studies for clarifying causal relationships.^[59]^

Although broad screening did not reveal clear causal links, other studies have focused more closely on specific cardiovascular endpoints. For example, Zhu et al used genetic data from UK Biobank and large international consortia, such as CARDIoGRAMplusC4D and MEGASTROKE, to specifically assess the effects of PCOS on the risk of coronary heart disease and several stroke subtypes in European populations, including any stroke, any ischemic stroke, large artery stroke, cardioembolic stroke, and small vessel stroke.^[60]^ Similarly, Simons et al (2021) conducted an MR study specifically examining the causal relationship between PCOS and coronary artery disease. This study used several rigorous sensitivity analyses, including MR-PRESSO and Steiger filtering, to comprehensively assess the robustness of its findings.^[61]^ These more targeted studies indicate ongoing interest in whether PCOS may influence key cardiovascular events through causal pathways independent of traditional metabolic risk factors.

For hypertension, the findings are more complex, particularly during pregnancy. For general hypertension, as shown in the study by Zhang et al current evidence does not support PCOS as a direct causal factor.^[59]^ However, when studies focused on hypertensive disorders of pregnancy (HDPs), MR findings were inconsistent. Ma et al found that genetic liability to PCOS was causally associated with an increased risk of HDPs, particularly gestational hypertension, but did not identify causal associations with more severe outcomes such as preeclampsia or eclampsia.^[62]^ In contrast, another MR study by Shao et al in individuals of European ancestry did not identify robust evidence for a causal effect of PCOS on HDPs. Based on this finding, the authors suggested that prevention strategies for HDPs in patients with PCOS may need to focus on identifying and intervening in individuals with high-risk features rather than applying uniform preventive measures to all patients with PCOS. The investigators also acknowledged that their conclusions may have been limited by population homogeneity, restricted to European ancestry, and by the absence of stratified analyses according to different PCOS clinical phenotypes.^[63]^ These conflicting findings underscore the complexity of the relationship between PCOS and hypertensive disorders of pregnancy and suggest that future studies should better account for ancestry differences and PCOS heterogeneity.

#### 3.6.3. Reproductive and endocrine-related phenotypes

Regarding pregnancy outcomes, Ma et al used two-sample MR to assess the causal effects of PCOS on adverse pregnancy and perinatal outcomes. The study found genetic evidence supporting an association between genetically predicted PCOS and risk of hypertensive disorders of pregnancy; however, it did not identify causal associations between PCOS and preeclampsia, eclampsia, sporadic miscarriage, or GDM.^[62]^ Runxiang Cao et al described mechanisms of endocrine dysregulation, noting that insulin resistance and compensatory hyperinsulinemia, which are common in patients with PCOS, may act on the pituitary gland to promote luteinizing hormone release and stimulate the ovaries and adrenal glands to secrete more androgens. High insulin levels may also suppress hepatic synthesis of SHBG, leading to increased free testosterone levels.^[64]^ This persistent hyperandrogenic state is a central feature of reproductive endocrine dysfunction in PCOS.

In addition to pregnancy-related complications, MR studies have begun to explore causal links between PCOS and other gynecological diseases. In a meta-analysis of MR studies, Yangming Qu et al found that PCOS may influence the risk of uterine leiomyoma, also known as uterine fibroids. Notably, their findings suggested that PCOS may be a protective factor for uterine fibroids, with an OR below 1, indicating that PCOS may be associated with a lower risk of uterine fibroids.^[65]^ This finding differs from conventional clinical assumptions and suggests a potentially complex genetic relationship between PCOS and gynecological tumors, which requires further confirmation.

#### 3.6.4. Cancer risk

Among gynecological malignancies, the association between endometrial cancer and PCOS has attracted particular attention. One targeted MR study aimed to evaluate the genetic association between PCOS and endometrial cancer risk. Chen et al emphasized that potential confounders should be considered when analyzing this relationship, including obesity, reflected by high BMI and WHR, oral contraceptive use, and reproductive status. These factors are related to the development of PCOS and also affect the risk of endometrial cancer, complicating interpretation of observational findings.^[66]^ Another study provided more detailed causal inference evidence. Kho et al found that although PCOS and endometrial cancer were genetically correlated, no direct causal association was observed after causal inference analyses accounting for obesity. The study further showed that the genetic correlation between PCOS and endometrial cancer was substantially attenuated after adjustment for the genetic component of BMI, suggesting that obesity may be a key confounder or mediator linking PCOS to endometrial cancer risk, whereas PCOS itself may not directly increase endometrial cancer risk.^[67]^

The relationship between breast cancer and PCOS is also complex, particularly because breast cancer includes distinct molecular subtypes. Wen et al noted that previous observational findings were inconsistent, partly because study designs did not adequately control for key confounders such as BMI or because heterogeneity in PCOS diagnostic criteria introduced bias, making clear causal conclusions difficult.^[68]^ MR studies provide a useful approach to clarify this issue. Wu et al provided genetic evidence for a causal association between PCOS and breast cancer risk, but the association appeared subtype-specific. Their analysis showed that genetically predicted PCOS was associated with increased risk of overall breast cancer and estrogen receptor (ER-positive) breast cancer, but not ER-negative (ER-negative) breast cancer.^[69]^ This finding was supported by subsequent research. Zhu et al similarly reported that PCOS did not increase the risk of breast cancer subtypes with poorer prognosis, such as HER2-enriched and triple-negative breast cancer, providing more refined evidence for clinical risk assessment.^[60]^ To further explore the underlying mechanisms, Cao et al used multivariable MR to examine the mediating roles of PCOS-related clinical traits in the pathway from PCOS to ER-positive breast cancer. The study found that age at menopause may have a weak mediating role, although the effect size was small and protective in direction, suggesting a potentially complex pathway through which PCOS may influence breast cancer risk via the reproductive endocrine axis.^[64]^

Regarding the causal relationship between PCOS and ovarian cancer, MR findings may differ from conventional expectations. A study by Harris et al did not find evidence that PCOS increases ovarian cancer risk; instead, it suggested a possible negative causal association. The results indicated that women with genetic liability to PCOS may have a lower risk of ovarian cancer. This potential protective association was observed across several histological subtypes, including high-grade serous, endometrioid, and clear cell ovarian cancers.^[70]^ This finding challenges the traditional view that PCOS is a risk factor for ovarian cancer and suggests that shared biological pathways may exist between the 2 conditions, although the specific mechanisms remain to be clarified.

#### 3.6.5. Other systemic effects

Traditional observational studies have frequently reported associations between PCOS and mental health problems, adverse pregnancy outcomes, and other systemic diseases. However, whether these associations are causal, and the extent to which they are influenced by confounders such as obesity and insulin resistance, remain important questions in clinical and research settings.^[59]^ In mental health, patients with PCOS are clinically observed to have higher risks of psychiatric disorders such as depression and anxiety. However, the underlying reasons for this comorbidity remain unclear. A comprehensive genetic analysis attempted to clarify the shared genetic basis and causal links between PCOS and major psychiatric disorders, including depression, schizophrenia, and bipolar disorder. Jiang et al found no shared genetic basis or direct causal relationship between PCOS and these psychiatric disorders at the genetic level. The study further noted that although genetically predicted PCOS appeared to be associated with depression, this effect was likely mediated by BMI. This suggests that the observed comorbidity may be more attributable to non-genetic exposure factors such as obesity than to a pathophysiological process directly caused by PCOS itself.^[40]^ This finding suggests that weight management may be an important intervention target when addressing mental health problems in patients with PCOS.

For patients with PCOS, pregnancy outcomes are among the major concerns during reproductive age. Several pathological features of PCOS, such as hyperandrogenism, insulin resistance, and altered endometrial receptivity, may increase the risk of adverse pregnancy and perinatal outcomes by affecting oocyte quality and placental function.^[62]^ Ma et al used two-sample MR to assess the causal effects of PCOS on multiple adverse pregnancy outcomes. The study provided genetic evidence that liability to PCOS is a causal risk factor for hypertensive disorders of pregnancy, particularly gestational hypertension. However, it did not identify significant causal associations between PCOS and preeclampsia, eclampsia, sporadic miscarriage, or GDM.^[62]^ These findings point to specific pregnancy complications for which PCOS may be relevant and provide genetic evidence to inform risk stratification and focused monitoring in pregnant women with PCOS.

The systemic effects of PCOS may also involve oral health. Some observational studies have suggested associations between PCOS and oral inflammatory diseases. To explore causality, Min et al conducted a two-sample MR study. The study found that genetically predicted PCOS was associated with increased risks of oral ulcer, gingival pain, and tooth mobility. This finding suggests that PCOS may adversely affect the oral microenvironment through chronic low-grade inflammation or other endocrine-metabolic pathways, thereby contributing to oral inflammatory diseases. This adds evidence that PCOS may affect multiple body systems and suggests that oral health may deserve consideration in the comprehensive management of patients with PCOS.^[71]^

In addition, the comorbidity of PCOS and thyroid dysfunction has attracted research attention. Clinically, patients with PCOS have been reported to have higher rates of subclinical hypothyroidism and Hashimoto thyroiditis, and their co-occurrence may exacerbate metabolic disturbances. Zhang et al noted that although genetic, immune, and endocrine hormonal factors may contribute to the comorbidity mechanisms of these 2 conditions, the exact causal relationship remains debated because of the lack of prospective cohort studies, making MR a useful tool for clarifying the underlying mechanisms.^[42]^ Together, these studies highlight that PCOS is a multifaceted and long-term endocrine disorder with effects extending beyond reproductive age and reproductive health, and that MR approaches are important for dissecting its complex etiological network.^[64]^

#### 3.6.6. Sensitivity analyses

Lim^[72]^ only excluded SNPs related to the outcome or confounders and did not perform relevant sensitivity analyses. Shao^[73]^ performed sensitivity analyses only for the MR results involving docosahexaenoic acid and PCOS and provided detailed information on the methods used, such as Cochran’s *Q* test and the MR-Egger intercept test. For other nutrients or compounds, including calcium, dehydroepiandrosterone, vitamin D, betaine, D-chiro-inositol, and metformin, the article reported only that no statistically significant causal relationships with PCOS were observed and did not mention sensitivity analyses for these results. The remaining studies performed sensitivity analyses and generally used multiple methods, including leave-one-out analysis, MR-PRESSO, and Cochran’s *Q* test.

#### 3.6.7. Development of the MR-PCOS Atlas platform

**Figure 3.**
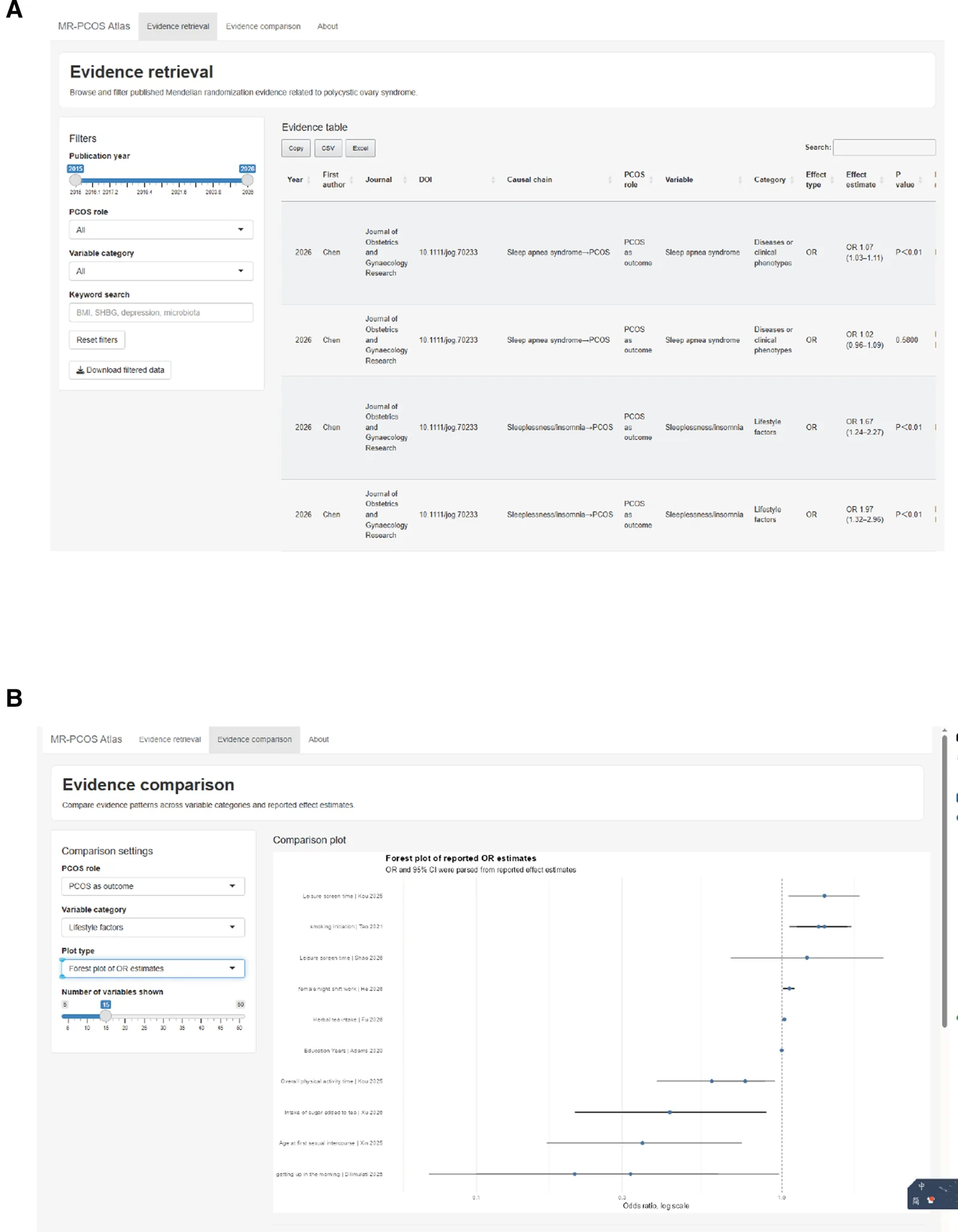
Functional demonstration of the MR-PCOS Atlas. (A) An example showing detailed study-level information from the 2025 PCOS-MR literature, including exposure, outcome, method, and effect estimates. (B) An example illustrating the causal effects of various lifestyle factors on PCOS (with PCOS as the outcome).

Using the collected data, we developed the MR-PCOS Atlas platform based on the Shiny package. Module 1 allows users to browse and filter PCOS-related MR evidence under specified conditions, as shown in Figure 3A. Module 2 allows users to compare MR results across different variable categories or different indicators in relation to PCOS, as shown in Figure 3B. The MR-PCOS Atlas is publicly accessible at http://103.236.93.60:3838/PCOS-MR/. To improve transparency and sustainability, the Shiny application code and underlying evidence data have been uploaded to GitHub at https://github.com/carifa/MR-PCOS-Atlas-Explorer. The underlying evidence data table is also provided as supplementary material to ensure availability even if the web platform is migrated or maintained. The platform is maintained by the research team. We have verified that the platform is accessible and fully functional as of July 2026. The platform is intended to be maintained continuously by the research team, and any future updates to the underlying evidence data will be documented on the GitHub repository.

## 4. Discussion

This study systematically synthesized published PCOS-related MR evidence, constructed a structured evidence map, and developed the MR-PCOS Atlas platform to support evidence retrieval, comparison, and hypothesis generation. Our analysis summarized genetic evidence on potential genetically supported relationships between multiple risk factors and PCOS, as well as the potential effects of PCOS on downstream health outcomes, thereby providing an evidence-based resource for prevention, management, and future research on this complex condition.

When MR evidence on potential antecedents was considered together, a pathophysiological axis centered on obesity-driven chronic low-grade inflammation and metabolic disturbance, followed by hyperandrogenism, became more apparent. Our synthesis supports BMI and central obesity, such as WHR, as among the most consistent and relatively strong genetically supported risk-related factors for PCOS. These associations were observed across different life stages, with childhood and adolescent obesity showing much larger effect estimates than adult obesity, highlighting the potential importance of early-life programming in PCOS development. As an initiating factor, obesity may contribute to PCOS through multiple pathways. On the 1 hand, inflammation in adipose tissue, particularly visceral adipose tissue, may activate innate immune pathways involving genes such as NLRP1 and CYBB, as well as pro-inflammatory cytokines such as CCL4, jointly creating an inflammatory microenvironment that may impair ovarian function. On the other hand, obesity may promote insulin resistance, which is consistent with findings related to IRS1 polymorphisms.

On this basis, MR studies support a protective genetically predicted association for SHBG, suggesting that it may be more than a biomarker. Genetically predicted higher SHBG levels were associated with a lower risk of PCOS, complementing findings that higher testosterone levels were associated with increased PCOS risk. Together, these findings support the central role of free androgen excess in PCOS pathophysiology and suggest that this state may be partly modulated by upstream factors such as obesity and insulin resistance. In addition, the protective association of vitamin D and the effects of several metabolites, such as specific lipids and amino acids, further refine this core axis, suggesting that the etiological network of PCOS is multidimensional and interconnected. In contrast, AMH, which has received substantial attention in observational studies, was not supported by MR evidence as a genetically supported factor, suggesting that elevated AMH may be more likely a consequence of follicular accumulation than a cause. This finding may be useful for clinical interpretation of AMH levels.

Regarding the potential consequences of PCOS, the MR evidence synthesized in this study challenges some conventional clinical assumptions. One notable finding is the limited evidence for direct genetically supported associations between PCOS and major cardiometabolic diseases such as type 2 diabetes and coronary heart disease. Several studies suggest that these associations may be largely mediated by BMI. This implies that the increased long-term cardiometabolic risk observed in patients with PCOS may be more attributable to the high prevalence of obesity than to an independent pathological effect of PCOS itself. This interpretation is important for clinical risk stratification and suggests that weight management should be prioritized when aiming to reduce long-term complications in patients with PCOS.

However, this does not imply that PCOS is a benign condition. MR evidence points to potential causal effects of PCOS on specific diseases. For example, PCOS has been genetically supported as a risk factor for ER-positive (ER-positive) breast cancer, whereas no association was observed for ER-negative breast cancer. This subtype specificity provides a new perspective for breast cancer etiology and risk screening in patients with PCOS. Similarly, the positive association between PCOS and oral inflammatory diseases, such as oral ulcers, suggests that its systemic low-grade inflammatory status may affect distant organs. More intriguingly, MR studies have suggested potentially protective associations of PCOS with ovarian cancer and uterine fibroids. Although the biological mechanisms underlying these findings remain unclear, they provide new directions for exploring shared genetic pathways among these gynecological diseases.

This study also identified inconsistent findings across MR studies in several areas, such as the relationships between PCOS and depression, thyroid disease, and hypertensive disorders of pregnancy. These inconsistencies are informative because they reflect the complexity and limitations of the current evidence. For example, conclusions regarding the genetically supported relationship between PCOS and depression differed substantially depending on whether BMI was adjusted for, strongly suggesting that obesity may act as an important mediator or confounder in the psycho-neuroendocrine axis. Similarly, differences in findings for thyroid function may arise from variation in exposure definitions, such as continuous hormone levels versus clinical diagnoses, and differences in the genetic backgrounds of study populations. These inconsistencies also indicate that future MR studies should be more refined, for example, by using multivariable Mendelian randomization to disentangle mediation effects and by conducting stratified analyses across different ancestries and PCOS clinical subtypes.

Although MR studies have unique advantages for investigating causal relationships, applying them to PCOS, a highly heterogeneous condition, remains challenging. Yaokai Wen et al emphasized that PCOS includes multiple clinical phenotypes. For example, according to the Rotterdam criteria, PCOS can be classified into classic PCOS, characterized by oligo-ovulation, hyperandrogenism, and polycystic ovarian morphology; ovulatory PCOS; and non-polycystic ovary PCOS.^[68]^ However, most GWAS data currently used in MR studies are derived from PCOS populations that combine different diagnostic criteria and clinical phenotypes.^[68]^ This heterogeneity means that current findings mainly reflect the average causal effect of PCOS as an overall construct and cannot determine whether different reproductive endocrine phenotypes, such as hyperandrogenism-predominant or ovulatory-dysfunction-predominant phenotypes, differ in their effects on related disease risks. Therefore, future genetic studies focused on distinct PCOS clinical subtypes are important for more precisely understanding the causal influence of PCOS on reproductive endocrine-related phenotypes.

The main strengths of this study are its systematic scope and methodological innovation. We not only comprehensively summarized MR evidence in the PCOS field but also developed the MR-PCOS Atlas as a practical tool, enabling a transition from static evidence synthesis to a dynamic knowledge resource and improving the accessibility and potential utility of the research findings. That said, several limitations of this work should be acknowledged. First, because our conclusions rest entirely on what has already been published, the synthesis is inevitably vulnerable to publication bias in the underlying literature. Second, the bulk of the PCOS-related GWAS data originate from European-ancestry cohorts, which constrains how far the findings can be extended to other populations, Asian cohorts in particular. Third, and as noted earlier, the GWAS resources presently in use rarely distinguish between PCOS clinical subtypes such as hyperandrogenic versus ovulatory-dysfunction phenotypes; consequently, our results capture only the average effect for PCOS as a single overall entity and may mask relationships specific to particular subtypes.

Based on the evidence synthesized in this study, several clinical implications may be considered: although these suggestions are hypothesis-generating and require prospective validation:: first, early intervention for high-risk factors related to PCOS, particularly active weight management in children and adolescents at risk of obesity; second, prioritizing weight control as a key strategy for reducing long-term cardiometabolic risk in patients with PCOS; and third, considering targeted health screening for patients with PCOS, with attention to conditions with genetically supported associations, such as ER-positive breast cancer and oral health problems.

Looking ahead, MR research in the PCOS field should develop in greater depth and breadth. Several priorities are apparent: first, large-scale PCOS GWAS in non-European populations; second, PCOS GWAS based on distinct clinical subtypes to identify subtype-specific causal pathways underlying heterogeneity; third, wider application of advanced approaches such as multivariable Mendelian randomization and bidirectional MR to disentangle complex causal networks and mediation mechanisms; and fourth, integration of MR with multi-omics data, such as proteomics and metabolomics, to validate and interpret genetic findings at the molecular level and ultimately identify potential drug targets.

## 5. Conclusions

In summary, this systematic synthesis of existing MR evidence provides a comprehensive map of the causal evidence network related to PCOS. The findings support a central role for obesity-driven inflammation and metabolic disturbance in PCOS development and help clarify the genetically supported pathways linking PCOS with several downstream conditions, while challenging some conventional assumptions. The MR-PCOS Atlas developed in this study is designed primarily for evidence retrieval and hypothesis generation rather than direct clinical decision-making. All reported genetically predicted associations require validation in experimental models and prospective clinical or interventional studies before translation into clinical practice. Future studies should focus on addressing population and phenotypic heterogeneity to support more precise prevention and treatment strategies for PCOS.

## Author contributions

**Conceptualization:** Sanwei Du, Dandan Dai, Junlin Zhou.

**Formal analysis:** Sanwei Du, Junlin Zhou.

**Methodology:** Sanwei Du, Wenhao Pan, Kexin Wang.

**Supervision:** Dandan Dai.

**Project administration:** Wenhao Pan.

**Software:** Wenhao Pan.

**Data curation:** Junlin Zhou.

**Investigation:** Kexin Wang.

**Writing – original draft:** Sanwei Du, Dandan Dai.








