# Supplementary material for: Mapping the causal landscape of polycystic ovary syndrome: A systematic Mendelian randomization evidence synthesis and the MR-PCOS atlas platform
Source: Medicine (Baltimore). 2026 Aug 21;105(34):e50366. doi: 10.1097/MD.0000000000050366 (PMC13502786; doi:10.1097/MD.0000000000050366)
Supplement: Supplementary file 1 [file medi-105-e50366-s001.docx]

| database | Search Strategy |
| --- | --- |
| PubMed | ((Ovary Syndrome, Polycystic[Title/Abstract]) OR (Syndrome, Polycystic Ovary[Title/Abstract]) OR (Polycystic Ovarian Syndrome[Title/Abstract]) OR (Ovarian Syndrome, Polycystic[Title/Abstract]) OR (Polycystic Ovary Syndrome 1[Title/Abstract]) OR (Sclerocystic Ovarian Degeneration[Title/Abstract]) OR (Ovarian Degeneration, Sclerocystic[Title/Abstract]) OR (Sclerocystic Ovary Syndrome[Title/Abstract]) OR (Stein-Leventhal Syndrome[Title/Abstract]) OR (Stein Leventhal Syndrome[Title/Abstract]) OR (Syndrome, Stein-Leventhal[Title/Abstract]) OR (Sclerocystic Ovaries[Title/Abstract]) OR (Ovary, Sclerocystic[Title/Abstract]) OR (Sclerocystic Ovary[Title/Abstract])) AND (Mendelian randomization[Title/Abstract]) |
| WOS | ((TS=(Ovary Syndrome, Polycystic)) OR TS=(Syndrome, Polycystic Ovary) OR TS=(Polycystic Ovarian Syndrome) OR TS=(Ovarian Syndrome, Polycystic) OR TS=(Polycystic Ovary Syndrome 1) OR TS=(Sclerocystic Ovarian Degeneration) OR TS=(Ovarian Degeneration, Sclerocystic) OR TS=(Sclerocystic Ovary Syndrome) OR TS=(Stein-Leventhal Syndrome) OR TS=(Stein Leventhal Syndrome) OR TS=(Syndrome, Stein-Leventhal) OR TS=(Sclerocystic Ovaries) OR TS=(Ovary, Sclerocystic) OR TS=(Sclerocystic Ovary)) AND TS=(Mendelian randomization) |
| Embase | (('Ovary Syndrome, Polycystic':ti,ab,kw OR 'Syndrome, Polycystic Ovary':ti,ab,kw OR 'Polycystic Ovarian Syndrome':ti,ab,kw OR 'Ovarian Syndrome, Polycystic':ti,ab,kw OR 'Polycystic Ovary Syndrome 1':ti,ab,kw OR 'Sclerocystic Ovarian Degeneration':ti,ab,kw OR 'Ovarian Degeneration, Sclerocystic':ti,ab,kw OR 'Sclerocystic Ovary Syndrome':ti,ab,kw OR 'Stein-Leventhal Syndrome':ti,ab,kw OR 'Stein Leventhal Syndrome':ti,ab,kw OR 'Syndrome, Stein-Leventhal':ti,ab,kw OR 'Sclerocystic Ovaries':ti,ab,kw OR 'Ovary, Sclerocystic':ti,ab,kw OR 'Sclerocystic Ovary':ti,ab,kw) AND 'Mendelian randomization':ti,ab,kw) |
| Cochrane Library | (("Ovary Syndrome, Polycystic":ti,ab,kw OR "Syndrome, Polycystic Ovary":ti,ab,kw OR "Polycystic Ovarian Syndrome":ti,ab,kw OR "Ovarian Syndrome, Polycystic":ti,ab,kw OR "Polycystic Ovary Syndrome 1":ti,ab,kw OR "Sclerocystic Ovarian Degeneration":ti,ab,kw OR "Ovarian Degeneration, Sclerocystic":ti,ab,kw OR "Sclerocystic Ovary Syndrome":ti,ab,kw OR "Stein-Leventhal Syndrome":ti,ab,kw OR "Stein Leventhal Syndrome":ti,ab,kw OR "Syndrome, Stein-Leventhal":ti,ab,kw OR "Sclerocystic Ovaries":ti,ab,kw OR "Ovary, Sclerocystic":ti,ab,kw OR "Sclerocystic Ovary":ti,ab,kw) AND "Mendelian randomization":ti,ab,kw) |
