# Supplementary material for: Mapping the causal landscape of polycystic ovary syndrome: A systematic Mendelian randomization evidence synthesis and the MR-PCOS atlas platform
Source: Medicine (Baltimore). 2026 Aug 21;105(34):e50366. doi: 10.1097/MD.0000000000050366 (PMC13502786; doi:10.1097/MD.0000000000050366)
Supplement: Supplementary file 2 [file medi-105-e50366-s002.docx]

| doi | reason |
| --- | --- |
| DOI: 10.1038/s42255-025-01444-1 | PCOS was not located at either end of the MR causal chain |
| DOI: 10.1080/14647273.2025.2610586 | PCOS was not located at either end of the MR causal chain |
| DOI: 10.1016/j.ecoenv.2026.119742 | Valid effect estimates could not be extracted |
| DOI: 10.1016/j.compbiomed.2025.111187 | PCOS was not located at either end of the MR causal chain |
| DOI: 10.1530/REP-25-0126 | Valid effect estimates could not be extracted |
| DOI: 10.1002/dmrr.70039 | Valid effect estimates could not be extracted |
| DOI: 10.1016/j.tem.2024.10.005 | Valid effect estimates could not be extracted |
| DOI: 10.1016/j.ric.2025.100008 | Valid effect estimates could not be extracted |
| DOI: 10.3389/fendo.2025.1534733 | Valid effect estimates could not be extracted |
| DOI: 10.1186/s12916-024-03795-0 | PCOS was not located at either end of the MR causal chain |
| DOI: 10.2174/0118715303266512231103075551 | Valid effect estimates could not be extracted |
| DOI: 10.1210/jendso/bvad114 | PCOS was not located at either end of the MR causal chain |
| DOI: 10.3389/fendo.2023.1133260 | PCOS was not located at either end of the MR causal chain |
| DOI: 10.1210/endrev/bnac001 | Valid effect estimates could not be extracted |
| DOI: 10.1210/jendso/bvac150 | Valid effect estimates could not be extracted |
| DOI: 10.1016/j.ebiom.2022.104154 | Valid effect estimates could not be extracted |
| DOI: 10.1016/j.tig.2022.01.005 | Valid effect estimates could not be extracted |
| DOI: 10.1016/j.fertnstert.2022.03.009 | Valid effect estimates could not be extracted |
| DOI: 10.1210/clinem/dgab757 | Valid effect estimates could not be extracted |
| DOI: 10.3389/fonc.2021.681396 | Valid effect estimates could not be extracted |
| DOI: 10.1016/j.tem.2021.05.002 | Valid effect estimates could not be extracted |
| DOI: 10.1002/edm2.267 | Valid effect estimates could not be extracted |
| DOI: 10.1016/j.ecl.2020.10.006 | Valid effect estimates could not be extracted |
| DOI: 10.3390/cancers14194817 | Valid effect estimates could not be extracted |
| DOI: 10.1136/bmjopen-2024-086717 | Valid effect estimates could not be extracted |
| DOI: 10.1136/esmoopen-2018-EACR25.598 | Valid effect estimates could not be extracted |
| DOI: 10.1210/jc.2015-3780 | Valid effect estimates could not be extracted |
| DOI: 10.1016/j.ymgme.2009.10.013 | Valid effect estimates could not be extracted |
| DOI: 10.1016/j.fertnstert.2022.03.009 | Valid effect estimates could not be extracted |
| DOI: 10.1210/jc.2015-3780 | Valid effect estimates could not be extracted |
| DOI: 10.1210/clinem/dgab757 | Valid effect estimates could not be extracted |
| DOI: 10.1016/j.tig.2022.01.005 | Valid effect estimates could not be extracted |
| DOI: 10.1016/j.tem.2021.05.002 | Valid effect estimates could not be extracted |
| DOI: 10.1016/j.ric.2025.100008 | Valid effect estimates could not be extracted |
| DOI: 10.1016/j.ecoenv.2026.119742 | Valid effect estimates could not be extracted |
| DOI: 10.1080/14647273.2025.2610586 | Valid effect estimates could not be extracted |
| DOI: 10.1016/j.ymgme.2009.10.013 | Valid effect estimates could not be extracted |
| DOI: 10.3389/fonc.2021.681396 | Valid effect estimates could not be extracted |
| DOI: 10.3390/life15050762 | Valid effect estimates could not be extracted |
| DOI: 10.1210/endrev/bnae002 | Valid effect estimates could not be extracted |
| DOI: 10.5653/cerm.2025.08543 | Valid effect estimates could not be extracted |
| DOI: 10.1530/REP-25-0126 | Valid effect estimates could not be extracted |
| DOI: 10.31083/FBL25586 | Valid effect estimates could not be extracted |
| DOI: 10.12688/wellcomeopenres.18657.2 | Valid effect estimates could not be extracted |
| DOI: 10.1371/journal.pone.0298997 | PCOS was not located at either end of the MR causal chain |
| DOI: 10.1016/j.compbiomed.2025.111187 | PCOS was not located at either end of the MR causal chain |
| DOI: 10.1097/MD.0000000000048429 | Valid effect estimates could not be extracted |
| DOI: 10.3389/fendo.2023.1133260 | Valid effect estimates could not be extracted |
| DOI: 10.1186/s40001-023-01174-8 | Valid effect estimates could not be extracted |
| DOI: 10.1002/edm2.267 | Valid effect estimates could not be extracted |
| DOI: 10.1016/j.reprotox.2025.108984 | PCOS was not located at either end of the MR causal chain |
| DOI: 10.21203/rs.3.rs-4430021/v1 | Valid effect estimates could not be extracted |
| DOI: 10.1097/MD.0000000000048611 | PCOS was not located at either end of the MR causal chain |
| DOI: 10.3389/fendo.2022.783150 | Valid effect estimates could not be extracted |
| DOI: 10.4103/singaporemedj.SMJ-2022-232 | Valid effect estimates could not be extracted |
| DOI: 10.1186/s12916-024-03795-0 | PCOS was not located at either end of the MR causal chain |
| DOI: 10.1210/clinem/dgac389 | Valid effect estimates could not be extracted |
| DOI: 10.1186/s12885-025-13774-0 | Valid effect estimates could not be extracted |
| DOI: 10.1016/j.ijcard.2016.07.174 | Valid effect estimates could not be extracted |
| DOI: 10.3389/fsurg.2025.1718589 | Valid effect estimates could not be extracted |
| DOI: 10.1016/j.ebiom.2022.104154 | Valid effect estimates could not be extracted |
| DOI: 10.3389/fendo.2024.1398600 | PCOS was not located at either end of the MR causal chain |
| DOI: 10.3389/fendo.2024.1302312 | Valid effect estimates could not be extracted |
| DOI: 10.1111/cen.12086 | Valid effect estimates could not be extracted |
| DOI: 10.1080/01443615.2024.2321321 | Valid effect estimates could not be extracted |
| DOI: 10.1038/s41562-021-01135-3 | Valid effect estimates could not be extracted |
| DOI: 10.1371/journal.pone.0304216 | PCOS was not located at either end of the MR causal chain |
| DOI: 10.1093/hmg/ddv465 | PCOS was not located at either end of the MR causal chain |
| DOI: 10.1007/s11892-019-1201-y | Valid effect estimates could not be extracted |
| DOI: 10.1530/EJE-21-0703 | PCOS was not located at either end of the MR causal chain |
| DOI: 10.3390/life15050762 | Valid effect estimates could not be extracted |
| DOI: 10.1016/j.ric.2025.100008 | Valid effect estimates could not be extracted |
| DOI: 10.3389/fsurg.2025.1718589 | Valid effect estimates could not be extracted |
| DOI: 10.21203/rs.3.rs-4430021/v1 | Valid effect estimates could not be extracted |
| DOI: 10.1186/s12916-024-03795-0 | Valid effect estimates could not be extracted |
