# Supplementary material for: Mapping the causal landscape of polycystic ovary syndrome: A systematic Mendelian randomization evidence synthesis and the MR-PCOS atlas platform
Source: Medicine (Baltimore). 2026 Aug 21;105(34):e50366. doi: 10.1097/MD.0000000000050366 (PMC13502786; doi:10.1097/MD.0000000000050366)
Supplement: Supplementary file 3 [file medi-105-e50366-s003.docx]

| **first_author** | | **year** | **journal** | **doi** | **causal_chain** | **pcos_role** | **non_pcos_variable** | **variable_category** | **effect_type** | **effect_estimate** | **effect_numeric** | **ci_lower** | **ci_upper** | **effect_method** | **p_value_label** | **p_value_numeric** | **exposure_source** | **exposure_ancestry** | **exposure_sample_size** | **outcome_source** | **outcome_ancestry** | **outcome_sample_size** | **sensitivity_methods** |
| --- | --- | --- | --- | --- | --- | --- | --- | --- | --- | --- | --- | --- | --- | --- | --- | --- | --- | --- | --- | --- | --- | --- | --- |
|  | Chen | 2026 | Journal of Obstetrics and Gynaecology Research | 10.1111/jog.70233 | Sleep apnea syndrome→PCOS | PCOS as outcome | Sleep apnea syndrome | Diseases or clinical phenotypes | OR | OR 1.07 (1.03–1.11) | 1.07 | 1.03 | -1.11 | IVW | P＜0.01 | 0.01 | IEU OpenGWAS | European | 476853 | FinnGen release 11 | European | cases/controls = 38322/216296 | Cochran Q; MR-Egger intercept; MR-PRESSO; leave-one-out; weighted median; MR-Egger |
|  | Chen | 2026 | Journal of Obstetrics and Gynaecology Research | 10.1111/jog.70233 | Sleep apnea syndrome→PCOS | PCOS as outcome | Sleep apnea syndrome | Diseases or clinical phenotypes | OR | OR 1.02 (0.96–1.09) | 1.02 | 0.96 | -1.09 | MVMR-IVW | 0.5800 | 0.58 | IEU OpenGWAS | European | 476853 | FinnGen release 11 | European | cases/controls = 38322/216296 | MVMR-Egger; MVMR-median |
|  | Chen | 2026 | Journal of Obstetrics and Gynaecology Research | 10.1111/jog.70233 | Sleeplessness/insomnia→PCOS | PCOS as outcome | Sleeplessness/insomnia | Lifestyle factors | OR | OR 1.67 (1.24–2.27) | 1.67 | 1.24 | -2.27 | IVW | P＜0.01 | 0.01 | IEU OpenGWAS / UK Biobank / MRC-IEU | European | 462341 | FinnGen release 11 | European | cases/controls = 38322/216296 | Cochran Q; MR-Egger intercept; MR-PRESSO; leave-one-out; weighted median; MR-Egger |
|  | Chen | 2026 | Journal of Obstetrics and Gynaecology Research | 10.1111/jog.70233 | Sleeplessness/insomnia→PCOS | PCOS as outcome | Sleeplessness/insomnia | Lifestyle factors | OR | OR 1.97 (1.32–2.96) | 1.97 | 1.32 | -2.96 | MVMR-IVW | P＜0.01 | 0.01 | IEU OpenGWAS / UK Biobank / MRC-IEU | European | 462341 | FinnGen release 11 | European | cases/controls = 38322/216296 | MVMR-Egger; MVMR-median |
|  | Chen | 2026 | Journal of Obstetrics and Gynaecology Research | 10.1111/jog.70233 | PCOS→sleep traits | PCOS as exposure | sleep traits | Lifestyle factors | Unclear | () |  |  |  | multiple MR methods() | Not reported |  | FinnGen release 11 | European | cases/controls = 38322/216296 | IEU OpenGWAS / UK Biobank / MRC-IEU | European |  | Cochran Q; MR-Egger intercept; MR-PRESSO; leave-one-out; weighted median; MR-Egger |
|  | Fu | 2026 | International Journal of Women's Health | 10.2147/IJWH.S576568 | CD33+ HLA-DR+ absolute count→PCOS | PCOS as outcome | CD33+ HLA-DR+ absolute count | Immune or cellular phenotypes | OR | OR 1.61 (1.27–2.03) | 1.61 | 1.27 | -2.03 | IVW | P＜0.01 | 0.01 | GWAS catalog immunophenotype datasets (GCST0001391 to GCST0002121) | European | 3735 | Apollo PCOS GWAS meta-analysis | European | cases/controls = 10074/103164 | MR-Egger intercept; Cochran Q; leave-one-out; funnel plot; weighted median |
|  | Fu | 2026 | International Journal of Women's Health | 10.2147/IJWH.S576568 | CD33+HLA-DR+ absolute count→PCOS | PCOS as outcome | CD33+ HLA-DR+ absolute count | Immune or cellular phenotypes | OR | OR 1.61 (1.27–2.03) | 1.61 | 1.27 | -2.03 | IVW | P＜0.01 | 0.01 | GWAS catalog immunophenotype datasets (GCST0001391 to GCST0002121) | European | 3735 | Apollo PCOS GWAS meta-analysis | European | cases/controls = 10074/103164 | MR-Egger intercept; Cochran Q; funnel plot; leave-one-out |
|  | Fu | 2026 | International Journal of Women's Health | 10.2147/IJWH.S576568 | Monocyte absolute count→PCOS | PCOS as outcome | Monocyte absolute count | Immune or cellular phenotypes | OR | OR 0.52 (0.39–0.69) | 0.52 | 0.39 | -0.69 | IVW | P＜0.01 | 0.01 | GWAS catalog immunophenotype datasets (GCST0001391 to GCST0002121) | European | 3735 | Apollo PCOS GWAS meta-analysis | European | cases/controls = 10074/103164 | MR-Egger intercept; MR-PRESSO; Cochran Q; leave-one-out; funnel plot; weighted median |
|  | Fu | 2026 | BMC Women's Health | 10.1186/s12905-025-04237-6 | Fresh fruit intake→Polycystic ovary syndrome | PCOS as outcome | Fresh fruit intake | Lifestyle factors | OR | OR 4.09 (1.66–10.03) | 4.09 | 1.66 | -10.03 | IVW | P＜0.01 | 0.01 | ukb-b-3881, MRC-IEU | European | 446,462 | ebi-a-GCST90044902 | European | 141,355 | MR-Egger intercept; MR-PRESSO; Cochran Q; leave-one-out; funnel plot; scatter plot |
|  | Fu | 2026 | BMC Women's Health | 10.1186/s12905-025-04237-6 | Herbal tea intake→Polycystic ovary syndrome | PCOS as outcome | Herbal tea intake | Lifestyle factors | OR | OR 1.02 (1.00–1.03) | 1.02 | 1 | -1.03 | IVW | 0.0318 | 0.0318 | ukb-b-13344, MRC-IEU | European | 64,949 | ebi-a-GCST90044902 | European | 141,355 | MR-Egger intercept; MR-PRESSO; Cochran Q; leave-one-out; funnel plot; scatter plot |
|  | Fu | 2026 | BMC Women's Health | 10.1186/s12905-025-04237-6 | Yogurt intake→Polycystic ovary syndrome | PCOS as outcome | Yogurt intake | Lifestyle factors | OR | OR 3.60 (1.58–8.21) | 3.6 | 1.58 | -8.21 | IVW | P＜0.01 | 0.01 | ukb-b-7753, MRC-IEU | European | 64,949 | ebi-a-GCST90044902 | European | 141,355 | MR-Egger intercept; MR-PRESSO; Cochran Q; leave-one-out; funnel plot; scatter plot |
|  | Fu | 2026 | BMC Women's Health | 10.1186/s12905-025-04237-6 | Fresh fruit intake→Polycystic ovary syndrome | PCOS as outcome | Fresh fruit intake | Nutritional or metabolic factors | OR | OR 4.09 (1.66–10.03) | 4.09 | 1.66 | -10.03 | IVW | 0.0020 | 0.002 | ukb-b-3881 / MRC-IEU | European | 446,462 | ebi-a-GCST90044902 | Not reported | 141,355 | MR-Egger intercept; MR-PRESSO; leave-one-out; Cochran Q |
|  | Fu | 2026 | BMC Women's Health | 10.1186/s12905-025-04237-6 | Herbal tea intake→Polycystic ovary syndrome | PCOS as outcome | Herbal tea intake | Nutritional or metabolic factors | OR | OR 1.02 (1.00–1.03) | 1.02 | 1 | -1.03 | IVW | 0.0300 | 0.03 | ukb-b-13344 / MRC-IEU | European | 64,949 | ebi-a-GCST90044902 | Not reported | 141,355 | MR-Egger intercept; MR-PRESSO; leave-one-out; Cochran Q |
|  | Fu | 2026 | BMC Women's Health | 10.1186/s12905-025-04237-6 | Yogurt intake→Polycystic ovary syndrome | PCOS as outcome | Yogurt intake | Nutritional or metabolic factors | OR | OR 3.60 (1.58–8.21) | 3.6 | 1.58 | -8.21 | IVW | 0.0020 | 0.002 | ukb-b-7753 / MRC-IEU | European | 64,949 | ebi-a-GCST90044902 | Not reported | 141,355 | MR-Egger intercept; MR-PRESSO; leave-one-out; Cochran Q |
|  | Guo | 2026 | International Journal of Women's Health | 10.2147/IJWH.S583951 | feelings of guilt→PCOS | PCOS as outcome | feelings of guilt | Diseases or clinical phenotypes | Unclear | Not reported |  |  |  | IVW | Not reported |  | relevant GWAS datasets via TwoSampleMR (Table S1) | Not reported | Not reported | GBD 2021 | Not reported | Not reported | Cochran Q; MR-Egger intercept; leave-one-out; Steiger test; funnel plot |
|  | Guo | 2026 | International Journal of Women's Health | 10.2147/IJWH.S583951 | worry or anxiety→PCOS | PCOS as outcome | worry or anxiety | Diseases or clinical phenotypes | Unclear | Not reported |  |  |  | IVW | Not reported |  | relevant GWAS datasets via TwoSampleMR (Table S1) | Not reported | Not reported | GBD 2021 | Not reported | Not reported | Cochran Q; MR-Egger intercept; leave-one-out; Steiger test; funnel plot |
|  | He | 2026 | International Journal of Biological Sciences | 10.7150/ijbs.116803 | female night shift work→PCOS | PCOS as outcome | female night shift work | Lifestyle factors | OR | OR 1.06 (1.01–1.10) | 1.06 | 1.01 | -1.1 | IVW | 0.0131 | 0.0131 | female night shift work GWAS | Not reported | Not reported | GWAS cohort in Finland | Finnish | cases/controls = 31548/179322 | MR-Egger; weighted median; weighted mode; MR-PRESSO; Cochran Q; I²; leave-one-out; funnel plot; scatter plot |
|  | He | 2026 | International Journal of Biological Sciences | 10.7150/ijbs.116803 | Female night shift work→PCOS | PCOS as outcome | female night shift work | Lifestyle factors | OR | OR 1.06 (1.01–1.10) | 1.06 | 1.01 | -1.1 | IVW | 0.0131 | 0.0131 | (female night shift work GWAS, ) | Not reported | Not reported | GWAS cohort in Finland | Finnish | cases/controls = 31548/179322 | MR-Egger; weighted median; weighted mode; MR-PRESSO; Cochran Q; I²; leave-one-out; funnel plot; scatter plot |
|  | He | 2026 | International Journal of Biological Sciences | 10.7150/ijbs.116803 | serum leucine levels→PCOS | PCOS as outcome | serum leucine levels | Nutritional or metabolic factors | OR | OR 1.12 (1.01–1.24) | 1.12 | 1.01 | -1.24 | IVW | 0.0268 | 0.0268 | IEU Open GWAS | Not reported | 115,047 | GWAS cohort in Finland | Finnish | cases/controls = 31548/179322 | MR-Egger; weighted median; weighted mode; MR-PRESSO; Cochran Q; I²; leave-one-out; funnel plot; scatter plot |
|  | He | 2026 | International Journal of Biological Sciences | 10.7150/ijbs.116803 | PCOS→serum leucine levels | PCOS as exposure | serum leucine levels | Nutritional or metabolic factors | Beta | β 0.03 (0.02–0.05) | 0.03 | 0.02 | -0.05 | IVW | P＜0.01 | 0.01 | GWAS cohort in Finland | Finnish | cases/controls = 31548/179322 | IEU Open GWAS | Not reported | 115,047 | MR-Egger; weighted median; weighted mode; MR-PRESSO; Cochran Q; I²; leave-one-out |
|  | He | 2026 | International Journal of Biological Sciences | 10.7150/ijbs.116803 | Serum leucine levels→PCOS | PCOS as outcome | serum leucine levels | Nutritional or metabolic factors | OR | OR 1.12 (1.01–1.24) | 1.12 | 1.01 | -1.24 | IVW | 0.0268 | 0.0268 | IEU Open GWAS | Not reported | 115,047 | GWAS cohort in Finland | Finnish | cases/controls = 31548/179322 | MR-Egger; weighted median; weighted mode; MR-PRESSO; Cochran Q; I²; leave-one-out; funnel plot; scatter plot |
|  | He | 2026 | International Journal of Biological Sciences | 10.7150/ijbs.116803 | PCOS→Serum leucine levels | PCOS as exposure | serum leucine levels | Nutritional or metabolic factors | Beta | β 0.03 (0.02–0.05) | 0.03 | 0.02 | -0.05 | IVW | P＜0.01 | 0.01 | GWAS cohort in Finland | Finnish | cases/controls = 31548/179322 | IEU Open GWAS | Not reported | 115,047 | MR-Egger; weighted median; weighted mode; MR-PRESSO; Cochran Q; I²; leave-one-out; funnel plot; scatter plot |
|  | He | 2026 | Journal of Ovarian Research | 10.1186/s13048-026-02014-z | ACOX2 expression→PCOS | PCOS as outcome | ACOX2 expression | Proteins or genes | OR | OR 0.39 (0.18–0.85) | 0.39 | 0.18 | -0.85 | SMR | 0.0173 | 0.0173 | eQTLGen | Not reported | 31684 | FinnGen_R11_E4_PCOS | European | cases/controls = 1909/241998 | HEIDI; colocalization analysis |
|  | He | 2026 | Journal of Ovarian Research | 10.1186/s13048-026-02014-z | GLIPR1 expression→PCOS | PCOS as outcome | GLIPR1 expression | Proteins or genes | OR | OR 0.86 (0.75–0.98) | 0.86 | 0.75 | -0.98 | SMR | 0.0285 | 0.0285 | eQTLGen | Not reported | 31684 | FinnGen_R11_E4_PCOS | European | cases/controls = 1909/241998 | HEIDI; colocalization analysis |
|  | He | 2026 | Journal of Ovarian Research | 10.1186/s13048-026-02014-z | PIK3R3 expression→PCOS | PCOS as outcome | PIK3R3 expression | Proteins or genes | OR | OR 1.39 (1.07–1.79) | 1.39 | 1.07 | -1.79 | SMR | 0.0120 | 0.012 | eQTLGen | Not reported | 31684 | FinnGen_R11_E4_PCOS | European | cases/controls = 1909/241998 | HEIDI; colocalization analysis |
|  | He | 2026 | Journal of Ovarian Research | 10.1186/s13048-026-02014-z | cg01554451 methylation (GLIPR1)→PCOS | PCOS as outcome | cg01554451 methylation (GLIPR1) | Proteins or genes | OR | Not reported |  |  |  | SMR | Not reported |  | blood mQTL meta-analysis (Brisbane Systems Genetics Study + Lothian Birth Cohorts) | European | 1980 | FinnGen_R11_E4_PCOS | European | cases/controls = 1909/241998 | HEIDI; colocalization analysis |
|  | He | 2026 | Journal of Ovarian Research | 10.1186/s13048-026-02014-z | cg22012981 methylation (ACOX2)→PCOS | PCOS as outcome | cg22012981 methylation (ACOX2) | Proteins or genes | OR | Not reported |  |  |  | SMR | Not reported |  | blood mQTL meta-analysis (Brisbane Systems Genetics Study + Lothian Birth Cohorts) | European | 1980 | FinnGen_R11_E4_PCOS | European | cases/controls = 1909/241998 | HEIDI; colocalization analysis |
|  | He | 2026 | Journal of Ovarian Research | 10.1186/s13048-026-02014-z | cg27584146 methylation (PIK3R3)→PCOS | PCOS as outcome | cg27584146 methylation (PIK3R3) | Proteins or genes | OR | Not reported |  |  |  | SMR | Not reported |  | blood mQTL meta-analysis (Brisbane Systems Genetics Study + Lothian Birth Cohorts) | European | 1980 | FinnGen_R11_E4_PCOS | European | cases/controls = 1909/241998 | HEIDI; colocalization analysis |
|  | Jiang | 2026 | Ecotoxicology and Environmental Safety | 10.1016/j.ecoenv.2026.119900 | FAM19A2→PCOS | PCOS as outcome | FAM19A2 | Proteins or genes | OR | Not reported |  |  |  | IVW | P＜0.01 | 0.01 | FinnGen | Not reported | 31,470 | FinnGen | Not reported | cases/controls = 4810/273021 | MR-Egger; weighted median; simple mode; weighted mode; MR-Egger intercept; Cochran Q |
|  | Jiang | 2026 | Ecotoxicology and Environmental Safety | 10.1016/j.ecoenv.2026.119900 | KCTD15→PCOS | PCOS as outcome | KCTD15 | Proteins or genes | OR | Not reported |  |  |  | IVW | P＜0.01 | 0.01 | FinnGen | Not reported | 30,935 | FinnGen | Not reported | cases/controls = 4810/273021 | MR-Egger; weighted median; simple mode; weighted mode; MR-Egger intercept; Cochran Q |
|  | Li | 2026 | Journal of Ovarian Research | 10.1186/s13048-026-01970-w | PCOS→MDD | PCOS as exposure | major depressive disorder | Diseases or clinical phenotypes | Unclear | Not reported |  |  |  | IVW | Not reported |  | FinnGen R12 | European | cases/controls = 42630/239434 | PGC | European | cases/controls = 246363/561190 | MR-Egger intercept; Cochran Q; MR-PRESSO; weighted median; maximum likelihood |
|  | Li | 2026 | Journal of Ovarian Research | 10.1186/s13048-026-01970-w | MDD→PCOS | PCOS as outcome | major depressive disorder | Diseases or clinical phenotypes | Unclear | () |  |  |  | multiple MR methods() | Not reported |  | PGC | European | cases/controls = 246363/561190 | FinnGen R12 | European | cases/controls = 42630/239434 | MR-Egger intercept; Cochran Q; MR-PRESSO; weighted median; maximum likelihood |
|  | Li | 2026 | Journal of Ovarian Research | 10.1186/s13048-026-01970-w | PCOS→psychiatric disorders | PCOS as exposure | psychiatric disorders | Diseases or clinical phenotypes | Unclear | () |  |  |  | multiple MR methods() | Not reported |  | FinnGen R12 | European | cases/controls = 42630/239434 | Psychiatric Genomics Consortium (PGC) | European |  | MR-Egger intercept; Cochran Q; MR-PRESSO; weighted median; maximum likelihood |
|  | Li | 2026 | Journal of Ovarian Research | 10.1186/s13048-026-01970-w | psychiatric disorders→PCOS | PCOS as outcome | psychiatric disorders | Diseases or clinical phenotypes | Unclear | () |  |  |  | IVW | Not reported |  | Psychiatric Genomics Consortium (PGC) | European |  | FinnGen R12 | European | cases/controls = 42630/239434 | MR-Egger intercept; Cochran Q; MR-PRESSO; weighted median; maximum likelihood |
|  | Lu | 2026 | International Journal of Women's Health | 10.2147/IJWH.S593314 | Nitrogen dioxide air pollution→Polycystic ovary syndrome | PCOS as outcome | Nitrogen dioxide air pollution | Other | OR | OR 6.60 (1.85–23.58) | 6.6 | 1.85 | -23.58 | IVW | P＜0.01 | 0.01 | IEU OpenGWAS ukb-b-2618 | European | 456,380 | IEU OpenGWAS ebia-GCST90044902 | European | cases/controls = 797/140,558 | MR-Egger intercept; MR-PRESSO global test; Cochran Q; leave-one-out; weighted median; weighted mode; simple mode; Steiger test; funnel plot; scatter plot |
|  | Lu | 2026 | International Journal of Women's Health | 10.2147/IJWH.S593314 | Nitrogen dioxide air pollution; 2005→Polycystic ovary syndrome | PCOS as outcome | Nitrogen dioxide air pollution; 2005 | Other | OR | OR 6.60 (1.85–23.58) | 6.6 | 1.85 | -23.58 | IVW | P＜0.01 | 0.01 | IEU OpenGWAS ukb-b-2618 | European | 456,380 | IEU OpenGWAS ebia-GCST90044902 | European | cases/controls = 797/140,558 | MR-Egger intercept; MR-PRESSO global test; Cochran Q; Steiger test; leave-one-out; funnel plot; weighted median; weighted mode; simple mode |
|  | Luo | 2026 | International Journal of Gynecology and Obstetrics | 10.1002/ijgo.70897 | Gestational hypertension→Polycystic ovary syndrome | PCOS as outcome | gestational hypertension | Diseases or clinical phenotypes | OR | OR 1.12 (0.73–1.74) | 1.12 | 0.73 | -1.74 | IVW | 0.5790 | 0.579 | IEU Open GWAS project, finn-b-O15_HYPTENSPREG | European | 123579 | IEU Open GWAS project | European | Not reported | MR-Egger intercept; weighted median; weighted mode; simple mode; Cochran Q; leave-one-out; funnel plot; scatter plot |
|  | Moolhuijsen | 2026 | Nature Genetics | 10.1038/s41588-026-02543-9 | PCOS→asthma | PCOS as exposure | asthma | Diseases or clinical phenotypes | Unclear | () |  |  |  | multiple MR methods() | Not reported |  | PCOS GWAS three-strata meta-analysis | mixed ancestry | cases/controls = 20818/523695 | most recent genome-wide study for each outcome | Not reported | Not reported | MR-Egger intercept; I2 |
|  | Moolhuijsen | 2026 | Nature Genetics | 10.1038/s41588-026-02543-9 | PCOS→depression | PCOS as exposure | depression | Diseases or clinical phenotypes | Unclear | () |  |  |  | multiple MR methods() | Not reported |  | PCOS GWAS three-strata meta-analysis | mixed ancestry | cases/controls = 20818/523695 | most recent genome-wide study for each outcome | Not reported | Not reported | MR-Egger intercept; I2 |
|  | Moolhuijsen | 2026 | Nature Genetics | 10.1038/s41588-026-02543-9 | PCOS→age at natural menopause | PCOS as exposure | age at natural menopause | Physiological traits | Unclear | () |  |  |  | multiple MR methods() | Not reported |  | PCOS GWAS three-strata meta-analysis | mixed ancestry | cases/controls = 20818/523695 | publicly available GWAS dataset for age at natural menopause | Not reported | Not reported | MR-Egger intercept; I2 |
|  | Moolhuijsen | 2026 | Nature Genetics | 10.1038/s41588-026-02543-9 | age at natural menopause→PCOS | PCOS as outcome | age at natural menopause | Physiological traits | Unclear | () |  |  |  | multiple MR methods() | Not reported |  | Age at natural menopause GWAS | Not reported | Not reported | PCOS GWAS three-strata meta-analysis | mixed ancestry | cases/controls = 20818/523695 | MR-Egger intercept; I2 |
|  | Moolhuijsen | 2026 | Nature Genetics | 10.1038/s41588-026-02543-9 | age at natural menopause→PCOS | PCOS as outcome | age at natural menopause (DNA-repair pathway variants) | Physiological traits | Unclear | () |  |  |  | IVW | Not reported |  | reported variants for age at natural menopause stratified by DNA-repair pathway annotation | Not reported | Not reported | PCOS GWAS three-strata meta-analysis | mixed ancestry | cases/controls = 20818/523695 | MR-Egger intercept; I2 |
|  | Moolhuijsen | 2026 | Nature Genetics | 10.1038/s41588-026-02543-9 | age at natural menopause→PCOS | PCOS as outcome | age at natural menopause (non-DNA-repair pathway variants) | Physiological traits | Unclear | () |  |  |  | IVW | Not reported |  | reported variants for age at natural menopause stratified by DNA-repair pathway annotation | Not reported | Not reported | PCOS GWAS three-strata meta-analysis | mixed ancestry | cases/controls = 20818/523695 | MR-Egger intercept; I2 |
|  | Shao | 2026 | Research | 10.34133/research.1131 | Leisure screen time→Polycystic ovarian syndrome | PCOS as outcome | Leisure screen time | Lifestyle factors | OR | OR 3.05 (1.65–5.65) | 3.05 | 1.65 | -5.65 | IVW | P＜0.01 | 0.01 | UK Biobank sex-stratified GWAS | European | 462,399 | Large-scale GWAS meta-analysis of PCOS | European | cases/controls = 10,074/103,164 | MR-RAPS; MR-ROBUST; dIVW; MR-Egger intercept; MR-PRESSO; Cochran Q; leave-one-out; Steiger test |
|  | Shao | 2026 | Research | 10.34133/research.1131 | Leisure screen time→Polycystic ovarian syndrome | PCOS as outcome | Leisure screen time | Lifestyle factors | OR | OR 1.21 (0.68–2.15) | 1.21 | 0.68 | -2.15 | MVMR-IVW | 0.5200 | 0.52 | UK Biobank sex-stratified GWAS | European | 462,399 | Large-scale GWAS meta-analysis of PCOS | European | cases/controls = 10,074/103,164 | MVMR-Egger; Robust MV-IVW; MV-weighted median |
|  | Shao | 2026 | Research | 10.34133/research.1131 | Leisure screen time→Polycystic ovarian syndrome | PCOS as outcome | Leisure screen time | Lifestyle factors | OR | OR 1.42 (1.09–1.85) | 1.42 | 1.09 | -1.85 | IVW | P＜0.01 | 0.01 | UK Biobank sex-stratified GWAS | European | 462,399 | Recent GWAS meta-analysis of reproductive health disorders combining Estonian Biobank and FinnGen | European | up to 293,618 women participants | MR-ROBUST; dIVW; MR-RAPS |
|  | Shao | 2026 | Research | 10.34133/research.1131 | Body mass index→Polycystic ovarian syndrome | PCOS as outcome | Body mass index | Physiological traits | Unclear | () |  |  |  | multiple MR methods() | Not reported |  | female-specific GWAS of BMI from the GIANT consortium | European | Not reported | Large-scale GWAS meta-analysis of PCOS | European | cases/controls = 10,074/103,164 | () |
|  | Wang | 2026 | Medicine | 10.1097/MD.0000000000048704 | PCOS→IgA nephropathy | PCOS as exposure | IgA nephropathy | Diseases or clinical phenotypes | OR | OR 1.05 (0.99–1.11) | 1.05 | 0.99 | -1.11 | IVW | 0.1254 | 0.1254 | Finngen_R9_E4_PCOS | European | cases/controls = 1424/392423 | ebi-a-GCST90018866 | European | cases/controls = 15587/462197 | MR-Egger intercept; Cochran Q; leave-one-out; weighted median; MR-Egger; funnel plot |
|  | Wang | 2026 | Medicine | 10.1097/MD.0000000000048704 | PCOS→IgA nephropathy | PCOS as exposure | IgA nephropathy | Diseases or clinical phenotypes | OR | OR 1.02 (0.99–1.06) | 1.02 | 0.99 | -1.06 | IVW | 0.2416 | 0.2416 | PCOS summary statistics | European | cases/controls = 10074/103164 | ebi-a-GCST90018866 | European | cases/controls = 15587/462197 | MR-Egger intercept; Cochran Q; leave-one-out; weighted median; MR-Egger; funnel plot |
|  | Wang | 2026 | Medicine | 10.1097/MD.0000000000048704 | IgA nephropathy→PCOS | PCOS as outcome | IgA nephropathy | Diseases or clinical phenotypes | OR | OR 1.44 (0.99–2.09) | 1.44 | 0.99 | -2.09 | IVW | 0.0558 | 0.0558 | ebi-a-GCST90018866 | European | cases/controls = 15587/462197 | Finngen_R9_E4_PCOS | European | cases/controls = 1424/392423 | MR-Egger intercept; Cochran Q; leave-one-out; weighted median; MR-Egger; funnel plot |
|  | Wang | 2026 | Medicine | 10.1097/MD.0000000000048704 | IgA nephropathy→PCOS | PCOS as outcome | IgA nephropathy | Diseases or clinical phenotypes | OR | OR 1.12 (0.54–2.30) | 1.12 | 0.54 | -2.3 | IVW | 0.7599 | 0.7599 | ebi-a-GCST90018866 | European | cases/controls = 15587/462197 | PCOS summary statistics | European | cases/controls = 10074/103164 | MR-Egger intercept; Cochran Q; leave-one-out; weighted median; MR-Egger; funnel plot |
|  | Wang | 2026 | International Journal of Endocrinology | 10.1155/ije/3604933 | Obstructive sleep apnea→Polycystic ovary syndrome | PCOS as outcome | Obstructive sleep apnea | Diseases or clinical phenotypes | OR | OR 1.34 (1.01–1.77) | 1.34 | 1.01 | -1.77 | IVW | 0.0390 | 0.039 | FinnGen release 11 | European | cases/controls = 54698/399035 | GWAS catalog GCST90044902 | European | cases/controls = 3609/229788 | MR-Egger intercept; MR-PRESSO; Cochran Q; leave-one-out; weighted median; scatter plot |
|  | Wang | 2026 | International Journal of Endocrinology | 10.1155/ije/3604933 | Polycystic ovary syndrome→Obstructive sleep apnea | PCOS as exposure | Obstructive sleep apnea | Diseases or clinical phenotypes | OR | OR 1.08 (0.95–1.24) | 1.08 | 0.95 | -1.24 | IVW | 0.2450 | 0.245 | GWAS catalog GCST90044902 | European | cases/controls = 3609/229788 | FinnGen release 11 | European | cases/controls = 54698/399035 | MR-Egger intercept; MR-PRESSO; Cochran Q; leave-one-out; weighted median; scatter plot |
|  | Wang | 2026 | International Journal of Endocrinology | 10.1155/ije/3604933 | Obstructive sleep apnea→Polycystic ovary syndrome | PCOS as outcome | obstructive sleep apnea | Diseases or clinical phenotypes | OR | OR 1.34 (1.01–1.77) | 1.34 | 1.01 | -1.77 | IVW | 0.0390 | 0.039 | FinnGen release 11 | European | cases/controls = 54698/399035 | GWAS Catalog GCST90044902 | European | cases/controls = 3609/229788 | MR-Egger intercept; MR-PRESSO; Cochran Q; leave-one-out; weighted median; scatter plot |
|  | Wang | 2026 | International Journal of Endocrinology | 10.1155/ije/3604933 | Polycystic ovary syndrome→Obstructive sleep apnea | PCOS as exposure | obstructive sleep apnea | Diseases or clinical phenotypes | OR | OR 1.08 (0.95–1.24) | 1.08 | 0.95 | -1.24 | IVW | 0.2450 | 0.245 | GWAS Catalog GCST90044902 | European | cases/controls = 3609/229788 | FinnGen release 11 | European | cases/controls = 54698/399035 | MR-Egger intercept; MR-PRESSO; Cochran Q; leave-one-out; weighted median; scatter plot |
|  | Wang | 2026 | International Journal of Endocrinology | 10.1155/ije/3604933 | Body mass index→Polycystic ovary syndrome | PCOS as outcome | Body mass index | Physiological traits | OR | OR 1.48 (1.15–1.90) | 1.48 | 1.15 | -1.9 | IVW | P＜0.01 | 0.01 | UK Biobank and GIANT consortium | European | 434794 female individuals | GWAS catalog GCST90044902 | European | cases/controls = 3609/229788 | MR-Egger intercept; MR-PRESSO; Cochran Q; leave-one-out; weighted median; scatter plot |
|  | Wang | 2026 | International Journal of Endocrinology | 10.1155/ije/3604933 | Body mass index→Polycystic ovary syndrome | PCOS as outcome | body mass index | Physiological traits | OR | OR 1.48 (1.15–1.90) | 1.48 | 1.15 | -1.9 | IVW | P＜0.01 | 0.01 | UK Biobank and GIANT consortium | European | 434794 female individuals | GWAS Catalog GCST90044902 | European | cases/controls = 3609/229788 | MR-Egger intercept; MR-PRESSO; Cochran Q; leave-one-out; weighted median; scatter plot |
|  | Xiao | 2026 | Naunyn-Schmiedeberg's Archives of Pharmacology | 10.1007/s00210-026-05473-9 | CAMKK1→PCOS | PCOS as outcome | CAMKK1 | Proteins or genes | OR | OR 0.65 (0.51–0.84) | 0.65 | 0.51 | -0.84 | IVW | P＜0.01 | 0.01 | UK Biobank Pharma Proteomics Project (UKB-PPP) | Not reported | 54219 | FinnGen | European | cases/controls = 642/118228 | MR-Egger intercept; Cochran Q; replication analysis |
|  | Xiao | 2026 | Naunyn-Schmiedeberg's Archives of Pharmacology | 10.1007/s00210-026-05473-9 | ERBB4→PCOS | PCOS as outcome | ERBB4 | Proteins or genes | OR | OR 1.92 (1.07–3.46) | 1.92 | 1.07 | -3.46 | IVW | 0.0280 | 0.028 | UK Biobank Pharma Proteomics Project (UKB-PPP) | Not reported | 54219 | FinnGen | European | cases/controls = 642/118228 | MR-Egger intercept; Cochran Q; replication analysis |
|  | Xiao | 2026 | Naunyn-Schmiedeberg's Archives of Pharmacology | 10.1007/s00210-026-05473-9 | FSHB→PCOS | PCOS as outcome | FSHB | Proteins or genes | OR | OR 0.27 (0.15–0.48) | 0.27 | 0.15 | -0.48 | IVW | P＜0.01 | 0.01 | UK Biobank Pharma Proteomics Project (UKB-PPP) | Not reported | 54219 | FinnGen | European | cases/controls = 642/118228 | MR-Egger intercept; Cochran Q; replication analysis |
|  | Xiao | 2026 | Naunyn-Schmiedeberg's Archives of Pharmacology | 10.1007/s00210-026-05473-9 | PDLIM4→PCOS | PCOS as outcome | PDLIM4 | Proteins or genes | OR | OR 1.69 (1.03–2.75) | 1.69 | 1.03 | -2.75 | Wald ratio | 0.0360 | 0.036 | deCODE Genetics / Icelandic dataset | Icelandic | 35559 | FinnGen | European | cases/controls = 642/118228 | MR-Egger intercept; Cochran Q; replication analysis |
|  | Xiao | 2026 | Naunyn-Schmiedeberg's Archives of Pharmacology | 10.1007/s00210-026-05473-9 | THOP1→PCOS | PCOS as outcome | THOP1 | Proteins or genes | OR | OR 0.64 (0.49–0.84) | 0.64 | 0.49 | -0.84 | Wald ratio | P＜0.01 | 0.01 | UK Biobank Pharma Proteomics Project (UKB-PPP) | Not reported | 54219 | FinnGen | European | cases/controls = 642/118228 | MR-Egger intercept; Cochran Q; replication analysis |
|  | Xu | 2026 | International Journal of Women's Health | 10.2147/IJWH.S586268 | Alcohol intake frequency→PCOS | PCOS as outcome | Alcohol intake frequency | Lifestyle factors | OR | OR 1.39 (1.03–1.88) | 1.39 | 1.03 | -1.88 | IVW | 0.0300 | 0.03 | UK Biobank | European | Not reported | FinnGen cohort (GWAS ID FINNGEN_R12_E4_PCOS) | European | cases/controls = 2214/267780 | weighted median; weighted mode; MR-Egger intercept; MR-PRESSO; Cochran Q; leave-one-out; funnel plot; scatter plot; forest plot |
|  | Xu | 2026 | International Journal of Women's Health | 10.2147/IJWH.S586268 | Alcohol intake frequency→Polycystic ovary syndrome | PCOS as outcome | Alcohol intake frequency | Lifestyle factors | OR | OR 1.39 (1.03–1.88) | 1.39 | 1.03 | -1.88 | IVW | 0.0300 | 0.03 | UK Biobank | European | Not reported | FinnGen cohort (GWAS ID FINNGEN_R12_E4_PCOS) | European | cases/controls = 2214/267780 | weighted median; weighted mode; MR-Egger intercept; MR-PRESSO; Cochran Q; leave-one-out; funnel plot; scatter plot; forest plot |
|  | Xu | 2026 | International Journal of Women's Health | 10.2147/IJWH.S586268 | Intake of sugar added to tea→PCOS | PCOS as outcome | Intake of sugar added to tea | Lifestyle factors | OR | OR 0.43 (0.21–0.89) | 0.43 | 0.21 | -0.89 | IVW | 0.0220 | 0.022 | UK Biobank | European | Not reported | FinnGen cohort (GWAS ID FINNGEN_R12_E4_PCOS) | European | cases/controls = 2214/267780 | weighted median; weighted mode; MR-Egger intercept; MR-PRESSO; Cochran Q; leave-one-out; funnel plot; scatter plot; forest plot |
|  | Xu | 2026 | International Journal of Women's Health | 10.2147/IJWH.S586268 | Intake of sugar added to tea→Polycystic ovary syndrome | PCOS as outcome | Intake of sugar added to tea | Lifestyle factors | OR | OR 0.43 (0.21–0.89) | 0.43 | 0.21 | -0.89 | IVW | 0.0220 | 0.022 | UK Biobank | European | Not reported | FinnGen cohort (GWAS ID FINNGEN_R12_E4_PCOS) | European | cases/controls = 2214/267780 | weighted median; weighted mode; MR-Egger intercept; MR-PRESSO; Cochran Q; leave-one-out; funnel plot; scatter plot; forest plot |
|  | Xu | 2026 | Front. Med. | 10.3389/fmed.2026.1747593 | 3-hydroxybutyrate→PCOS | PCOS as outcome | 3-hydroxybutyrate | Metabolites | OR | OR 1.55 (95% CI ) | 1.55 |  |  | IVW | 0.0230 | 0.023 | Karjalainen et al. metabolite GWAS | Not reported | Not reported | EBI database (ID: ebi-a-GCST90044902) | Not reported | Not reported | MR-Egger intercept; Cochran Q; leave-one-out; weighted median; scatter plot; funnel plot; forest plot |
|  | Xu | 2026 | Frontiers in Medicine | 10.3389/fmed.2026.1747593 | Concentration of extremely small VLDL particles→PCOS | PCOS as outcome | Concentration of extremely small VLDL particles | Metabolites | OR | OR 1.16 (95% CI ) | 1.16 |  |  | IVW | 0.0380 | 0.038 | Karjalainen et al. metabolite GWAS / IEU | Not reported | Not reported | EBI database (ID: ebi-a-GCST90044902) | Not reported | Not reported | weighted median; MR-Egger; Cochran Q; MR-Egger intercept; leave-one-out; scatter plot; funnel plot; forest plot |
|  | Xu | 2026 | Front. Med. | 10.3389/fmed.2026.1747593 | Particle number in extremely small VLDL→PCOS | PCOS as outcome | Particle number in extremely small VLDL | Metabolites | OR | OR 1.16 (95% CI ) | 1.16 |  |  | IVW | 0.0380 | 0.038 | Karjalainen et al. metabolite GWAS | Not reported | Not reported | EBI database (ID: ebi-a-GCST90044902) | Not reported | Not reported | MR-Egger intercept; Cochran Q; leave-one-out; weighted median; scatter plot; funnel plot; forest plot |
|  | Xu | 2026 | Frontiers in Medicine | 10.3389/fmed.2026.1747593 | Ratio of apolipoprotein B to A1→PCOS | PCOS as outcome | Ratio of apolipoprotein B to A1 | Metabolites | OR | OR 1.15 (95% CI ) | 1.15 |  |  | IVW | 0.0400 | 0.04 | Karjalainen et al. metabolite GWAS / IEU | Not reported | Not reported | EBI database (ID: ebi-a-GCST90044902) | Not reported | Not reported | weighted median; MR-Egger; Cochran Q; MR-Egger intercept; leave-one-out; scatter plot; funnel plot; forest plot |
|  | Xu | 2026 | Front. Med. | 10.3389/fmed.2026.1747593 | Ratio of triglycerides to total lipids in large LDL→PCOS | PCOS as outcome | Ratio of triglycerides to total lipids in large LDL | Metabolites | OR | OR 1.26 (95% CI ) | 1.26 |  |  | IVW | 0.0190 | 0.019 | Karjalainen et al. metabolite GWAS | Not reported | Not reported | EBI database (ID: ebi-a-GCST90044902) | Not reported | Not reported | MR-Egger intercept; Cochran Q; leave-one-out; weighted median; scatter plot; funnel plot; forest plot |
|  | Xu | 2026 | Front. Med. | 10.3389/fmed.2026.1747593 | Total cholesterol levels in very large HDL→PCOS | PCOS as outcome | Total cholesterol levels in very large HDL | Metabolites | OR | OR 0.82 (95% CI ) | 0.82 |  |  | IVW | 0.0390 | 0.039 | Karjalainen et al. metabolite GWAS | Not reported | Not reported | EBI database (ID: ebi-a-GCST90044902) | Not reported | Not reported | MR-Egger intercept; Cochran Q; leave-one-out; weighted median; scatter plot; funnel plot; forest plot |
|  | Xu | 2026 | Front. Med. | 10.3389/fmed.2026.1747593 | Total lipids in extremely small VLDL→PCOS | PCOS as outcome | Total lipids in extremely small VLDL | Metabolites | OR | OR 1.16 (95% CI ) | 1.16 |  |  | IVW | 0.0460 | 0.046 | Karjalainen et al. metabolite GWAS | Not reported | Not reported | EBI database (ID: ebi-a-GCST90044902) | Not reported | Not reported | MR-Egger intercept; Cochran Q; leave-one-out; weighted median; scatter plot; funnel plot; forest plot |
|  | Xu | 2026 | Front. Med. | 10.3389/fmed.2026.1747593 | Triglyceride in medium LDL→PCOS | PCOS as outcome | Triglyceride in medium LDL | Metabolites | OR | OR 1.18 (95% CI ) | 1.18 |  |  | IVW | 0.0370 | 0.037 | Karjalainen et al. metabolite GWAS | Not reported | Not reported | EBI database (ID: ebi-a-GCST90044902) | Not reported | Not reported | MR-Egger intercept; Cochran Q; leave-one-out; weighted median; scatter plot; funnel plot; forest plot |
|  | Xu | 2026 | Front. Med. | 10.3389/fmed.2026.1747593 | Triglyceride levels in LDL→PCOS | PCOS as outcome | Triglyceride levels in LDL | Metabolites | OR | OR 1.17 (95% CI ) | 1.17 |  |  | IVW | 0.0480 | 0.048 | Karjalainen et al. metabolite GWAS | Not reported | Not reported | EBI database (ID: ebi-a-GCST90044902) | Not reported | Not reported | MR-Egger intercept; Cochran Q; leave-one-out; weighted median; scatter plot; funnel plot; forest plot |
|  | Xu | 2026 | Front. Med. | 10.3389/fmed.2026.1747593 | Ratio of apolipoprotein B to apolipoprotein A1→PCOS | PCOS as outcome | Ratio of apolipoprotein B to apolipoprotein A1 | Physiological traits | OR | OR 1.15 (95% CI ) | 1.15 |  |  | IVW | 0.0400 | 0.04 | Karjalainen et al. metabolite GWAS | Not reported | Not reported | EBI database (ID: ebi-a-GCST90044902) | Not reported | Not reported | MR-Egger intercept; Cochran Q; leave-one-out; weighted median; scatter plot; funnel plot; forest plot |
|  | Yan | 2026 | International Journal of Women’s Health | 10.2147/IJWH.S605528 | Cystatin-F→PCOS | PCOS as outcome | Cystatin-F | Proteins or genes | OR | OR 0.85 (0.73–1.00) | 0.85 | 0.73 | -1 | IVW | 0.0490 | 0.049 | MRC-IEU OpenGWAS database; Cystatin-F GWAS (prot-a-705) | European | 3301 | FinnGen consortium; PCOS GWAS (finn-b-E4_POCS) | European | cases/controls = 642/118228 | MR-Egger intercept; Cochran Q; leave-one-out; weighted median; weighted mode; simple mode; funnel plot; scatter plot; forest plot |
|  | Yan | 2026 | International Journal of Women’s Health | 10.2147/IJWH.S605528 | PCOS→Cystatin-F | PCOS as exposure | Cystatin-F | Proteins or genes | Unclear | ( GWAS SNP , F-statistic, ) |  |  |  | IVW | Not reported |  | FinnGen consortium; PCOS GWAS (finn-b-E4_POCS) | European | cases/controls = 642/118228 | MRC-IEU OpenGWAS database; Cystatin-F GWAS (prot-a-705) | European | 3301 | Not reported |
|  | Yan | 2026 | International Journal of Women’s Health | 10.2147/IJWH.S605528 | PCOS→Cystatin-F | PCOS as exposure | Cystatin-F | Proteins or genes | Unclear | (SNPN) |  |  |  | IVW | Not reported |  | FinnGen; finn-b-E4_POCS | European | cases/controls = 642/118228 | MRC-IEU OpenGWAS database; prot-a-705 | European | 3301 | Not reported |
|  | Yang | 2026 | Medicine | 10.1097/MD.0000000000049143 | PCOS→fetal/neonatal hemorrhagic and hematologic disorders | PCOS as exposure | fetal/neonatal hemorrhagic and hematologic disorders | Diseases or clinical phenotypes | OR | OR 1.80 (1.11–2.91) | 1.8 | 1.11 | -2.91 | IVW | 0.0167 | 0.0167 | ebi-a-GCST90044902 GWAS | Not reported | Not reported | FinnGen consortium (finn-bP16_HAEMORRHAGIC_HAEMATOL_DISORD_FETUS_NEWBO) | Finnish | Not reported | MR-Egger intercept; Cochran Q; Steiger test; leave-one-out; funnel plot; weighted median |
|  | Yang | 2026 | Medicine | 10.1097/MD.0000000000049143 | PCOS→fetal/neonatal hemorrhagic and hematologic disorders | PCOS as exposure | hemorrhagic and haematological disorders of the fetus or newborn | Diseases or clinical phenotypes | OR | OR 1.80 (1.11–2.91) | 1.8 | 1.11 | -2.91 | IVW | 0.0167 | 0.0167 | ebi-a-GCST90044902 GWAS | Not reported | Not reported | FinnGen consortium (finn-bP16_HAEMORRHAGIC_HAEMATOL_DISORD_FETUS_NEWBO) | Finnish | Not reported | MR-Egger intercept; Cochran Q; Steiger test; leave-one-out; funnel plot; weighted median; MR-Egger; simple mode; weighted mode |
|  | Yang | 2026 | Brain Imaging and Behavior | 10.1007/s11682-026-01143-9 | PCOS (consortium definition)→cortical surficial area of caudal middle frontal gyrus | PCOS as exposure | cortical surficial area of caudal middle frontal gyrus | Physiological traits | Beta | β -20.74 (-39.05–-2.42) | -20.74 | -39.05 | -2.42 | IVW | 0.0265 | 0.0265 | FinnGen | European | cases/controls = 31548/179322 | ENIGMA Consortium | Not reported | 51665 | MR-Egger intercept; MR-PRESSO global test; Cochran Q; leave-one-out; funnel plot; weighted median; weighted mode; MR-Egger |
|  | Yang | 2026 | Brain Imaging and Behavior | 10.1007/s11682-026-01143-9 | PCOS→cortical surficial area of caudal middle frontal gyrus | PCOS as exposure | cortical surficial area of caudal middle frontal gyrus | Physiological traits | Beta | β -20.74 (-39.05–-2.42) | -20.74 | -39.05 | -2.42 | IVW | 0.0265 | 0.0265 | FinnGen PCOS consortium definition phenotype | European | cases/controls = 31548/179322 | ENIGMA Consortium | Not reported | 51665 | MR-Egger intercept; MR-PRESSO global test; Cochran Q; leave-one-out; funnel plot; weighted median; weighted mode; MR-Egger |
|  | Yang | 2026 | Brain Imaging and Behavior | 10.1007/s11682-026-01143-9 | PCOS→cortical surficial area of inferior parietal lobule | PCOS as exposure | cortical surficial area of inferior parietal lobule | Physiological traits | Beta | β -6.13 (-11.57–-0.69) | -6.13 | -11.57 | -0.69 | IVW | 0.0273 | 0.0273 | Tyrmi JS (2022) and FinnGen PCOS GWAS | Not reported | Not reported | ENIGMA Consortium | Not reported | 51665 | MR-Egger intercept; MR-PRESSO global test; Cochran Q; leave-one-out; funnel plot; weighted median; weighted mode; MR-Egger |
|  | Yang | 2026 | Brain Imaging and Behavior | 10.1007/s11682-026-01143-9 | PCOS (adjusted for age)→cortical surficial area of pars opercularis | PCOS as exposure | cortical surficial area of pars opercularis | Physiological traits | Beta | β 5.70 (0.02–11.37) | 5.7 | 0.02 | -11.37 | IVW | 0.0491 | 0.0491 | Tyrmi JS (2022) and FinnGen PCOS GWAS | European | Not reported | ENIGMA Consortium | Not reported | 51665 | MR-Egger intercept; MR-PRESSO global test; Cochran Q; leave-one-out; funnel plot; weighted median; weighted mode; MR-Egger |
|  | Yang | 2026 | Brain Imaging and Behavior | 10.1007/s11682-026-01143-9 | PCOS→cortical surficial area of pars opercularis | PCOS as exposure | cortical surficial area of pars opercularis | Physiological traits | Beta | β 5.70 (0.02–11.37) | 5.7 | 0.02 | -11.37 | IVW | 0.0491 | 0.0491 | Tyrmi JS (2022) / FinnGen PCOS phenotype (adjusted for age) | Not reported | Not reported | ENIGMA Consortium | Not reported | 51665 | MR-Egger intercept; MR-PRESSO global test; Cochran Q; leave-one-out; funnel plot; weighted median; weighted mode; MR-Egger |
