# Supplementary material for: Mapping the causal landscape of polycystic ovary syndrome: A systematic Mendelian randomization evidence synthesis and the MR-PCOS atlas platform
Source: Medicine (Baltimore). 2026 Aug 21;105(34):e50366. doi: 10.1097/MD.0000000000050366 (PMC13502786; doi:10.1097/MD.0000000000050366)
Supplement: Supplementary file 4 [file medi-105-e50366-s004.docx]

| First author | Year | Journal | DOI | Instrument selection threshold | LD clumping parameters | Weak instrument assessment | Horizontal pleiotropy assessment | Heterogeneity assessment | Directionality or robustness analysis | Multivariable or mediation adjustment | Multiple-testing correction | Overall methodological quality |
| --- | --- | --- | --- | --- | --- | --- | --- | --- | --- | --- | --- | --- |
|  |  |  |  |  |  |  |  |  |  |  |  |  |
| Li | 2026 | Journal of Ovarian Research | 10.1186/s13048-026-01970-w | P < 5×10^-8 | r² < 0.001, window = 10000 kb | F-statistic | MR-Egger intercept; MR-PRESSO | Cochran Q | weighted median; maximum likelihood; MR-Lap | 不存在 | FDR | 中 |
| Yang | 2026 | Medicine | 10.1097/MD.0000000000049143 | P < 5×10^-6 | r² < 0.001, window = 10000 kb | F-statistic | MR-Egger intercept; funnel plot | Cochran Q; funnel plot | Steiger test; leave-one-out | 不存在 | 不存在 | 中 |
| Chen | 2026 | Journal of Obstetrics and Gynaecology Research | 10.1111/jog.70233 | P < 5×10^-8；sleep apnea syndrome 使用放宽阈值 P < 5×10^-6 | r² < 0.001, window > 10000 kb | F-statistic | MR-Egger intercept; MR-PRESSO; MR-PRESSO global test | Cochran Q | leave-one-out; weighted median; bidirectional analysis; MVMR-Egger; MVMR-median | Obesity (BMI, WHR, WC, HC) and insulin resistance represented by fasting insulin | 未明确报告 | 中 |
| Niu | 2025 | Gynecologic and Obstetric Investigation | 10.1159/000546487 | P < 5×10^-8 | r² < 0.001, window = 10000 kb | F-statistic | MR-Egger intercept | Cochran Q | leave-one-out | 不存在 | 不存在 | 中 |
| Jiang | 2026 | Ecotoxicology and Environmental Safety | 10.1016/j.ecoenv.2026.119900 | P < 1e-08 | r² < 0.1, window = 1000 kb | F-statistic | MR-Egger intercept | Cochran Q |  | 不存在 | Bonferroni | 中 |
| Zhang | 2024 | Combinatorial Chemistry & High Throughput Screening | 10.2174/0113862073325251241101054306 | P < 5×10^-8 | r² ≤ 0.001, window = 10000 kb | F-statistic/R² | MR-Egger intercept; funnel plot | Cochran Q; funnel plot | leave-one-out | 不存在 | 不存在 | 中 |
| Zheng | 2026 | International Journal of Gynecology and Obstetrics | 10.1002/ijgo.70411 | P < 5×10^-8 | r² < 0.01, window = 10000 kb | F-statistic | MR-Egger intercept; MR-PRESSO | Cochran Q; funnel plot | leave-one-out; replication analysis | 不存在 | 不存在 | 中 |
| Wen | 2025 | The World Journal of Biological Psychiatry | 10.1080/15622975.2025.2555173 | P < 5×10^-8 | r² < 0.001, window = 10000 kb | F-statistic/R² | MR-Egger intercept; MR-PRESSO | Cochran Q; funnel plot | leave-one-out | 不存在 | 未明确报告 | 中 |
| Zhang | 2026 | International Journal of Women's Health | 10.2147/IJWH.S571454 | P < 5.0 × 10^-8; if SNPs fewer than three, relaxed to P < 5.0 × 10^-7 | r² > 0.001 within a 10,000 kb window | 不存在 | MR-Egger intercept; MR-PRESSO | Cochran Q | leave-one-out | 不存在 | 不确定 | 中 |
| Fu | 2026 | International Journal of Women's Health | 10.2147/IJWH.S576568 | P < 5×10^-6 | r² < 0.001, window = 1000 kb | F-statistic | MR-Egger intercept; MR-PRESSO; PhenoScanner | Cochran Q; funnel plot | leave-one-out; replication analysis; weighted median | 不存在 | FDR | 高 |
| Wang | 2026 | Ecotoxicology and Environmental Safety | 10.1016/j.ecoenv.2026.119742 | exposure instruments: P < 5×10^-6; reverse MR: P < 5×10^-8 | r² < 0.001, window = 10000 kb | F-statistic | MR-Egger intercept | Cochran Q | BWMR | 不存在 | FDR | 中 |
| Xu | 2026 | Front. Med. | 10.3389/fmed.2026.1747593 | Methods部分写为 P < 5×10^-8；Results部分又写为 p < 1×10^-5，存在不一致 | r² < 0.001, window = 10000 kb | F-statistic | MR-Egger intercept | Cochran Q; funnel plot | leave-one-out | 不存在 | 不存在 | 中 |
| Luo | 2026 | International Journal of Gynecology and Obstetrics | 10.1002/ijgo.70897 | P < 5×10^-8 | r² < 0.01, window = 10 Mb | F-statistic | MR-Egger intercept; MR-PRESSO | Cochran Q; funnel plot | leave-one-out | 不存在 | Bonferroni | 中 |
| You | 2026 | International Journal of Women's Health | 10.2147/IJWH.S586045 | P < 5×10^-8 for PCOS, diabetes, BMI, and HRT; P < 5×10^-7 for ever use of OCs | r² < 0.001, window = 10000 kb | F-statistic | MR-Egger intercept; MR-PRESSO; MR-PRESSO global test | Cochran Q; funnel plot | leave-one-out | diabetes, BMI, hormone replacement therapy use, oral contraceptive use | 不存在 | 中 |
| Guo | 2026 | International Journal of Women's Health | 10.2147/IJWH.S583951 | P < 5×10^-8 | r² < 0.001, window = 10000 kb | F-statistic | MR-Egger intercept | Cochran Q; funnel plot | Steiger test; leave-one-out | 不存在 | 未明确报告 | 中 |
| Xu | 2026 | International Journal of Women's Health | 10.2147/IJWH.S586268 | P < 5×10^-8; relaxed to P < 5×10^-6 for traits with limited SNPs such as sugar added to coffee or tea, bran cereal, and oat cereal | r² < 0.001, window = 10000 kb | F-statistic | MR-Egger intercept; MR-PRESSO | Cochran Q; funnel plot | leave-one-out | 不存在 | 未明确报告 | 中 |
| Wang | 2026 | International Journal of Endocrinology | 10.1155/ije/3604933 | P < 5×10^-8; if no genome-wide significant SNPs, P < 5×10^-6 | r² < 0.001, window = 10000 kb | F-statistic | MR-Egger intercept; MR-PRESSO | Cochran Q | leave-one-out; weighted median; MR-Egger; scatter plot | 中介变量：BMI；使用 two-step MR 评估 OSA→BMI→PCOS | 不存在 | 中 |
| Lu | 2026 | International Journal of Women's Health | 10.2147/IJWH.S593314 | P < 5×10^-8 | r² < 0.001, window = 10 kb | F-statistic | MR-Egger intercept; MR-PRESSO global test | Cochran Q; funnel plot | Steiger test; leave-one-out | 不存在 | 不存在 | 中 |
| Yan | 2026 | International Journal of Women’s Health | 10.2147/IJWH.S605528 | P < 5×10^-6 | r² < 0.001, window = 10000 kb | F-statistic/R² | MR-Egger intercept; funnel plot | Cochran Q; funnel plot | leave-one-out; bidirectional MR | 不存在 | 未明确报告 | 中 |
| Wang | 2026 | Medicine | 10.1097/MD.0000000000048704 | P < 5×10^-8 | r² < 0.01 | F-statistic | MR-Egger intercept | Cochran Q; funnel plot | leave-one-out; replication analysis | 不存在 | 不存在 | 中 |
| He | 2026 | International Journal of Biological Sciences | 10.7150/ijbs.116803 | P < 5.00E-8 or 5.00E-6 | r² < 0.001, window = 10000 kb | 不存在 | MR-Egger intercept; MR-PRESSO | Cochran Q; I²; funnel plot | leave-one-out | MVMR adjusted for major determinants of exposure and outcome to minimize bias；具体调整变量未明确；中介变量为serum leucine levels | Bonferroni | 中 |
| Xie | 2025 | International Journal of Women's Health | 不确定 | PCOS: P < 5×10^-8; plasma lipid groups and immune cell traits: P < 1×10^-5 | r² < 0.001, window = 10000 kb | F-statistic | MR-Egger intercept; MR-PRESSO | Cochran Q | leave-one-out; BWMR | 中介变量：immune cell traits, especially SSC-A on CD4+ | Bonferroni | 中 |
| Fu | 2026 | BMC Women's Health | 10.1186/s12905-025-04237-6 | P < 5×10^-8；部分暴露在SNP不足时放宽至 P < 5×10^-6 | r² < 0.01, window = 5,000 kb | F-statistic；R² | MR-Egger intercept; MR-PRESSO | Cochran Q; funnel plot | leave-one-out | 不存在 | FDR | 中 |
| Lai | 2025 | Discover Oncology | 10.1007/s12672-025-01861-4 | P < 5×10^-8 | r² < 0.001, window = 不确定 | F-statistic | MR-Egger intercept | Cochran Q; funnel plot | leave-one-out; weighted median | 不存在 | Benjamini–Hochberg false discovery rate | 中 |
| Liu | 2025 | Journal of Ovarian Research | 10.1186/s13048-025-01617-2 | P < 5×10^-6 | r² < 0.001, window = 10000 kb | 不存在 | MR-Egger intercept; MR-PRESSO | Cochran Q; funnel plot | Steiger test; leave-one-out; scatter plot | 不存在 | 不存在 | 中 |
| Dang | 2025 | Diabetology & Metabolic Syndrome | 10.1186/s13098-025-01739-z | P < 5×10^-8 | r² < 0.001, window = 10000 kb | F-statistic | MR-Egger intercept; MR-PRESSO | Cochran Q; funnel plot | Steiger test; leave-one-out; BWMR | 不存在 | 未明确报告 | 中 |
| Arruda | 2025 | Nature Communications | 10.1038/s41467-025-64927-5 | P < 5×10^-8 | T2D main IVs: index variants with r2 < 0.05 over a 5 Mb window; other traits: r2 < 0.001, window = 10 Mb | F-statistic | MR-Egger intercept; MR-PRESSO | I²; Cochran Q | Steiger test; weighted median; MR-Egger; MR-PRESSO; comparison with alternative IV selection approaches; leave-one-cluster-out; correlated IVW | 存在；对部分cluster-stratified结果使用cardiometabolic traits进行MVMR调整，但原文未报告针对PCOS的具体MVMR结果 | FDR | 中 |
| Ye | 2025 | Nature Communications | 10.1038/s41467-025-64985-9 | UVMR中 IR phenotypes IVs 为 P < 5×10^-8；HOMA-IR 例外为 P < 1×10^-5；druggable genome MR中排除 F-statistic≤10 的SNP | UVMR/MVMR: r² < 0.001, window = 10000 kb; druggable genome MR: r² < 0.1 | F-statistic | MR-Egger intercept | Cochran Q | weighted median; simple mode; weighted mode; MR-Egger; MVMR-median; multivariable MR-Egger; multivariable MR-Lasso; Steiger test | MVMR adjusted for muscle or adiposity traits including GS, ALM, WBLM, BF%, BMI, and WHR | FDR | 中 |
| Yu | 2025 | Signal Transduction and Targeted Therapy | 10.1038/s41392-025-02468-5 | P < 5×10^-8 | 不确定 | F-statistic | MR-Egger intercept; MR-PRESSO | Cochran Q; funnel plot | leave-one-out; weighted median; weighted mode; MR-Egger | Multivariable MR performed among glycolysis-related factors / key metabolic enzymes; specific adjusted variables not fully reported in provided full text | 不存在 | 中 |
| Wei | 2025 | Eating and Weight Disorders - Studies on Anorexia, Bulimia and Obesity | 10.1007/s40519-025-01801-9 | P < 5×10^-6 | r² < 0.0001, window = 10000 kb | F-statistic | MR-Egger intercept | Cochran Q; funnel plot | leave-one-out | 不存在 | FDR | 中 |
| Zhang | 2025 | Medicine | 10.1097/MD.0000000000045566 | P < 5×10^-6 | r² < 0.001, window = 10000 kb | F-statistic/R² | MR-Egger intercept; MR-PRESSO | Cochran Q | leave-one-out | 不存在 | Bonferroni | 高 |
| Xing | 2025 | Gynecology and Obstetrics Clinical Medicine | 10.1136/gocm-2025-000268 | P < 5×10^-8; to maximise variance explained, threshold of 1×10^-5 also described | r² < 0.001, window = 10000 kb | F-statistic | MR-Egger intercept; MR-PRESSO | Cochran Q | leave-one-out | 不存在 | FDR | 中 |
| Chen | 2025 | Medicine | 10.1097/MD.0000000000044448 | P < 5×10^-6 prioritized; P < 1×10^-5 considered when unavailable | r² < 0.001, window = 10000 kb | F-statistic | MR-Egger intercept; MR-PRESSO; PhenoScanner | Cochran Q; funnel plot | Steiger test; leave-one-out; MR-RAPS | dopamine 4-sulfate to dopamine 3-O-sulfate ratio as mediator in two-step MR | 不存在 | 中 |
| Yu | 2025 | Health Science Reports | 10.1002/hsr2.71285 | P < 5×10^-8 | r² < 0.001, window = 10000 kb | F-statistic | MR-Egger intercept; MR-PRESSO | Cochran Q; MR-PRESSO global test | weighted median; simple mode; weighted mode; SMR; HEIDI; colocalization analysis | 不存在 | FDR | 中 |
| Ge | 2025 | Journal of Clinical Endocrinology & Metabolism | 10.1210/clinem/dgaf057 | 不确定 | 不确定 | 不存在 |  |  |  | 不存在 | 不存在 | 低 |
| Aru | 2025 | Medicine | 10.1097/MD.0000000000042685 | significant associations with LDL-C levels; exact P threshold not clearly reported for instrument selection | r² < 0.3, within 100 kb upstream or downstream of PCSK9 and HMGCR gene regions | F-statistic | MR-Egger intercept; MR-PRESSO; GWAS Catalog screening for pleiotropic SNPs | Cochran Q | leave-one-out; replication analysis | 不存在 | 不存在 | 中 |
| Hong | 2025 | F S Sci | 10.1016/j.xfss.2025.02.003 | P < 5×10^-8 | r² < 0.001, window = 10000 kb | 不存在 | MR-Egger intercept; PheWAS | Cochran Q | leave-one-out; MR-Egger; weighted median; simple mode; weighted mode; colocalization analysis | 不存在 | Bonferroni | 中 |
| Gao | 2025 | Endocrine, Metabolic & Immune Disorders - Drug Targets | 10.2174/0118715303363459250317084005 | P < 5×10^-6（因满足P < 5×10^-8的SNP数量有限） | r² < 0.001, window = 10000 kb | F-statistic | MR-Egger intercept; MR-PRESSO | Cochran Q; funnel plot | leave-one-out | 不存在 | 不存在 | 中 |
| Zhang | 2025 | International Journal of Gynecology and Obstetrics | 10.1002/ijgo.16029 | Primary: P < 5×10^-8; if no SNPs available, relaxed to P < 5×10^-5 | r² = 0.001, window = 10000 kb | F-statistic | MR-Egger intercept; MR-PRESSO | Cochran Q | Steiger test; leave-one-out | 不存在 | FDR | 中 |
| Peng | 2025 | International Journal of Gynecology & Obstetrics | 10.1002/ijgo.16057 | P < 5×10^-8；POF及为获得足够SNP时使用 P < 5×10^-6 | r² < 0.001, window = 10000 kb | F-statistic | MR-Egger intercept | Cochran Q | leave-one-out; replication analysis | 不存在 | 不存在 | 中 |
| Motafeghi | 2025 | Int J Endocrinol Metab | 10.5812/ijem-159124 | P < 5×10^-8 | r² < 0.001, window = 10000 kb | F-statistic | MR-Egger intercept; MR-PRESSO; PhenoScanner | Cochran Q; I²; funnel plot; Rucker’s Q | leave-one-out; weighted median; weighted mode; simple mode; MR-RAPS; MR-lasso; Robust IVW; single SNP MR | 不存在 | 不存在 | 中 |
| Lin | 2025 | PLOS ONE | 10.1371/journal.pone.0321380 | P < 5×10^-8 | r² < 0.01；window = 不确定 | F-statistic | MR-Egger intercept | Cochran Q; funnel plot | leave-one-out | 不存在 | Bonferroni | 中 |
| Wang | 2025 | Advanced Science | 10.1002/advs.202411679 | P < 5×10^-8 for hemoglobin; cis-eQTLs P < 5×10^-4; cis-pQTLs P < 5×10^-8 | r² < 0.001, window = 10000 kb for main MR; eQTL r² < 0.1; pQTL pruning r² > 0.01 | F-statistic | MR-Egger intercept; MR-PRESSO |  | Steiger test; replication analysis; weighted median; MR-PRESSO; MR-Egger regression; type 1 error assessment for partial sample overlap | MVMR adjusted for bioavailable testosterone or total testosterone | Bonferroni/FDR | 中 |
| Chan | 2025 | Endocrinology, Diabetes & Metabolism | 10.1002/edm2.70030 | P < 5×10^-8 | r² < 0.001, window = 10000 kb | F-statistic/R² | MR-Egger intercept | Cochran Q | leave-one-out | 不存在 | 不存在 | 中 |
| Jiang | 2025 | Medicine | 10.1097/MD.0000000000041359 | Cytokines: initially P < 5×10^-8, relaxed to P < 5×10^-6; PCOS: P < 5×10^-8 | Cytokines: r² < 0.001, window = 250 kb; PCOS: r² < 0.001, window = 10000 kb | F-statistic | MR-Egger intercept; MR-PRESSO | Cochran Q | leave-one-out; replication analysis | 不存在 | FDR | 高 |
| Shao | 2025 | European Journal of Obstetrics & Gynecology and Reproductive Biology | 10.1016/j.ejogrb.2024.12.043 | P < 5×10^-8 | r² < 0.001 | F-statistic | MR-Egger intercept; MR-PRESSO | Cochran Q; funnel plot | leave-one-out; replication analysis | 不存在 | 未明确报告 | 中 |
| Ma | 2025 | Scientific Reports | 10.1038/s41598-025-86794-2 | P < 5×10^-6 | r² < 0.001, window = 10000 kb | F-statistic | MR-Egger intercept; MR-PRESSO | Cochran Q; funnel plot | Steiger test; leave-one-out; reverse MR analysis | BMI, obesity, diabetes, smoking, alcohol use, physical activity, inflammation, depression, waist-hip ratio, vitamin D, dehydroepiandrosterone sulphate, sex hormone binding globulin, testosterone; mediation MR also evaluated multiple potential mediators | FDR | 中 |
| Li | 2025 | Frontiers in Endocrinology | 10.3389/fendo.2025.1534733 | P < 5×10^-8 | r² < 0.001, window = 1000 kb | F-statistic | MR-Egger intercept; MR-PRESSO; phenoscanner | Cochran Q | leave-one-out | 不存在 | FDR | 中 |
| Fang | 2025 | International Journal of Women's Health | 不确定 | P < 5×10^-8 | r² < 0.01; window = 不确定 | F-statistic | MR-Egger intercept; MR-PRESSO | Cochran Q | Steiger test; leave-one-out | 中介变量为BMI | 不存在 | 中 |
| Xin | 2025 | International Journal of Women's Health | 不确定 | P < 5×10^-8; for OD and PCOS analyses in two-step MR, p < 5×10^-6 was used due to limited significant loci | r² < 0.001, window = 10000 kb | F-statistic/R² | MR-Egger intercept; MR-PRESSO | Cochran Q; funnel plot | leave-one-out | MVMR adjusted for BMI, depression, ischemic heart disease, diabetes; spontaneous abortion models additionally considered smoking, alcohol consumption, coffee intake, and insomnia; mediation variable was BMI | 不存在 | 中 |
| Tao | 2025 | International Journal of Women's Health | 不确定 | P < 1e−5 | r² < 0.1, window = 500 kb | F-statistic | MR-Egger intercept; MR-PRESSO | Cochran Q; funnel plot | leave-one-out; weighted median | 不存在 | FDR | 中 |
| Chen | 2025 | Journal of Multidisciplinary Healthcare | 不确定 | P < 1×10^-5 | r² < 0.1, window = 500 kb | F-statistic | MR-Egger intercept; MR-PRESSO | Cochran Q; funnel plot | Steiger test; leave-one-out | 不存在 | 不存在 | 中 |
| Wang | 2025 | International Journal of Women's Health | 不确定 | P < 5×10^-8 | r² < 0.001, window = 10000 kb | F-statistic | MR-Egger intercept; MR-PRESSO; MR-PRESSO global test | Cochran Q; funnel plot | leave-one-out | 不存在 | 不存在 | 中 |
| Fang | 2024 | Journal of Clinical Endocrinology and Metabolism | 10.1210/clinem/dgae446 | P < 5×10^-8；MAF in outcome > 0.01 | r² < 0.001, window = 10000 kb | F-statistic | MR-Egger intercept; MR-PRESSO | Cochran Q | leave-one-out | 不存在 | 不存在 | 中 |
| Fu | 2025 | Am J Transl Res | 10.62347/PGYY9493 | P < 5e-06 for most SNPs across four sports data categories | r² < 0.01, window = 5000 kb | F-statistic | MR-Egger; MR-PRESSO | funnel plot | leave-one-out | 不存在 | 不存在 | 中 |
| Kou | 2025 | International Journal of Women's Health | 不确定 | P < 5×10^-8; for 179 lipid traits, walking time, sedentary behavior, moderate-intensity physical activity, total physical activity time, and when PCOS was used as exposure, P < 5×10^-6 | r² < 0.001, window = 10000 kb | F-statistic | MR-Egger intercept; MR-PRESSO; MR-PRESSO global test | Cochran Q | Steiger test; leave-one-out; weighted median; MR-Egger; weighted mode; simple mode | two-step MR mediation with TAG (50:1) and other lipid traits as mediators; 无 multivariable MR | 不存在 | 中 |
| Qu | 2025 | International Journal of Women's Health | 不确定 | For inflammatory proteins P < 1×10^-5; for reverse MR with ovarian-related diseases as exposure P < 5×10^-8 | r² < 0.001, window = 10000 kb | F-statistic | MR-Egger intercept; MR-PRESSO | Cochran Q | Steiger test; leave-one-out | 不存在 | 未明确报告 | 中 |
| Ye | 2025 | International Journal of Women's Health | 不确定 | P < 1×10^-5（原文说明因 P < 5×10^-8 时 SNP 数量不足而采用放宽阈值） | r² = 0.001, window = 10000 kb | F-statistic | MR-PRESSO | 不确定 | leave-one-out | 不存在 | 不存在 | 中 |
| Ni | 2024 | The American Journal of Human Genetics | 10.1016/j.ajhg.2024.10.008 | P < 5×10^-8 | r² < 0.1, window = 5000 kb | F-statistic/R² | MR-Egger intercept; MR-PRESSO | HEIDI test | weighted median; simple mode; weighted mode; replication analysis; colocalization analysis; SMR | 不存在 | FDR | 中 |
| Gao | 2024 | Journal of Ovarian Research | 10.1186/s13048-024-01420-5 | P < 5×10^-8 | r² < 0.001, window = 10000 kb | F-statistic/R² | MR-Egger intercept; MR-PRESSO | Cochran Q; funnel plot | leave-one-out | BMI, FBG, FI, BT | 不存在 | 中 |
| Guo | 2024 | BMC Women's Health | 10.1186/s12905-024-03144-6 | P < 5×10^-8 | r² < 0.001, window = 10000 kb | F-statistic | MR-Egger intercept; MR-PRESSO | Cochran Q | replication analysis | 不存在 | Bonferroni | 中 |
| Zeng | 2024 | Diabetology & Metabolic Syndrome | 10.1186/s13098-024-01404-x | P < 5×10^-8 | r² = 0.001, window = 10000 kb | F-statistic | MR-Egger intercept; MR-PRESSO | Cochran Q | weighted median | MVMR included three glycemic traits; mediation variable was type II diabetes in two-step MR | 不存在 | 中 |
| Yang | 2024 | Journal of Ovarian Research | 10.1186/s13048-024-01466-5 | P < 5×10^-8 | r² < 0.001, window = 10000 kb | F-statistic/R² | MR-Egger intercept; MR-PRESSO | Cochran Q; funnel plot | leave-one-out; weighted median; weighted mode; simple mode | current tobacco smoking, alcohol consumption, BMI, sleeplessness/insomnia, history of PID | Bonferroni | 中 |
| Chen | 2024 | Journal of Ovarian Research | 10.1186/s13048-024-01486-1 | P < 1×10^-5 | r² < 0.1, window = 500 kb | F-statistic | MR-Egger intercept; MR-PRESSO global test | Cochran Q | Steiger test; replication analysis; Radial MR | 不存在 | 不存在 | 中 |
| Kong | 2024 | BMC Women's Health | 10.1186/s12905-024-03347-x | P < 5×10^-8; when fewer than three SNPs, relaxed to P < 5×10^-6 | r² < 0.001, window = 10000 kb | F-statistic | MR-Egger intercept | Cochran Q | leave-one-out | 不存在 | 不存在 | 中 |
| Tian | 2024 | Journal of Ovarian Research | 10.1186/s13048-024-01525-x | P < 1×10^-5 | r² < 0.001, window = 10000 kb | F-statistic | MR-Egger intercept; MR-PRESSO | Cochran Q | weighted median; weighted mode; simple mode | 不存在 | 不存在 | 中 |
| Jiang | 2024 | Multiple Sclerosis Journal | 10.1177/13524585241292802 | 不确定 | 不确定 | R² | MR-Egger intercept; MR-PRESSO | Cochran Q | leave-one-out; replication analysis | 不存在 | 不确定 | 中 |
| Bi | 2024 | Breast Cancer Research | 10.1186/s13058-024-01923-5 | 不确定 | 不确定 | 不存在 | 不存在 | 不存在 | 不存在 | 不存在 | Bonferroni | 低 |
| Alesi | 2024 | Biomolecules | 10.3390/biom14121544 | P < 5×10^-6 | r² < 0.01, window = 10000 kb | F-statistic | MR-Egger intercept | Cochran Q; funnel plot | leave-one-out | 不存在 | Bonferroni | 中 |
| Zhang | 2024 | Human Reproduction | 10.1093/humrep/deae244 | P < 5×10^-8 | r² < 0.001, window = 不确定 | F-statistic | MR-Egger intercept; MR-PRESSO | Cochran Q; I² | leave-one-out | 不存在 | FDR | 中 |
| Liu | 2024 | Journal of Thoracic Disease | 10.21037/jtd-24-885 | P < 5×10^-8 | r² < 0.001, window = 10000 kb | F-statistic | MR-Egger intercept | Cochran Q; funnel plot | leave-one-out | 不存在 | 不存在 | 中 |
| Wang | 2024 | Br J Hosp Med | 10.12968/hmed.2024.0478 | P < 5×10^-6 | r² < 0.001, window = 10000 kb | 不存在 | MR-Egger intercept; MR-PRESSO | Cochran Q | leave-one-out | 不存在 | 不存在 | 中 |
| Gao | 2024 | Medicine | 10.1097/MD.0000000000040115 | Gut microbiota: P < 1 × 10^-5; risk factors: P < 5 × 10^-8 | r² < 0.001, window > 10000 kb | 不存在 | MR-Egger intercept; MR-PRESSO |  | weighted median; MR-Maxlik; Radial MR | 中介变量包括 BMI 和 waist circumference；使用network MR评估 Sellimonas→risk factor→PCOS | Bonferroni | 中 |
| Lu | 2024 | International Journal of Surgery | 10.1097/JS9.0000000000001814 | P < 1×10^-5 | r² < 0.001, window = 10000 kb | F-statistic | MR-Egger intercept; MR-PRESSO | Cochran Q; I² | leave-one-out | 不存在 | Bonferroni | 中 |
| Shen | 2024 | Reproductive Sciences | 10.1007/s43032-024-01476-0 | P < 5×10^-8 | r² < 0.01, window = 1000 kb | 不存在 | MR-Egger intercept; MR-PRESSO | Cochran Q | leave-one-out | 不存在 | 不存在 | 中 |
| Wang | 2024 | Fertility and Sterility | 10.1016/j.fertnstert.2024.02.011 | P < 5×10^-8 | R2 < 0.001 | F-statistic | MR-Egger intercept | Cochran Q; I² | leave-one-out | 不存在 | 未明确报告 | 中 |
| Ling | 2024 | Scientific Reports | 10.1038/s41598-024-55993-8 | P < 5×10^-8; reverse MR for traits with limited SNPs used relaxed threshold P < 5×10^-6 | r² < 0.001, window = 10000 kb | F-statistic/R² | MR-Egger intercept; MR-PRESSO; funnel plot | Cochran Q; funnel plot | leave-one-out | 不存在 | Bonferroni | 中 |
| Guixue | 2024 | Frontiers in Endocrinology | 10.3389/fendo.2024.1337562 | P < 5×10^-8 | r² < 0.001, window = 10000 kb | F-statistic | MR-Egger intercept | Cochran Q; funnel plot | leave-one-out | 不存在 | 不存在 | 中 |
| Zhao | 2024 | Frontiers in Endocrinology | 10.3389/fendo.2024.1364157 | forward MR: P < 5×10^-8; reverse MR: P < 5×10^-6 | r² < 0.001, window = 10000 kb | F-statistic | MR-Egger intercept; MR-PRESSO; funnel plot | Cochran Q; funnel plot | leave-one-out | 不存在 | Bonferroni | 中 |
| Tan | 2024 | Frontiers in Endocrinology | 10.3389/fendo.2024.1359236 | P ≤ 5×10^-8；若存在异质性则可使用 1×10^-6 | r² < 0.001, window = 1000 kb | F-statistic | MR-Egger; MR-PRESSO | Cochran Q | weighted median; simple mode; weighted mode | 不存在 | 不存在 | 中 |
| Su | 2024 | Frontiers in Endocrinology | 10.3389/fendo.2024.1393847 | P < 5×10^-8; MAF ≥ 0.01 | r² < 0.001, window = 10000 kb | F-statistic/R² | MR-Egger intercept; MR-PRESSO | Cochran Q; funnel plot | leave-one-out | 不存在 | 不存在 | 中 |
| Liu | 2024 | Frontiers in Medicine | 10.3389/fmed.2024.1429783 | OSAS as exposure: P < 5×10^-8; PCOS as exposure: P < 5×10^-6 | r² < 0.001, window = 10000 kb | F-statistic | MR-Egger intercept; MR-PRESSO | Cochran Q | leave-one-out | 不存在 | 不存在 | 中 |
| Wang | 2024 | Frontiers in Endocrinology | 10.3389/fendo.2024.1391826 | P < 1×10^-5 | r² < 0.01, window = 500 kb | F-statistic | MR-Egger intercept; MR-PRESSO | Cochran Q; funnel plot | Steiger test; leave-one-out; replication analysis | 不存在 | 不存在 | 中 |
| Guo | 2024 | Gynecological Endocrinology | 10.1080/09513590.2024.2420963 | P < 1×10^-5 | r² < 0.001, window = 10000 kb | F-statistic | MR-Egger intercept; MR-PRESSO | Cochran Q; funnel plot | leave-one-out; bidirectional MR | 中介变量为 1-stearoyl-2-oleoyl-GPI (18:0/18:1) levels | 不存在 | 中 |
| Lin | 2024 | International Journal of Endocrinology | 10.1155/2024/3090797 | P < 5×10^-8 | r² < 0.05; proxy threshold r² > 0.8 for replacement search | F-statistic | MR-Egger intercept; MR-PRESSO | leave-one-out | Steiger test; leave-one-out; weighted median | 不存在 | 不存在 | 中 |
| Wan | 2024 | Gynecological Endocrinology | 10.1080/09513590.2024.2444380 | Forward MR: P < 5×10^-8; reverse MR: text reports threshold adjusted from 5×10^-8 to 1×10^-5, but discussion mentions 1×10^-6 | r² < 0.001, window = 10000 kb | F-statistic | MR-Egger intercept; MR-PRESSO | Cochran Q; funnel plot | leave-one-out; replication analysis | 不存在 | Bonferroni | 中 |
| Cao | 2024 | J. Bio-X Res. | 10.34133/jbioxresearch.0024 | European LTL GWAS: P < 8.31×10^-9; East Asian LTL GWAS: P < 5×10^-8 | European LTL instruments: r² > 0.01 used for clumping removal; East Asian 16 SNPs reported not in LD | F-statistic | MR-Egger intercept; funnel plot | Cochran Q; Rücker’s Q | leave-one-out; replication analysis | 不存在 | 不存在 | 中 |
| Liu | 2023 | BMC Medicine | 10.1186/s12916-023-02775-0 | P < 5×10^-8 | r² < 0.001, window = 10 Mb | F-statistic | MR-Egger intercept; MR-PRESSO | Cochran Q | leave-one-out; replication analysis; weighted median; MR-Egger; MR-PRESSO; IVW analysis after excluding obesity-related variants | MVMR and stepwise mediation analyses involving NAFLD, fasting insulin, fasting glucose, SHBG, and bioavailable testosterone; obesity-related variants were excluded in mediation analyses | Bonferroni | 中 |
| Pu | 2023 | BMC Medical Genomics | 10.1186/s12920-023-01581-0 | 主分析：P < 5×10^-8；反向OS→PCOS分析：P < 1×10^-5 | 主分析：r² < 0.001, window > 10000 kb；反向OS→PCOS分析：r² < 0.01, distance > 250 kb | F-statistic | MR-Egger intercept | Cochran Q; funnel plot | leave-one-out | 不存在 | 不存在 | 中 |
| Chen | 2023 | Human Genomics | 10.1186/s40246-023-00550-z | P < 5×10^-8 | r² < 0.001, window = 10 mb | F-statistic | MR-Egger intercept; MR-PRESSO | Cochran Q; funnel plot | leave-one-out; replication analysis | 不存在 | 未明确报告 | 高 |
| Ma | 2023 | International Journal of Gynecology and Obstetrics | 10.1002/ijgo.14820 | P < 5×10^-8 | r² ≤ 0.01 | F-statistic | MR-Egger intercept; MR-PRESSO | Cochran Q; funnel plot | leave-one-out | 不存在 | 不存在 | 中 |
| Shen | 2023 | World Journal of Clinical Cases | 10.12998/wjcc.v11.i23.5468 | P < 5×10^-8 for vitamins D, E, A, and B12; P < 5×10^-6 for vitamin K | r² < 0.01, window = 1000 kb | 不存在 | MR-Egger intercept; MR-PRESSO | Cochran Q | weighted median; MR-RAPS; MR-PRESSO | 不存在 | 不存在 | 中 |
| Dobbie | 2023 | Human Reproduction | 10.1093/humrep/dead053 | P < 5×10^-8 | r² < 0.001, window = 不确定 | F-statistic/R² | MR-Egger | 不存在 | weighted median; weighted mode; MR-Egger | adult body size | 不存在 | 中 |
| Xiao | 2023 | Journal of the American Heart Association | 10.1161/JAHA.121.026561 | 2-sample MR used genome-wide significant SNPs with P < 5×10^-8; 1-sample MR used cardiometabolic PGSs as instruments | r² = 0.1, window = 250 kb | F-statistic | MR-Egger intercept; MR-Egger | 不存在 | weighted median; bidirectional MR | 不存在 | FDR | 中 |
| Mu | 2023 | eBioMedicine | 10.1016/j.ebiom.2023.104492 | P < 5×10^-8 | 不确定 | F-statistic/R² | MR-Egger intercept; MR-PRESSO | Cochran Q; funnel plot | Steiger test; leave-one-out | Multivariable MR adjusted for the other BCAAs; mediation analysis不存在 | FDR | 中 |
| Zhang | 2023 | Frontiers in Endocrinology | 10.3389/fendo.2023.1110341 | P < 5×10^-8 | r² < 0.001, window = 10000 kb | F-statistic/R² | MR-Egger intercept; MR-PRESSO | Cochran Q | leave-one-out | BMI, WHR, WC, HC, fasting insulin | 不存在 | 中 |
| Du | 2023 | Frontiers in Endocrinology | 10.3389/fendo.2023.1120119 | P < 5×10^-8 | r² < 0.001 | F-statistic | MR-Egger intercept | Cochran Q | weighted median | 不存在 | 未明确报告 | 中 |
| Liu | 2023 | Frontiers in Endocrinology | 10.3389/fendo.2023.1140499 | P < 5×10^-8 | r² < 0.001, window = 10000 kb | F-statistic | MR-Egger intercept; MR-PRESSO; MR-PRESSO global test | Cochran Q | leave-one-out; replication analysis; weighted median; weighted mode; MR-Egger; MR-PRESSO | 不存在 | 不存在 | 中 |
| Liang | 2023 | Frontiers in Endocrinology | 10.3389/fendo.2023.1164186 | P < 1.0×10^-5 and P < 5×10^-8 | r² < 0.001, window = 10000 kb | F-statistic/R² | MR-Egger intercept; MR-PRESSO | Cochran Q | leave-one-out | 不存在 | FDR | 中 |
| Si | 2023 | Frontiers in Endocrinology | 10.3389/fendo.2023.1229900 | P < 5×10^-8 | r² < 0.01, window = 10 Mb | F-statistic/R² | MR-Egger intercept; MR-PRESSO | Cochran Q; funnel plot | leave-one-out | 不存在 | 不存在 | 中 |
| Zhao | 2023 | Frontiers in Endocrinology | 10.3389/fendo.2023.1295412 | LDL-C关联SNP达到genome-wide significance；MAF > 1% | r² < 0.3, window = 100 kb | 不存在 | MR-Egger intercept; MR-PRESSO | Cochran Q | leave-one-out | 不存在 | 不存在 | 中 |
| Zhao | 2023 | Frontiers in Endocrinology | 10.3389/fendo.2023.1161356 | P < 5×10^-8 and minor allele frequency > 0.01 | TT/BioT/SHBG: r² < 0.001, window = 10000 kb; DHEAS: r² < 0.1, window = 5000 kb | F-statistic | MR-Egger intercept; MR-PRESSO | Cochran Q | weighted median | BMI was analyzed by multivariable Mendelian randomization for outcomes with significant differences; 针对PCOS是否进行了MVMR结果报告不确定 | Bonferroni | 中 |
| Zhang | 2023 | Clin. Exp. Obstet. Gynecol. | 10.31083/j.ceog5009193 | P < 5×10^-8 | r² < 0.01；window = 不确定 | F-statistic | MR-Egger intercept; MR-PRESSO | Cochran Q | leave-one-out; replication analysis; MR-RAPS | 不存在 | 不存在 | 中 |
| Aru | 2023 | Frontiers in Endocrinology | 10.3389/fendo.2023.1326344 | P < 1×10^-5 | r² < 0.001, window = 10000 kb | F-statistic | MR-Egger intercept; MR-PRESSO | Cochran Q | leave-one-out | 不存在 | 未明确报告 | 中 |
| Min | 2024 | Frontiers in Endocrinology | 10.3389/fendo.2023.1282056 | 主要写明P < 5×10^-6；结果部分对AMH和Estradiol又写P < 5×10^-8，存在不一致 | r² < 0.001, window = 10000 kb | F-statistic | MR-Egger intercept; MR-PRESSO global test | Cochran Q | leave-one-out | 不存在 | 不存在 | 中 |
| Liu | 2022 | BMC Medicine | 10.1186/s12916-022-02238-y | Exposure IVs selected as independent GWAS signals at stringent P-threshold of 5×10^-9 for adult traits; childhood BMI top associated SNPs reaching 5×10^-8; reverse MR used 14 PCOS-associated variants at 5×10^-8 | Adult obesity GWAS clumping: P<1×10^-9 and LD window ±5 Mb (r2 > 0.05) to obtain clumps; cross-trait meta-analysis used PLINK clumping --clump-r2 0.2 --clump-kb 500; MR-specific clumping parameters not fully described | F-statistic | MR-Egger intercept; GWAS catalog screening for pleiotropic SNPs | 不存在 | leave-one-out; MR-Clust | 不存在 | Bonferroni | 中 |
| Actkins | 2022 | Journal of the Endocrine Society | 10.1210/jendso/bvac150 | 不确定 | 不确定 | 不确定 |  |  |  | 报告了mediation analysis，涉及BMI、free testosterone、total testosterone和T2D；其余不确定 | 不确定 | 低 |
| Zhang | 2022 | Clinical and Experimental Obstetrics & Gynecology | 10.31083/j.ceog4909199 | P < 5 × 10^-8 | r² < 0.001, window < 1 Mb | F-statistic | MR-Egger intercept; MR-PRESSO | MR-PRESSO global test | Steiger test; leave-one-out | 不存在 | 不存在 | 中 |
| He | 2022 | Frontiers in Genetics | 10.3389/fgene.2022.850892 | P < 5×10^-8 | r² < 0.001, window = 10000 kb | F-statistic | MR-Egger intercept; MR-PRESSO | Cochran Q; I²; funnel plot | leave-one-out; replication analysis | BMI | Bonferroni | 中 |
| De Silva | 2022 | PLOS ONE | 10.1371/journal.pone.0269191 | P < 5×10^-8 | r² < 0.001, window = 10000 kb; proxy SNP minimum LD-r² = 0.8 | F-statistic | MR-Egger intercept; MR-PRESSO; MR-PRESSO global test; Radial MR | Cochran Q; funnel plot | leave-one-out; weighted median; weighted mode | 存在；multivariable MR adjusted for combinations of HC, WC, BMI, weight, and WHR depending on model | Bonferroni | 高 |
| Verdiesen | 2022 | Human Reproduction | 10.1093/humrep/deac028 | P < 5×10^-8 | 不存在 | F-statistic | comparison of IVW and SNP-specific Wald ratio estimates | Cochran Q | leave-one-out | 不存在 | 不存在 | 中 |
| Simons | 2021 | Clinical Endocrinology | 10.1111/cen.14593 | P < 5×10^-8 | r² > 0.1 exclusion for LD; palindromic variants with minor allele frequency > 0.42 excluded | F-statistic | MR-Egger intercept; MR-PRESSO | Cochran Q | Steiger test | 不存在 | 不存在 | 中 |
| Gan | 2021 | The Journal of Clinical Endocrinology and Metabolism | 10.1210/clinem/dgab843 | P < 5×10^-8 | r² < 0.001, window = 10000 kb | F-statistic | MR-Egger intercept; MR-PRESSO; funnel plot | Cochran Q; I²; funnel plot | leave-one-out; Radial MR; weighted median; weighted mode; MR-Egger SIMEX | 不存在 | 不存在 | 中 |
| Sun | 2022 | Reproductive Sciences | 10.1007/s43032-021-00776-z | PeQTL < 5×10^-8; MAF > 0.01 | removing SNPs in very strong LD (r² > 0.9) with the top associated eQTL, and removing SNPs in low LD or not in LD (r² < 0.05) with the top associated eQTL | 不存在 | HEIDI test | HEIDI test |  | 不存在 | FDR | 中 |
| Yan | 2022 | Endocrine | 10.1007/s12020-021-02867-y | P < 5×10^-8 | r² > 0.1 excluded；window = 不确定 | 不存在 | MR-Egger intercept; MR-PRESSO |  | leave-one-out | 不存在 | Bonferroni | 中 |
| Deshmukh | 2022 | Bone | 10.1016/j.bone.2021.116285 | 不确定 | 不确定 | 不存在 | MR-Egger intercept |  |  | 不存在 | 不存在 | 中 |
| Martin | 2022 | eLife | https://doi.org/10.7554/eLife.72452 | P < 5×10^-8 | 不确定 | 不存在 | MR-Egger | Cochran Q; I² | weighted median; replication analysis | 不存在 | FDR | 中 |
| Chen | 2021 | Frontiers in Endocrinology | 10.3389/fendo.2021.756137 | P < 5×10^-8 | r² < 0.001；另外检查与混杂因素相关信号时使用 r² > 0.2 | F-statistic | MR-Egger intercept; MR-PRESSO; MR-PRESSO global test | Cochran Q | weighted median; weighted mode; simple mode | 不存在 | 不存在 | 中 |
| Lim | 2021 | Genes & Genomics | 10.1007/s13258-021-01134-8 | Suggestive association P < 10^-5 for insulin GWAS variants; additional exclusion of SNPs nominally associated with PCOS or confounding factors | 不确定 | 不存在 |  |  |  | 不存在 | 不存在 | 低 |
| Chen | 2021 | Frontiers in Endocrinology | 10.3389/fendo.2021.731569 | 暴露侧主要为 P < 5×10^-8，因仅9个炎症因子满足>2个独立SNP，进一步采用 P < 5×10^-6；PCOS工具变量为 P < 5×10^-8 | 暴露侧 r² < 0.1；PCOS侧 r² < 0.01；window = 不确定 | F-statistic | MR-Egger intercept; MR-PRESSO | Cochran Q | weighted median; simple mode; weighted mode; bidirectional MR | 不存在 | 不存在 | 中 |
| Kho | 2021 | Human Genetics | 10.1007/s00439-021-02312-0 | P < 5×10^-8 | r² < 0.01 | 不存在 | MR-Egger intercept; GSMR HEIDI outlier test | Cochran Q | leave-one-out | 不存在 | Bonferroni | 中 |
| Martin | 不确定 | 不确定 | 不确定 | P < 5×10^-8 | 不确定 | 不存在 | MR-Egger | I² | weighted median | 不存在 | 未明确报告 | 中 |
| Jin | 2021 | Frontiers in Genetics | 10.3389/fgene.2021.689897 | P < 5×10^-8；reverse MR for OCD relaxed to P < 5×10^-6 | r² = 0.001, window = 10000 kb | F-statistic | MR-Egger intercept | Cochran Q; funnel plot | leave-one-out | 不存在 | Bonferroni | 中 |
| Wu | 2021 | Frontiers in Genetics | 10.3389/fgene.2021.644101 | PCOS instruments: P < 5×10^-8; periodontitis instruments: P < 5×10^-6 | R² > 0.01 within 1 Mb window examined; variant with the lowest P-value at each locus retained | F-statistic | MR-Egger intercept; MR-PRESSO; MR-PRESSO global test | Cochran Q; funnel plot | leave-one-out | 不存在 | Bonferroni | 高 |
| Wen | 2021 | Breast Cancer Research and Treatment | 10.1007/s10549-020-05973-z | P < 5×10^-8 | r² < 0.001 | 不存在 | MR-Egger intercept | Cochran Q | leave-one-out; weighted median | 不存在 | 不存在 | 中 |
| Tao | 2021 | Clinical Epidemiology | 不确定 | genome-wide significance threshold | r² > 0.1 used for exclusion of SNPs in high LD | F-statistic | MR-Egger intercept; MR-PRESSO | Cochran Q | replication analysis | 不存在 | 不存在 | 中 |
| Sun | 2020 | Frontiers in Endocrinology | 10.3389/fendo.2020.00621 | P < 1 × 10^-5 | r² < 0.1, window = 500 kb | F-statistic/R² | MR-Egger; MR-PRESSO; MR-PRESSO global test |  | weighted median | 不存在 | 不存在 | 中 |
| Zhao | 2020 | The Journal of Clinical Endocrinology & Metabolism | 10.1210/clinem/dgaa125 | BMI instruments selected from top significant variants in BBJ GWAS; exact genome-wide significance threshold not explicitly stated in this article | PLINK clump using 1000 Genomes East Asian reference; same region defined as within 500 kb; linkage disequilibrium mentioned as r2>0.2 for many loci, but formal clumping threshold not clearly reported | 不存在 | MR-Egger intercept; funnel plot | 不存在 | leave-one-out | 不存在 | Bonferroni | 中 |
| Zhu | 2020 | Journal of the Endocrine Society | 10.1210/jendso/bvaa046 | 不确定 | 不确定 | 不存在 | MR-Egger |  |  | 不存在 | 不确定 | 中 |
| Ahn | 2020 | Diabetes, Metabolic Syndrome and Obesity: Targets and Therapy | 10.2147/DMSO.S281529 | GWAS suggestive association P < 1×10^-6 for obesity-trait SNP selection; discovery stage forward selection P < 0.05 | 不确定 | 不存在 | 不存在 | 不存在 | 不存在 | 不存在 | 不存在 | 低 |
| Harris | 2019 | International Journal of Epidemiology | 10.1093/ije/dyz113 | P < 5×10^-8 | Reported as none in linkage disequilibrium; exact r² threshold/window not reported | 不存在 | MR-Egger intercept | Cochran Q | weighted median | 不存在 | 不存在 | 中 |
| Yarmolinsky | 2019 | PLoS Medicine | 10.1371/journal.pmed.1002893 | P < 5×10^-8 | r² < 0.001, window = 10000 kb | F-statistic/R² | MR-Egger intercept | 不存在 | Steiger test; leave-one-out; weighted median; weighted mode | 不存在 | Bonferroni | 中 |
| Day | 2018 | PLoS Genetics | 10.1371/journal.pgen.1007813 | 不确定 | 不确定 | 不存在 | MR-Egger intercept | 不存在 | 不存在 | 不存在 | Bonferroni | 低 |
| Day | 2015 | Nature Communications | 10.1038/ncomms9464 | 基于reported GWAS signals for 15 traits；具体SNP纳入显著性阈值不确定 | 不确定 | 不存在 | 不确定（通过individual SNP consistency检查pleiotropy） | funnel plot | scatter plot | 不存在 | Bonferroni | 中 |
| Wen | 2025 | The World Journal of Biological Psychiatry | 10.1080/15622975.2025.2555173 | P < 5×10^-8 | r² < 0.001, window = 10000 kb | F-statistic/R² | MR-Egger intercept; MR-PRESSO | Cochran Q; funnel plot | leave-one-out | 不存在 | 未明确报告 | 中 |
| Niu | 2025 | Gynecologic and Obstetric Investigation | 10.1159/000546487 | P < 5×10^-8 | r² < 0.001, window = 10000 kb | F-statistic | MR-Egger intercept | Cochran Q | leave-one-out | 不存在 | 未明确报告 | 中 |
| Si | 2023 | Frontiers in Endocrinology | 10.3389/fendo.2023.1229900 | P < 5×10^-8 | r² < 0.01, window = 10000 kb | F-statistic/R² | MR-Egger intercept; MR-PRESSO | Cochran Q; funnel plot | leave-one-out | 不存在 | 不存在 | 中 |
| Wang | 2025 | International Journal of Women's Health | 不确定 | P < 5×10^-8 | r² < 0.001, window = 10000 kb | F-statistic | MR-Egger intercept; MR-PRESSO; MR-PRESSO global test | Cochran Q; funnel plot | leave-one-out | 不存在 | 不存在 | 中 |
| Jiang | 2024 | Multiple Sclerosis Journal | 10.1177/13524585241292802 | 不确定 | 不确定 | 不存在 | 不确定（正文提及 explored horizontal pleiotropy，但未明确 MR 中具体方法） | 不确定 | bidirectional analysis; remove obesity-related SNPs; sensitivity analyses | 不存在 | 不存在 | 中 |
| Fang | 2024 | Journal of Clinical Endocrinology and Metabolism | 10.1210/clinem/dgae446 | P < 5×10^-8; outcome MAF > 0.01 | r² < 0.001, window = 10000 kb | F-statistic | MR-Egger intercept; MR-PRESSO | Cochran Q | leave-one-out | 不存在 | 不存在 | 中 |
| Wang | 2025 | Advanced Science | 10.1002/advs.202411679 | P < 5 × 10^-8; eQTL follow-up analyses used P < 5 × 10^-4; cis-pQTL used P < 5 × 10^-8 | r² < 0.001, window = 10000 kb; eQTL r² < 0.1; pQTL pruning r² > 0.01 removed | F-statistic | MR-Egger intercept; MR-PRESSO |  | Steiger test; replication analysis; weighted median; MR-PRESSO; MR-Egger | Multivariable MR adjusted for bioavailable testosterone or total testosterone | Bonferroni/FDR | 中 |
| Chen | 2024 | Journal of Ovarian Research | 10.1186/s13048-024-01486-1 | P < 1×10^-5 | r² < 0.1, window = 500 kb | F-statistic | MR-Egger intercept; MR-PRESSO | Cochran Q | Steiger test; replication analysis; Radial MR | 不存在 | 不存在 | 中 |
| Zhang | 2024 | Combinatorial Chemistry & High Throughput Screening | 10.2174/0113862073325251241101054306 | P < 5e-08 | r² ≤ 0.001, window = 10000 kb | F-statistic/R² | MR-Egger intercept; funnel plot | Cochran Q; funnel plot | leave-one-out | 不存在 | 不存在 | 中 |
| Xiao | 2023 | Journal of the American Heart Association | 10.1161/JAHA.121.026561 | 2-sample MR used genome-wide significant SNPs with P < 5×10^-8; female-specific traits for bidirectional MR required at least 1 genome-wide significant SNP after pruning | r² = 0.1, window = 250 kb | F-statistic | MR-Egger intercept |  | weighted median; MR-Egger; bidirectional MR | 不存在 | FDR | 中 |
| Peng | 2025 | International Journal of Gynecology & Obstetrics | 10.1002/ijgo.16057 | P < 5×10^-8；为获得足够SNP（number ≥3）时部分ORRD也采用 P < 5×10^-6 | r² < 0.001, window = 10000 kb | F-statistic | MR-Egger intercept | Cochran Q | leave-one-out; replication analysis | 不存在 | 不存在 | 中 |
| Su | 2024 | Frontiers in Endocrinology | 10.3389/fendo.2024.1393847 | P < 5×10^-8; MAF ≥ 0.01 | r² < 0.001, window = 10000 kb | F-statistic/R² | MR-Egger intercept; MR-PRESSO | Cochran Q; funnel plot | leave-one-out | 不存在 | 不存在 | 中 |
| Wang | 2024 | Frontiers in Endocrinology | 10.3389/fendo.2024.1391826 | P < 1×10^-5 | r² < 0.01, window = 500 kb | F-statistic | MR-Egger intercept; MR-PRESSO | Cochran Q; funnel plot | Steiger test; leave-one-out; replication analysis | 不存在 | 不存在 | 中 |
| Du | 2023 | Frontiers in Endocrinology | 10.3389/fendo.2023.1120119 | P < 5×10^-8 | r² < 0.001 | F-statistic | MR-Egger intercept | Cochran Q | weighted median; simple mode; weighted mode | 不存在 | 不存在 | 中 |
| Zhao | 2023 | Frontiers in Endocrinology | 10.3389/fendo.2023.1295412 | LDL-C关联SNP达到genome-wide significance；MAF > 1% | r² < 0.3, window = 100 kb | 不存在 | MR-Egger intercept; MR-PRESSO | Cochran Q | leave-one-out; replication analysis | 不存在 | 不存在 | 中 |
| Ni | 2024 | The American Journal of Human Genetics | 10.1016/j.ajhg.2024.10.008 | P < 5×10^-8 | r² < 0.1, window = 5000 kb | F-statistic/R² | MR-Egger intercept; MR-PRESSO | HEIDI test | weighted median; weighted mode; simple mode; replication analysis; colocalization analysis; SMR | 不存在 | FDR | 中 |
| Min | 2023 | Frontiers in Microbiology | 10.3389/fmicb.2023.1203902 | P < 1×10^-5 | r² = 0.001, window = 10000 kb | F-statistic | MR-Egger intercept; MR-PRESSO global test | Cochran Q | leave-one-out | 不存在 | FDR | 中 |
| Cao | 2023 | Journal of Ovarian Research | 10.1186/s13048-023-01306-y | P < 5×10^-8 | r² < 0.001, window = 10000 kb | F-statistic | MR-Egger intercept; MR-PRESSO | Cochran Q | MR-PRESSO | 中介变量包括age at menopause、IGF-1、triglycerides；MVMR中对PCOS进行调整 | 不存在 | 中 |
| Yan | 2022 | Endocrine | 10.1007/s12020-021-02867-y | P < 5×10^-8 | r² > 0.1 excluded；window = 不确定 | 不存在 | MR-Egger intercept; MR-PRESSO |  | leave-one-out | 不存在 | Bonferroni | 中 |
| Harris | 2019 | International Journal of Epidemiology | 10.1093/ije/dyz113 | P < 5×10^-8 | No linkage disequilibrium among included SNPs; specific r²/window not reported | 不存在 | MR-Egger intercept | Cochran Q | weighted median | 不存在 | 不存在 | 中 |
| Tao | 2025 | International Journal of Women’s Health | 不确定 | P < 1e−5 | r² < 0.1, window = 500 kb | F-statistic | MR-Egger intercept; MR-PRESSO | Cochran Q; funnel plot | leave-one-out; weighted median | 不存在 | FDR | 中 |
| Sun | 2024 | Frontiers in Endocrinology | 10.3389/fendo.2024.1280983 | P < 1.0×10^-5 | r² < 0.001, window = 10000 kb | F-statistic | MR-Egger intercept; MR-PRESSO | Cochran Q | leave-one-out | 不存在 | Bonferroni | 中 |
| Shao | 2026 | Research | 10.34133/research.1131 | LST: P < 5×10^-8; SDW/SDC/DPA: P < 1×10^-5 | GCTA-COJO with 10 Mb LD distance; exact r² threshold not clearly reported | F-statistic | MR-Egger intercept; MR-PRESSO | Cochran Q | Steiger test; leave-one-out; replication analysis; MR-RAPS; MR-ROBUST; dIVW | BMI; additionally sex hormones were assessed in MVMR pleiotropy analyses | Bonferroni | 高 |
| Motafeghi | 2025 | International Journal of Endocrinology and Metabolism | 10.5812/ijem-159124 | P < 5×10^-8 | r² < 0.001, window = 10000 kb | F-statistic | MR-Egger intercept; MR-PRESSO; PhenoScanner | Cochran Q; I²; funnel plot; Rucker’s Q | leave-one-out | 不存在 | 不存在 | 高 |
| Fu | 2025 | Am J Transl Res | 10.62347/PGYY9493 | P < 5e-06 for most SNPs across four sports data categories | r² < 0.01, window = 5000 kb | F-statistic | MR-Egger; MR-PRESSO | funnel plot | leave-one-out | 不存在 | 不存在 | 中 |
| Bi | 2024 | Breast Cancer Research | 10.1186/s13058-024-01923-5 | 不确定 | 不存在 | 不存在 | 不存在 | 不存在 | 不存在 | 不存在 | Bonferroni | 中 |
| Qu | 2025 | International Journal of Women's Health | 不确定 | Circulating inflammatory proteins: P < 1×10^-5; reverse MR for ovarian-related diseases: P < 5×10^-8 | r² < 0.001, window = 10000 kb | F-statistic | MR-Egger intercept; MR-PRESSO | Cochran Q | Steiger test; leave-one-out | 不存在 | 不存在 | 中 |
| Ahn | 2020 | Diabetes, Metabolic Syndrome and Obesity: Targets and Therapy | 10.2147/DMSO.S281529 | Discovery stage SNPs with P < 0.05 were taken forward; obesity-associated loci for MR were suggestive loci with P < 1×10^-6 | 不确定 | 不存在 |  |  |  | 不存在 | 未明确报告 | 低 |
| Alesi | 2024 | Biomolecules | 10.3390/biom14121544 | P < 5×10^-6 | r² < 0.01, window = 10000 kb | F-statistic | MR-Egger intercept | Cochran Q; funnel plot | leave-one-out | 不存在 | Bonferroni | 中 |
| Chen | 2021 | Frontiers in Endocrinology | 10.3389/fendo.2021.756137 | P < 5×10^-8 | r² < 0.001 | F-statistic | MR-Egger intercept; MR-PRESSO | Cochran Q | weighted median; weighted mode; simple mode | 不存在 | 不存在 | 中 |
| Ling | 2024 | Scientific Reports | 10.1038/s41598-024-55993-8 | P < 5×10^-8; reverse MR used relaxed threshold P < 5×10^-6 for traits with limited SNPs (≤ 3) after LD pruning | r² < 0.001, window = 10000 kb | F-statistic/R² | MR-Egger intercept; MR-PRESSO | Cochran Q; funnel plot | leave-one-out | 不存在 | Bonferroni | 中 |
| Zhao | 2023 | Frontiers in Endocrinology | 10.3389/fendo.2023.1161356 | GWAS P < 5×10^-8 and minor allele frequency > 0.01 | TT/BioT/SHBG: r² < 0.001, window = 10000 kb; DHEAS: r² < 0.1, window = 5000 kb | F-statistic | MR-Egger intercept; MR-PRESSO | Cochran Q | weighted median | BMI | Bonferroni | 中 |
| Xu | 2026 | Frontiers in Medicine | 10.3389/fmed.2026.1747593 | Methods section: P < 5×10^-8; Results section also states genomewide significance screening at p < 1×10^-5 | r² = 0.001, window = 10000 kb | F-statistic | MR-Egger intercept | Cochran Q; funnel plot | leave-one-out | 不存在 | 不存在 | 中 |
| Wan | 2024 | Gynecological Endocrinology | 10.1080/09513590.2024.2444380 | Forward MR: P < 5×10^-8; reverse MR: threshold adjusted to P < 1×10^-5 | r² < 0.001, window = 10000 kb | F-statistic | MR-Egger intercept; MR-PRESSO | Cochran Q; funnel plot | leave-one-out; replication analysis; scatter plot | 不存在 | Bonferroni | 高 |
| Zheng | 2026 | International Journal of Gynecology and Obstetrics | 10.1002/ijgo.70411 | P < 5×10^-8 | r² < 0.01, window = 10000 kb | F-statistic | MR-Egger intercept; MR-PRESSO | Cochran Q; funnel plot | leave-one-out; replication analysis | 不存在 | 不存在 | 中 |
| Sun | 2022 | Reproductive Sciences | 10.1007/s43032-021-00776-z | PeQTL < 5×10^-8; MAF > 0.01 | removing SNPs in very strong LD (r² > 0.9) with the top associated eQTL, and removing SNPs in low LD or not in LD (r² < 0.05) with the top associated eQTL | 不存在 | HEIDI test | HEIDI test |  | 不存在 | FDR | 中 |
| Gao | 2024 | Phenomics | 10.1007/s43657-024-00156-y | P < 5×10^-8 | r² = 0.001, window = 10000 kb | F-statistic | MR-Egger intercept | Cochran Q | leave-one-out | 不存在 | 不存在 | 中 |
| Chen | 2021 | Frontiers in Endocrinology | 10.3389/fendo.2021.731569 | 暴露端主要为 P < 5×10^-8，因仅17个指标达到该阈值，后改用 P < 5×10^-6；PCOS工具变量为 P < 5×10^-8 | 暴露端 r² < 0.1；PCOS端 r² < 0.01；window = 不确定 | F-statistic | MR-Egger intercept; MR-PRESSO | Cochran Q | weighted median; simple mode; weighted mode; bidirectional MR | 不存在 | 不存在 | 中 |
| Chen | 2024 | Frontiers in Nutrition | 10.3389/fnut.2024.1434648 | p < 1.0 × 10^-5 or p < 5.0 × 10^-8 | r² < 0.001, window = 10000 kb; proxy SNPs allowed with r² > 0.8 | F-statistic | MR-Egger intercept; MR-PRESSO | Cochran Q | leave-one-out; reverse causality analysis | BMI-adjusted PCOS GWAS used; no formal MVMR adjustment model reported for extracted topic-related chains | FDR | 中 |
| Ma | 2025 | Scientific Reports | 10.1038/s41598-025-86794-2 | P < 5×10^-6 | r² < 0.001, window = 10000 kb | F-statistic | MR-Egger intercept; MR-PRESSO | Cochran Q; funnel plot | Steiger test; leave-one-out; reverse MR | BMI, obesity, diabetes, smoking, alcohol use, physical activity, inflammation, depression, waist-hip ratio, vitamin D, dehydroepiandrosterone sulphate, sex hormone binding globulin, testosterone; mediation analysis also examined vitamin D deficiency, visceral adipose tissue mass, BMI adjusted waist-hip ratio and other mediators | FDR | 中 |
| Day | 2018 | PLoS Genetics | 10.1371/journal.pgen.1007813 | 不确定 | 不确定 | 不存在 | MR-Egger intercept | 不存在 | 不存在 | 不存在 | Bonferroni | 中 |
| Jin | 2021 | Frontiers in Genetics | 10.3389/fgene.2021.689897 | 主要为 P < 5×10^-8；反向MR中OCD因无达阈值SNP而放宽至 P < 5×10^-6 | r² = 0.001, window = 10000 kb | F-statistic | MR-Egger intercept | Cochran Q; funnel plot | leave-one-out | 不存在 | Bonferroni | 中 |
| Zhao | 2024 | Front. Endocrinol. | 10.3389/fendo.2024.1364157 | Forward MR: P < 5×10^-8; reverse MR: P < 5×10^-6 | r² < 0.001, window = 10000 kb | F-statistic | MR-Egger intercept; MR-PRESSO; funnel plot | Cochran Q; funnel plot | leave-one-out | 不存在 | Bonferroni | 中 |
| Zhou | 2026 | Journal of Ovarian Research | 10.1186/s13048-026-02026-9 | P < 5.0×10^-6 | r² < 0.001, window = 10000 kb, 1000G EUR reference panel | F-statistic | MR-Egger intercept; MR-PRESSO | Cochran Q | leave-one-out; BWMR; reverse MR | 不存在 | FDR | 中 |
| Wen | 2021 | Breast Cancer Research and Treatment | 10.1007/s10549-020-05973-z | P < 5×10^-8 | R2 < 0.001 | 不存在 | MR-Egger intercept | Cochran Q | leave-one-out | 不存在 | 不存在 | 中 |
| You | 2026 | International Journal of Women's Health | 10.2147/IJWH.S586045 | PCOS, diabetes, BMI, and HRT: P < 5×10^-8; oral contraceptive use: P < 5×10^-7 | r² < 0.001, window = 10000 kb | F-statistic | MR-Egger intercept; MR-PRESSO | Cochran Q; funnel plot | leave-one-out | Adjusted for diabetes, BMI, hormone replacement therapy use, and oral contraceptive use | 不存在 | 中 |
| Luo | 2026 | International Journal of Gynecology and Obstetrics | 10.1002/ijgo.70897 | P < 5×10^-8 | r² < 0.01, window = 10 Mb | F-statistic | MR-Egger intercept; MR-PRESSO | Cochran Q; funnel plot | leave-one-out | 不存在 | Bonferroni | 中 |
| Shen | 2024 | Reproductive Sciences | 10.1007/s43032-024-01476-0 | P < 5×10^-8 | r² < 0.01, window = 1000 kb | 不存在 | MR-Egger intercept; MR-PRESSO | Cochran Q | leave-one-out | 不存在 | 不存在 | 中 |
| He | 2026 | International Journal of Biological Sciences | 10.7150/ijbs.116803 | P < 5.00E-8 or 5.00E-6 | r² < 0.001, window = 10000 kb | 不存在 | MR-Egger intercept; MR-PRESSO | Cochran Q; I²; funnel plot | leave-one-out | MVMR performed after adjusting for the major determinants of exposure and outcome；具体调整变量未明确 | Bonferroni | 中 |
| Guo | 2024 | Gynecological Endocrinology | 10.1080/09513590.2024.2420963 | P < 1×10^-5 | r² < 0.001, window = 10000 kb | F-statistic | MR-Egger intercept; MR-PRESSO | Cochran Q; funnel plot | leave-one-out; bidirectional MR | 中介变量：1-stearoyl-2-oleoyl-GPI (18:0/18:1) levels；采用中介MR计算beta1、beta2、mediated effect和mediated proportion | 不存在 | 中 |
| Wang | 2024 | Fertility and Sterility | 10.1016/j.fertnstert.2024.02.011 | P < 5×10^-8 | r² < 0.001, window = 不确定 | F-statistic | MR-Egger intercept | Cochran Q; I² | leave-one-out | 不存在 | 不存在 | 中 |
| Wang | 2026 | Medicine | 10.1097/MD.0000000000048704 | P < 5×10^-8 | r² < 0.01；window = 不确定 | F-statistic | MR-Egger intercept | Cochran Q; funnel plot | leave-one-out; replication analysis | 不存在 | 不存在 | 中 |
| Chen | 2026 | Journal of Obstetrics and Gynaecology Research | 10.1111/jog.70233 | 主要为 P < 5×10^-8；sleep apnea syndrome 放宽为 P < 5×10^-6 | r² < 0.001, window > 10000 kb | F-statistic | MR-Egger intercept; MR-PRESSO | Cochran Q | leave-one-out | Obesity (BMI, WHR, WC, HC) and insulin resistance represented by fasting insulin | 未明确报告 | 中 |
| Liu | 2025 | Journal of Ovarian Research | 10.1186/s13048-025-01617-2 | P < 5×10^-6 | r² < 0.001, window = 10000 kb | 不存在 | MR-Egger intercept; MR-PRESSO | Cochran Q; funnel plot | Steiger test; leave-one-out | 不存在 | 不存在 | 中 |
| Wei | 2025 | Eating and Weight Disorders - Studies on Anorexia, Bulimia and Obesity | 10.1007/s40519-025-01801-9 | P < 5×10^-6 | r² < 0.0001, window = 10000 kb | F-statistic | MR-Egger intercept | Cochran Q; funnel plot | leave-one-out | 不存在 | FDR | 中 |
| Simons | 2021 | Clinical Endocrinology | 10.1111/cen.14593 | P < 5×10^-8 | r² > 0.1 excluded; SNP with largest absolute effect estimate retained | F-statistic | MR-Egger intercept; MR-PRESSO | Cochran Q | Steiger test; MR-PRESSO; weighted median; simple median | 不存在 | 不存在 | 中 |
| Wang | 2026 | Ecotoxicology and Environmental Safety | 10.1016/j.ecoenv.2026.119742 | EDC暴露SNP初始阈值 P < 5×10^-8；因无SNP保留，最终采用 P < 5×10^-6；reverse MR采用 P < 5×10^-8 | r² < 0.001, window = 10000 kb | F-statistic | MR-Egger intercept; MR-PRESSO | Cochran Q | BWMR; weighted median; MR-Egger | 不存在 | FDR | 中 |
| Gao | 2025 | Endocrine, Metabolic & Immune Disorders - Drug Targets | 10.2174/0118715303363459250317084005 | P < 5×10^-6 | r² < 0.001, window = 10000 kb | F-statistic | MR-Egger intercept; MR-PRESSO | Cochran Q; funnel plot | leave-one-out | 不存在 | 不存在 | 中 |
| Jiang | 2025 | Medicine | 10.1097/MD.0000000000041359 | Exposure SNPs: P < 5×10^-8 initially, relaxed to P < 5×10^-6; PCOS SNPs: P < 5×10^-8 | Cytokines: r² < 0.001, window = 250 kb; PCOS: r² < 0.001, window = 10000 kb | F-statistic | MR-Egger intercept; MR-PRESSO | Cochran Q | leave-one-out; replication analysis | 不存在 | FDR | 中 |
| Jiang | 2026 | Ecotoxicology and Environmental Safety | 10.1016/j.ecoenv.2026.119900 | p < 1e-08 | r² < 0.1, window = 1000 kb | F-statistic | MR-Egger intercept | Cochran Q | weighted median; simple mode; weighted mode | 不存在 | Bonferroni | 中 |
| Yu | 2025 | Health Science Reports | 10.1002/hsr2.71285 | P < 5×10^-8 | r² < 0.001, window = 10000 kb | F-statistic | MR-Egger intercept; MR-PRESSO | Cochran Q; MR-PRESSO global test | weighted median; simple mode; weighted mode; SMR; HEIDI; colocalization analysis | 不存在 | FDR | 中 |
| Zhang | 2022 | Clinical and Experimental Obstetrics & Gynecology | 10.31083/j.ceog4909199 | P < 5 × 10^-8 | r² < 0.001, window = 1 Mb | F-statistic | MR-Egger intercept; MR-PRESSO | MR-PRESSO global test | Steiger test; leave-one-out | 不存在 | 不存在 | 中 |
| Tian | 2024 | Journal of Ovarian Research | 10.1186/s13048-024-01525-x | P < 1×10^-5 | r² < 0.001, window = 10000 kb | F-statistic | MR-Egger intercept; MR-PRESSO | Cochran Q | weighted median; weighted mode; simple mode | 不存在 | 不存在 | 中 |
| Aru | 2023 | Frontiers in Endocrinology | 10.3389/fendo.2023.1326344 | P < 1×10^-5 | r² < 0.001, window = 10000 kb | F-statistic/R² | MR-Egger intercept; MR-PRESSO; GWAS Catalog search for pleiotropic SNPs | Cochran Q | leave-one-out | 不存在 | 未明确报告 | 中 |
| Yang | 2026 | Medicine | 10.1097/MD.0000000000049143 | P < 5×10^-6 | r² < 0.001, window = 10000 kb | F-statistic/R² | MR-Egger intercept; funnel plot | Cochran Q; funnel plot | Steiger test; leave-one-out | 不存在 | 不存在 | 中 |
| Liu | 2023 | Frontiers in Endocrinology | 10.3389/fendo.2023.1140499 | P < 5×10^-8 | r² < 0.001, window = 10000 kb | F-statistic | MR-Egger intercept; MR-PRESSO; MR-PRESSO global test | Cochran Q | leave-one-out; replication analysis; weighted median; weighted mode; MR-Egger; MR-PRESSO | 不存在 | 不存在 | 高 |
| Shao | 2025 | European Journal of Obstetrics & Gynecology and Reproductive Biology | 10.1016/j.ejogrb.2024.12.043 | P < 5×10^-8 | r² < 0.001 | F-statistic | MR-Egger intercept; MR-PRESSO | Cochran Q; funnel plot | leave-one-out; replication analysis | 不存在 | 不存在 | 中 |
| Xu | 2025 | Frontiers in Nutrition | 10.3389/fnut.2025.1644496 | lipids: P < 5×10^-8; PCOS: P < 5×10^-6 | r² < 0.001; window = 不确定 | F-statistic | MR-Egger intercept; MR-PRESSO | Cochran Q | leave-one-out | multivariable MR adjusted for HDL, LDL, and TG jointly; confounder-associated IVs identified via PhenoScanner and excluded in reevaluation | 不存在 | 中 |
| Kho | 2021 | Human Genetics | 10.1007/s00439-021-02312-0 | P < 5×10^-8 | r² < 0.01 | 不存在 | MR-Egger intercept; GSMR HEIDI outlier test | Cochran Q | leave-one-out | 不存在 | Bonferroni | 中 |
| Wu | 2021 | Frontiers in Genetics | 10.3389/fgene.2021.644101 | PCOS instruments: P < 5×10^-8; periodontitis instruments: P < 5×10^-6 | R2 > 0.01 within 1 Mb window was examined; variants with the lowest P-value at each locus were retained | F-statistic | MR-Egger intercept; MR-PRESSO | Cochran Q; funnel plot | leave-one-out | 不存在 | Bonferroni | 中 |
| Gan | 2021 | The Journal of Clinical Endocrinology and Metabolism | 10.1210/clinem/dgab843 | P < 5×10^-8 | r² < 0.001, window = 10000 kb | F-statistic | MR-Egger intercept; MR-PRESSO; funnel plot | Cochran Q; I²; funnel plot | leave-one-out; Radial MR | 不存在 | 不存在 | 中 |
| Chen | 2023 | Human Genomics | 10.1186/s40246-023-00550-z | P < 5×10^-8 | r² < 0.001, window = 10 mb | F-statistic | MR-Egger intercept; MR-PRESSO | Cochran Q; funnel plot | leave-one-out; replication analysis | 不存在 | 不存在 | 中 |
| Gao | 2024 | Medicine | 10.1097/MD.0000000000040115 | gut microbiota IVs: P < 1×10^-5; risk factors IVs: P < 5×10^-8 | r² < 0.001, window > 10000 kb | 不存在 | MR-Egger intercept; MR-PRESSO | 不确定 | weighted median; maximum likelihood; Radial MR; MR-PRESSO | 中介变量：BMI, WC | Bonferroni | 中 |
| Mu | 2023 | eBioMedicine | 10.1016/j.ebiom.2023.104492 | P < 5×10^-8 | 不确定 | F-statistic/R² | MR-Egger intercept; MR-PRESSO | Cochran Q; funnel plot | Steiger test; leave-one-out; weighted median; weighted mode; replication analysis | Multivariable MR included all BCAAs together to assess independent causality; no mediation analysis reported | 未明确报告 | 中 |
| Yarmolinsky | 2019 | PLoS Medicine | 10.1371/journal.pmed.1002893 | P < 5×10^-8 | r² < 0.001, window = 10000 kb | F-statistic/R² | MR-Egger intercept; weighted median; weighted mode | 不存在 | Steiger test; leave-one-out | 不存在 | Bonferroni | 中 |
| Tao | 2021 | Clinical Epidemiology | 不确定 | genome-wide significance threshold | r² > 0.1 exclusion threshold; window = 不确定 | F-statistic/R² | MR-Egger intercept; MR-PRESSO | Cochran Q | weighted median; likelihood-based method; MR-PRESSO; excluding potentially pleiotropic SNPs | 不存在 | 不存在 | 中 |
| Li | 2025 | Frontiers in Endocrinology | 10.3389/fendo.2025.1534733 | P < 5×10^-8 | r² < 0.001, window = 1000 kb for cis-pQTL proximity definition; cis-effects within 1000 kb of corresponding protein-coding sequences | F-statistic | MR-Egger intercept; MR-PRESSO; phenoscanner | Cochran Q | leave-one-out | 不存在 | FDR | 中 |
| Lin | 2024 | International Journal of Endocrinology | 10.1155/2024/3090797 | P < 5×10^-8 | r² < 0.05; proxy search threshold r² > 0.8 for unavailable SNPs | F-statistic | MR-Egger intercept; MR-PRESSO | leave-one-out | Steiger test; leave-one-out; weighted median | 不存在 | 不存在 | 中 |
| Wang | 2026 | International Journal of Endocrinology | 10.1155/ije/3604933 | P < 5×10^-8；若无 genome-wide significant SNPs，则使用 P < 5×10^-6 | r² < 0.001, window = 10000 kb | F-statistic/R² | MR-Egger intercept; MR-PRESSO | Cochran Q | leave-one-out | 中介变量：BMI；进行了 two-step MR mediation analysis | 不存在 | 中 |
| Yang | 2024 | Journal of Ovarian Research | 10.1186/s13048-024-01466-5 | P < 5×10^-8 | r² < 0.001, window = 10000 kb | F-statistic/R² | MR-Egger intercept; MR-PRESSO | Cochran Q; funnel plot | leave-one-out | current tobacco smoking, alcohol consumption, sleeplessness/insomnia, BMI, history of PID | Bonferroni | 中 |
| Chan | 2025 | Endocrinology, Diabetes & Metabolism | 10.1002/edm2.70030 | P < 5×10^-8 | r² < 0.001, window = 10000 kb | F-statistic/R² | MR-Egger intercept | Cochran Q | leave-one-out | 不存在 | 不存在 | 中 |
| Hong | 2025 | F S Sci | 10.1016/j.xfss.2025.02.003 | P < 5×10^-8 | r² < 0.001, window = 10000 kb | 不存在 | MR-Egger intercept; PheWAS | Cochran Q | leave-one-out; weighted median; weighted mode; simple mode; colocalization analysis | 不存在 | 未明确报告 | 中 |
| Zeng | 2024 | Diabetology & Metabolic Syndrome | 10.1186/s13098-024-01404-x | P < 5×10^-8 | r² < 0.001, window = 10000 kb | F-statistic | MR-Egger intercept; MR-PRESSO | Cochran Q | weighted median | 存在；MVMR 同时纳入三种 glycemic traits，mediation analysis 中介变量为 type II diabetes | 不存在 | 中 |
| De Silva | 2022 | PLOS ONE | 10.1371/journal.pone.0269191 | P < 5×10^-8 | r² < 0.001, window = 10000 kb; proxy SNP minimum r² = 0.8; MAF threshold for palindromic SNP alignment = 0.3 | F-statistic | MR-Egger intercept; MR-PRESSO; Radial MR | Cochran Q; funnel plot | leave-one-out; replication analysis 不存在; weighted median; weighted mode; scatter plot; single SNP analysis | 存在；MVMR adjusted for combinations of HC, WC, BMI, Weight, and WHR depending on model; mediation analysis 不存在 | Bonferroni | 高 |
| Zhang | 2025 | Int J Gynecol Obstet | 10.1002/ijgo.16029 | micronutrients: P < 5×10^-8; if no SNPs then relaxed to P < 5×10^-5 | r² = 0.001, window = 10000 kb | F-statistic | MR-Egger intercept; MR-PRESSO | Cochran Q | Steiger test; leave-one-out | 不存在 | FDR | 中 |
| Liu | 2024 | Journal of Thoracic Disease | 10.21037/jtd-24-885 | P < 5×10^-8 | r² < 0.001, window = 10000 kb | F-statistic | MR-Egger intercept | Cochran Q; funnel plot | leave-one-out | 不存在 | 不存在 | 中 |
| Lai | 2025 | Discover Oncology | 10.1007/s12672-025-01861-4 | P < 5×10^-8 | r² < 0.001, window = 不确定 | F-statistic | MR-Egger intercept | Cochran Q; funnel plot | leave-one-out; weighted median | 不存在 | Benjamini–Hochberg false discovery rate | 中 |
| Guixue | 2024 | Frontiers in Endocrinology | 10.3389/fendo.2024.1337562 | P < 5×10^-8 | r² < 0.001, window > 10000 kb | F-statistic | MR-Egger intercept | Cochran Q; funnel plot | leave-one-out | 不存在 | 不存在 | 中 |
| Liu | 2022 | BMC Medicine | 10.1186/s12916-022-02238-y | Exposure IVs were index SNPs identified at genome-wide significance; adult obesity GWAS top-associated SNPs identified at P < 1×10^-9 and childhood BMI combined meta-analysis top SNPs at P < 5×10^-8; reverse MR used 14 PCOS-associated variants reaching P < 5×10^-8 | 不确定 | F-statistic | MR-Egger intercept |  | leave-one-out; MR-Clust | 不存在 | Bonferroni | 中 |
| Xiao | 2026 | Naunyn-Schmiedeberg's Archives of Pharmacology | 10.1007/s00210-026-05473-9 | P < 5×10^-8 | UKB-PPP exposure source reported clumping ± 1 Mb, r² < 0.8; per-chain MR clumping parameters not clearly reported | F-statistic | MR-Egger intercept; HEIDI | Cochran Q | replication analysis | 不存在 | FDR | 中 |
| Chen | 2025 | Medicine | 10.1097/MD.0000000000044448 | P < 5×10^-6; suggestive threshold P < 1×10^-5 when unavailable | r² < 0.001, window = 10000 kb | F-statistic | MR-Egger intercept; MR-PRESSO | Cochran Q; funnel plot | Steiger test; leave-one-out; MR-RAPS | 中介变量为 dopamine 4-sulfate to dopamine 3-O-sulfate ratio | 不存在 | 中 |
| Min | 2024 | Frontiers in Endocrinology | 10.3389/fendo.2023.1282056 | PCOS、LH及反向MR为 P < 5×10^-6；AMH和Estradiol为 P < 5×10^-8 | r² < 0.001, window = 10000 kb | F-statistic | MR-Egger intercept; MR-PRESSO | Cochran Q | leave-one-out | 不存在 | 不存在 | 中 |
| Lu | 2026 | International Journal of Women's Health | 10.2147/IJWH.S593314 | P < 5×10^-8 | r² < 0.001, window = 10 kb | F-statistic | MR-Egger intercept; MR-PRESSO global test | Cochran Q; funnel plot | Steiger test; leave-one-out | 不存在 | 不存在 | 中 |
| Zhao | 2020 | The Journal of Clinical Endocrinology & Metabolism | 10.1210/clinem/dgaa125 | 不确定 | PLINK clump; disequilibrium information based on 1000 Genomes East Asian data; within 500 kb region mentioned; r2>0.2 reported for many loci in same region, but final clumping threshold not clearly reported | 不存在 | MR-Egger intercept; funnel plot |  | leave-one-out | 不存在 | Bonferroni | 中 |
| He | 2022 | Frontiers in Genetics | 10.3389/fgene.2022.850892 | P < 5×10^-8 | r² < 0.001, window = 10000 kb | F-statistic | MR-Egger intercept; MR-PRESSO | Cochran Q; I²; funnel plot | leave-one-out; replication analysis | BMI | Bonferroni | 中 |
| Zhang | 2025 | Medicine | 10.1097/MD.0000000000045566 | P < 5×10^-6 for FST instruments | r² < 0.001, window = 10000 kb | F-statistic/R² | MR-Egger intercept; MR-PRESSO | Cochran Q | leave-one-out | 不存在 | Bonferroni | 高 |
| Xu | 2026 | International Journal of Women's Health | 10.2147/IJWH.S586268 | P < 5×10^-8; for some dietary traits with limited SNPs, relaxed to P < 5×10^-6 | r² < 0.001, window = 10000 kb | F-statistic | MR-Egger intercept; MR-PRESSO | Cochran Q; funnel plot | leave-one-out | 不存在 | 不存在 | 中 |
| Zhang | 2023 | Frontiers in Endocrinology | 10.3389/fendo.2023.1110341 | P < 5×10^-8 | r² < 0.001, window = 10000 kb | F-statistic/R² | MR-Egger intercept; MR-PRESSO | Cochran Q | leave-one-out | Adjusted for obesity indicators (BMI, WHR, WC, HC) and fasting insulin as insulin resistance | 不存在 | 中 |
| Li | 2025 | Immunology & Cell Biology | 10.1111/imcb.70016 | P < 1×10^-5 | r² < 0.001, window = 10000 kb | F-statistic | MR-Egger intercept; MR-PRESSO; MR-PRESSO global test | Cochran Q | leave-one-out; reverse MR | 中介变量为blood metabolites；使用two-step MR和delta method | 不存在 | 中 |
| Lu | 2024 | Frontiers in Endocrinology | 10.3389/fendo.2024.1438079 | P < 1×10^-5 | r² < 0.001, window = 10000 kb | F-statistic | MR-Egger intercept | Cochran Q; I²; funnel plot | leave-one-out | 不存在 | 不存在 | 中 |
| Wang | 2024 | Br J Hosp Med | 10.12968/hmed.2024.0478 | P < 5 × 10^-6 | r² < 0.001, window = 10000 kb | 不存在 | MR-Egger intercept; MR-PRESSO | Cochran Q | leave-one-out; funnel plot; scatter plot | 不存在 | 不存在 | 中 |
| Fu | 2026 | BMC Women's Health | 10.1186/s12905-025-04237-6 | P < 5×10^-8；当常规阈值下独立 SNP 少于5个时，部分膳食暴露采用 P < 5×10^-6 | r² < 0.01, window = 5000 kb | F-statistic/R² | MR-Egger intercept; MR-PRESSO | Cochran Q; funnel plot | leave-one-out | 不存在 | FDR | 中 |
| Day | 2015 | Nature Communications | 10.1038/ncomms9464 | 基于 reported GWAS signals for 15 traits；具体 SNP selection threshold 不确定 | 不确定 | 不存在 | scatter plot; funnel plot | 不确定 | scatter plot; funnel plot | 不存在 | Bonferroni | 中 |
| Zhang | 2023 | Clinical and Experimental Obstetrics & Gynecology | 10.31083/j.ceog5009193 | P < 5×10^-8 | r² < 0.01 | F-statistic | MR-Egger intercept; MR-PRESSO | Cochran Q | leave-one-out; replication analysis; MR-RAPS | 不存在 | 不存在 | 中 |
| Liu | 2024 | Front. Med. | 10.3389/fmed.2024.1429783 | OSAS exposure: P < 5×10^-8; PCOS exposure: P < 5×10^-6 | r² < 0.001, window = 10000 kb | F-statistic | MR-Egger intercept; MR-PRESSO | Cochran Q | leave-one-out | 不存在 | 不存在 | 中 |
| Liang | 2023 | Frontiers in Endocrinology | 10.3389/fendo.2023.1164186 | P < 1.0×10^-5 and P < 5×10^-8 | r² < 0.001, window = 10000 kb | F-statistic/R² | MR-Egger intercept; MR-PRESSO | Cochran Q | leave-one-out | 不存在 | FDR | 中 |
| Jia | 2024 | Frontiers in Medicine | 10.3389/fmed.2024.1433612 | P < 5×10^-6 | r² < 0.001, window = 10000 kb | F-statistic | MR-Egger intercept; MR-PRESSO | Cochran Q | Steiger test; leave-one-out; weighted median | 中介变量：serum metabolites（two-step MR; delta method for 95% CI） | FDR | 中 |
| Fu | 2026 | International Journal of Women's Health | 10.2147/IJWH.S576568 | P < 5×10^-6 | r² < 0.001, window = 1000 kb | F-statistic | MR-Egger intercept; MR-PRESSO; PhenoScanner | Cochran Q; funnel plot | leave-one-out; replication analysis | 不存在 | FDR | 中 |
| Zhang | 2021 | Journal of Clinical Medicine | 10.3390/jcm10122688 | PCOS variants p < 5×10^-8; traits/outcomes IV selection thresholds not fully reported in the main text | LD R2 < 0.5 for selected PCOS-associated variants; clumping parameters for all MR instruments not fully reported | 不存在 | MR-Egger |  | weighted median | 不存在 | Bonferroni | 中 |
| Dang | 2025 | Diabetology & Metabolic Syndrome | 10.1186/s13098-025-01739-z | P < 5×10^-8 | r² < 0.001, window = 10000 kb | F-statistic | MR-Egger intercept; MR-PRESSO | Cochran Q; funnel plot | Steiger test; leave-one-out; BWMR | 不存在 | 未明确报告 | 中 |
| Li | 2023 | Heliyon | 10.1016/j.heliyon.2023.e22155 | P < 1.0 × 10^-5 | r² < 0.001；window = 不确定 | F-statistic | MR-Egger intercept | Cochran Q | leave-one-out; reverse MR analysis | 不存在 | FDR | 中 |
| Adams | 2020 | Scientific Reports | 10.1038/s41598-020-69114-8 | P < 5×10^-8 | r² < 0.001 | F-statistic | MR-Egger intercept; MR-Egger; weighted median; weighted mode; Radial MR | Cochran Q; scatter plot | SIMEX correction | For mediation of Education Years on T2D, multivariable MR adjusted for individual mediators; no PCOS-related mediation adjustment existed | Bonferroni | 中 |
| Lin | 2025 | PLOS ONE | 10.1371/journal.pone.0321380 | P < 5×10^-8 | r² < 0.01；window = 不确定 | F-statistic | MR-Egger intercept | Cochran Q; funnel plot | leave-one-out | 不存在 | Bonferroni | 中 |
| Aru | 2025 | Medicine | 10.1097/MD.0000000000042685 | Significant associations with LDL-C levels; pleiotropic SNP screening threshold P < 5 × 10^-8 in GWAS Catalog; exact SNP selection P threshold for instruments not fully reported | r² < 0.3, window = 100 kb around PCSK9 and HMGCR gene regions | F-statistic | MR-Egger intercept; MR-PRESSO; GWAS Catalog screening for pleiotropic SNPs | Cochran Q | leave-one-out; replication analysis | 不存在 | 不存在 | 中 |
| Kong | 2024 | BMC Women's Health | 10.1186/s12905-024-03347-x | P < 5×10^-8；当SNP少于3个时放宽至 P < 5×10^-6 | r² < 0.001, window = 10000 kb | F-statistic | MR-Egger intercept | Cochran Q | leave-one-out | 不存在 | 不存在 | 中 |
| Ma | 2023 | International Journal of Gynecology and Obstetrics | 10.1002/ijgo.14820 | P < 5×10^-8 | r² ≤ 0.01 | F-statistic | MR-Egger intercept; MR-PRESSO | Cochran Q; funnel plot | leave-one-out | 不存在 | 不存在 | 中 |
| Ruth | 2020 | Nat Med | 10.1038/s41591-020-0751-5 | P < 5×10^-8 | 1Mb distance-based clumping; long-range LD variants with r2>0.05 excluded; proxies with r2>0.5 within 250 kb for missing outcome variants | 不存在 | MR-Egger intercept; MR-Egger |  | Steiger test; replication analysis; weighted median | 不存在 | 未明确报告 | 中 |
| Gao | 2024 | Journal of Ovarian Research | 10.1186/s13048-024-01420-5 | P < 5×10^-8 | r² < 0.001, window = 10000 kb | F-statistic/R² | MR-Egger intercept; MR-PRESSO | Cochran Q; funnel plot | leave-one-out | MVMR adjusted for BMI; BMI, FBG, and FI; mediation/MVMR included bioavailable testosterone | 不存在 | 中 |
| Sun | 2020 | Frontiers in Endocrinology | 10.3389/fendo.2020.00621 | P < 1 × 10^-5 | r² < 0.1, window = 500 kb | F-statistic/R² | MR-Egger; MR-PRESSO; MR-PRESSO global test |  | weighted median | 不存在 | 不存在 | 中 |
| Ye | 2025 | International Journal of Women's Health | 不确定 | P < 1×10^-5（因严格阈值 P < 5×10^-8 下 SNP 数量不足） | r² = 0.001, window = 10000 kb | F-statistic | MR-PRESSO | Cochran Q; funnel plot | leave-one-out | 不存在 | 不存在 | 中 |
| Guo | 2024 | BMC Women's Health | 10.1186/s12905-024-03144-6 | P < 5×10^-8 | r² < 0.001, window = 10000 kb | F-statistic | MR-Egger intercept; MR-PRESSO | Cochran Q | replication analysis | 使用BMI-adjusted SHBG SNPs作为替代IV集；不存在明确MVMR调整模型 | Bonferroni | 中 |
| Fang | 2025 | International Journal of Women's Health | 不确定 | P < 5×10^-8 | r² < 0.01; using 1000 Genomes European reference panel; window = 不确定 | F-statistic | MR-Egger intercept; MR-PRESSO | Cochran Q | Steiger test; leave-one-out | 中介变量为BMI | 不存在 | 中 |
| Deshmukh | 2022 | Bone | 10.1016/j.bone.2021.116285 | 不确定 | 不确定 | 不存在 | MR-Egger intercept |  |  | Adjusted for age, BMI, and top five principal components in regression models; 不存在中介或MVMR | 不存在 | 低 |
| Chen | 2025 | Journal of Multidisciplinary Healthcare | 不确定 | P < 1×10^-5 | r² < 0.1, window = 500 kb | F-statistic | MR-Egger intercept; MR-PRESSO | Cochran Q; funnel plot | Steiger test; leave-one-out | 不存在 | 不存在 | 中 |
| Shen | 2023 | World Journal of Clinical Cases | 10.12998/wjcc.v11.i23.5468 | Vitamins D, E, A, and B12: P < 5×10^-8; vitamin K: P < 5×10^-6 | r² < 0.01, window = 1000 kb | 不存在 | MR-Egger intercept; MR-PRESSO | Cochran Q | weighted median; MR-RAPS; MR-PRESSO | 不存在 | 不存在 | 中 |
| Xue | 2025 | Clinical and Experimental Reproductive Medicine | 10.5653/cerm.2025.08543 | 不确定 | 不确定 | 不确定 | 不存在 | 不存在 | leave-one-out; scatter plot; 不确定 | 不存在 | 不存在 | 低 |
| Hu | 2025 | International Journal of Diabetes in Developing Countries | 10.1007/s13410-024-01360-2 | PCOS as exposure: P < 1×10^-5; sensitivity analysis used P < 1×10^-6 | LD independent SNPs selected; proxy SNPs with r² > 0.8 when target SNPs unavailable; clumping阈值其余参数不确定 | 不存在 | MR-Egger intercept |  | Steiger test; weighted median | 不存在 | 不存在 | 低 |
| Brower | 2019 | Human Reproduction | 10.1093/humrep/dey343 | BMI instrument used SNPs robustly associated with BMI; PCOS instrument used SNPs associated with PCOS at genome-wide significance | 不确定 | F-statistic | MR-Egger intercept | funnel plot | MR-Egger; maximum likelihood | 不存在 | Bonferroni | 中 |
| Tan | 2024 | Frontiers in Endocrinology | 10.3389/fendo.2024.1359236 | P ≤ 5×10^-8；若存在异质性则采用 1×10^-6 | r² < 0.001, window = 1000 kb | F-statistic | MR-Egger; MR-PRESSO | Cochran Q | weighted median; simple mode; weighted mode | 不存在 | 不存在 | 中 |
| Verdiesen | 不确定 | 不确定 | 不确定 | lead SNPs identified from AMH GWAS; genome-wide significant loci reported as p < 5×10^-8 | 不确定 | F-statistic | comparison of IVW with SNP-specific Wald ratio estimates | Cochran Q | leave-one-out | 不存在 | 不存在 | 中 |
| Xing | 2025 | Gynecology and Obstetrics Clinical Medicine | 10.1136/gocm-2025-000268 | P < 5×10^-8; to maximise explained variance, a threshold of 1×10^-5 was also described | r² < 0.001, window = 10000 kb | F-statistic | MR-Egger intercept; MR-PRESSO | Cochran Q; scatter plot | leave-one-out | 不存在 | FDR | 中 |
| Lim | 2021 | Genes & Genomics | 10.1007/s13258-021-01134-8 | Genetic variants showing suggestive association with blood insulin level (P < 10^-5) were selected; MR finally used 7 SNPs after exclusions | 不确定 | 不存在 |  |  |  | 不存在 | 不存在 | 低 |
| Verdiesen | 2022 | Human Reproduction | 10.1093/humrep/deac028 | P < 5×10^-8 | 不存在 | F-statistic | 比较IVW与单SNP Wald ratio估计 | Cochran Q | leave-one-out | 不存在 | 不存在 | 中 |
| Guo | 2026 | International Journal of Women's Health | 10.2147/IJWH.S583951 | P < 5×10^-8 | r² < 0.001, window = 10000 kb | F-statistic | MR-Egger intercept | Cochran Q; funnel plot | Steiger test; leave-one-out | 不存在 | 未明确报告 | 中 |
| Dilimulati | 2025 | Nature and Science of Sleep | 不确定 | P < 5×10^-8 | r² < 0.001, window = 10000 kb | F-statistic | MR-Egger intercept; MR-PRESSO | Cochran Q; funnel plot | leave-one-out | BMI | 不存在 | 中 |
| She | 2025 | Reproductive and Developmental Medicine | 10.1097/RD9.0000000000000108 | 主要为 P < 5×10^-8；反向 MR 中部分女性生殖系统疾病因 SNP 较少改为 P < 5×10^-6 | r² < 0.001, window = 1000 kb | F-statistic | MR-Egger intercept; MR-PRESSO | Cochran Q; funnel plot | leave-one-out | 不存在 | 未明确报告 | 中 |
| Yan | 2026 | International Journal of Women’s Health | 10.2147/IJWH.S605528 | P < 5×10^-6 | r² < 0.001, window = 10000 kb | F-statistic/R² | MR-Egger intercept; funnel plot | Cochran Q; funnel plot | leave-one-out; bidirectional MR; weighted median; weighted mode; simple mode | 不存在 | 未明确报告 | 中 |
| Yang | 2026 | Brain Imaging and Behavior | 10.1007/s11682-026-01143-9 | P < 5×10^-6 | r² < 0.001, window = 10 Mb | F-statistic | MR-Egger intercept; MR-PRESSO global test | Cochran Q; funnel plot | leave-one-out | 不存在 | Bonferroni | 中 |
| He | 2026 | Journal of Ovarian Research | 10.1186/s13048-026-02014-z | P < 5.0 × 10^-8 | SMR-multi used SNPs within default 500 kb probe window with LD r² < 0.9 relative to the top-associated SNP; main SMR cis-window ±1000 kb | 不存在 | HEIDI | HEIDI | colocalization analysis; replication analysis | 不存在 | 未明确报告 | 中 |
| Martin | 不确定 | 不确定 | 不确定 | Variants associated at P < 5×10^-8 with both body fat percentage and a composite metabolic phenotype | 不确定 | 不存在 | MR-Egger | I² | weighted median | 不存在 | 不存在 | 中 |
| Cao | 2024 | J. Bio-X Res. | 10.34133/jbioxresearch.0024 | Europeans: P < 8.31×10^-9; East Asians: P < 5×10^-8 | r² > 0.01 used to remove variants in linkage disequilibrium | F-statistic | MR-Egger intercept; funnel plot | Cochran Q; funnel plot | leave-one-out; replication analysis | 不存在 | 不存在 | 中 |
| Zhang | 2024 | Human Reproduction | 10.1093/humrep/deae244 | P < 5×10^-8 | r² < 0.001, window = 不确定 | F-statistic | MR-Egger intercept; MR-PRESSO | Cochran Q; I² | leave-one-out | 不存在 | FDR | 中 |
| Moolhuijsen | 2026 | Nature Genetics | 10.1038/s41588-026-02543-9 | not explicitly reported for MR instruments in provided text | 不确定 | 不存在 | MR-Egger intercept | I2 |  | BMI adjusted using multivariate inverse-weighted method; beta coefficients for BMI taken from GIANT consortium | 未明确报告 | 中 |
| Li | 2026 | Journal of Ovarian Research | 10.1186/s13048-026-01970-w | P < 5×10^-8 | r² < 0.001, window = 10000 kb | F-statistic | MR-Egger intercept; MR-PRESSO | Cochran Q | weighted median; maximum likelihood; MR-Lap | 不存在 | FDR | 中 |
| Martin | 2022 | eLife | 10.7554/eLife.72452 | P < 5×10^-8 | 不确定 | 不存在 | MR-Egger | Cochran Q; I2 | weighted median; replication analysis | 不存在 | FDR | 中 |
| Xin | 2025 | International Journal of Women's Health | 不确定 | P < 5×10^-8; 对 OD 和 PCOS 的 two-step MR 因显著位点有限，额外使用 p < 5e-6 | r² < 0.001, window = 10000 kb | F-statistic/R² | MR-Egger intercept; MR-PRESSO | Cochran Q; funnel plot | leave-one-out | MVMR adjusted for BMI, depression, ischemic heart disease, diabetes; additional lifestyle adjustments for spontaneous abortion included smoking, alcohol consumption, coffee intake, insomnia; mediation variable was BMI | 不存在 | 中 |
| Leinonen | 2023 | Communications Medicine | 10.1038/s43856-022-00226-0 | P < 5×10^-8 | 独立lead SNP定义为每染色体递归选择最低P值SNP，并移除其两侧500 kb区域；传统MR clumping参数未明确完整报告 | 不存在 | MR-Egger; CAUSE |  | replication analysis; multivariable MR; cross-sex analyses | multivariable MR-Egger adjusted for SHBG; for some female reproductive phenotypes adjusting for PCOS had negligible effects | Bonferroni | 中 |
| Day | 2018 | PLoS Genetics | 10.1371/journal.pgen.1007813 | 不确定 | 不确定 | 不存在 | MR-Egger intercept | 不存在 | 不存在 | 不存在 | Bonferroni | 中 |
| Ruth | 2020 | Nat Med | 10.1038/s41591-020-0751-5 | P < 5×10^-8 | 1Mb distance-based clumping; long-range LD correlated variants excluded at r2>0.05; proxy search within 250kb with r2>0.5 for missing outcome variants | 不存在 | MR-Egger intercept |  | Steiger test; replication analysis; weighted median; MR-Egger | 不存在 | 未明确报告 | 中 |
| Fang | 2025 | International Journal of Women's Health | 不确定 | P < 5×10^-8 | r² < 0.01；window = 不确定 | F-statistic | MR-Egger intercept; MR-PRESSO | Cochran Q | Steiger test; leave-one-out | 中介变量：BMI | 不存在 | 中 |
| Harris | 2019 | International Journal of Epidemiology | 10.1093/ije/dyz113 | P < 5×10^-8 | Reported that none was in linkage disequilibrium; specific r²/window not reported | 不存在 | MR-Egger intercept | Cochran Q | weighted median | 不存在 | 不存在 | 中 |
| Min | 2024 | Frontiers in Endocrinology | 10.3389/fendo.2023.1282056 | P < 5×10^-6；但结果部分对AMH和Estradiol写为P < 5×10^-8 | r² < 0.001, window = 10000 kb | F-statistic | MR-Egger intercept; MR-PRESSO | Cochran Q | leave-one-out | 不存在 | 不存在 | 中 |
| Guan | 2024 | Frontiers in Endocrinology | 10.3389/fendo.2024.1337562 | P < 5×10^-8 | r² < 0.001, window = 10000 kb | F-statistic | MR-Egger intercept | Cochran Q; funnel plot | leave-one-out | 不存在 | 不存在 | 中 |
| Pu | 2023 | BMC Medical Genomics | 10.1186/s12920-023-01581-0 | PCOS作为暴露时: P < 5×10^-8；reverse MR中OS指标作为暴露时: P < 1×10^-5 | PCOS作为暴露时: r² < 0.001, window > 10000 kb；reverse MR中OS指标作为暴露时: r² < 0.01, distance > 250 kb | F-statistic | MR-Egger intercept | Cochran Q; funnel plot | leave-one-out | 不存在 | 不存在 | 中 |
| Day | 2015 | Nature Communications | 10.1038/ncomms9464 | based on reported GWAS signals for 15 traits; genome-wide significance threshold for instrument selection not explicitly stated in MR methods | 不确定 | 不存在 | 不确定（未描述方法） | funnel plot | funnel plot | 不存在 | Bonferroni | 中 |
| Motafeghi | 2025 | Int J Endocrinol Metab | 10.5812/ijem-159124 | P < 5×10^-8 | r² < 0.001, window = 10000 kb | F-statistic | MR-Egger intercept; MR-PRESSO; PhenoScanner | Cochran Q; I²; funnel plot; Rucker’s Q | leave-one-out | 不存在 | 不存在 | 中 |
| Bi | 2024 | Breast Cancer Research | 10.1186/s13058-024-01923-5 | 不确定 | 不确定 | 不存在 |  |  |  | 不存在 | Bonferroni | 低 |
| Verdiesen | 不确定 | 不确定 | 不确定 | Lead SNPs identified at p < 5×10^-8 from the AMH GWAS meta-analysis were used as instruments | 不确定 | F-statistic | Comparison of overall IVW estimate with SNP-specific Wald ratio estimates | Cochran Q | leave-one-out | 不存在 | 不存在 | 中 |
| Verdiesen | 2022 | Human Reproduction | 10.1093/humrep/deac028 | Lead SNPs reaching genome-wide significance (P < 5×10^-8) in the AMH GWAS meta-analysis | 不确定 | F-statistic | comparison of overall IVW estimate to SNP-specific Wald ratio estimates | Cochran Q | leave-one-out | 不存在 | 不存在 | 中 |
| Martin | 2022 | eLife | https://doi.org/10.7554/eLife.72452 | P < 5×10^-8 | 不确定 | 不存在 | MR-Egger | Cochran Q; I² | weighted median; UK Biobank validation | 不存在 | FDR | 中 |
| Zhang | 2024 | Combinatorial Chemistry & High Throughput Screening | 10.2174/0113862073325251241101054306 | P < 5×10^-8 | r² ≤ 0.001, window = 10000 kb | F-statistic/R² | MR-Egger intercept; funnel plot | Cochran Q; funnel plot | leave-one-out | 不存在 | 不存在 | 中 |
| Yarmolinsky | 2019 | PLoS Medicine | 10.1371/journal.pmed.1002893 | P < 5×10^-8 | r² < 0.001, window = 10000 kb | F-statistic/R² | MR-Egger intercept; weighted median; weighted mode |  | Steiger test; leave-one-out | 不存在 | Bonferroni | 中 |
| Xiao | 2023 | Journal of the American Heart Association | 10.1161/JAHA.121.026561 | 2-sample MR exposure SNPs: P < 5×10^-8 | r² = 0.1, window = 250 kb | F-statistic | MR-Egger intercept; MR-Egger |  | weighted median; bidirectional MR | 不存在 | FDR | 中 |
| Kho | 2021 | Human Genetics | 10.1007/s00439-021-02312-0 | P < 5×10^-8 | r² < 0.01 | 不存在 | MR-Egger intercept; GSMR HEIDI outlier test | Cochran Q | leave-one-out; weighted median; weighted mode; GSMR | 不存在 | Bonferroni | 中 |
| Naz | 2025 | medRxiv | 10.1101/2025.11.07.25339752 | P < 5 × 10^-8 | r² < 0.01, window = 10000 kb | 不存在 | 不存在 | 不存在 | 不存在 | 不存在 | Bonferroni | 低 |
| Wei | 不确定 | 不确定 | 不确定 | P < 5×10^-6 | r² < 0.001 | F-statistic | MR-Egger intercept; MR-PRESSO | Cochran Q; funnel plot | leave-one-out | 不存在 | 不存在 | 中 |
| Petersen | 2024 | 不确定 | 不确定 | P < 5×10^-8 | r² < 0.01, window = 1000 kb | 不存在 | CAUSE; MR-Egger |  | mode-based estimate; IVW; MR-Egger | 不存在 | FDR | 中 |
| De Silva | 不确定 | 不确定 | 不确定 | P < 5×10^-8 | r² < 0.001, window = 10000 kb | F-statistic | MR-Egger intercept; MR-PRESSO | Cochran Q; funnel plot | leave-one-out; Radial MR | 不存在 | Bonferroni | 高 |
| Xiao | 不确定 | 不确定 | 不存在 | genome-wide significant SNPs | LD pruned according to LD patterns in the 1000 Genomes HapMap SNPs | 不存在 | MR-Egger |  | weighted median | 不存在 | FDR | 中 |
| Yang | 2026 | Brain Imaging and Behavior | 10.1007/s11682-026-01143-9 | P < 5×10^-6 | r² < 0.001, window = 10 Mb | F-statistic | MR-Egger intercept; MR-PRESSO global test | Cochran Q; funnel plot | leave-one-out; weighted median; weighted mode; MR-Egger | 不存在 | Bonferroni | 中 |
| Hu | 2025 | International Journal of Diabetes in Developing Countries | 10.1007/s13410-024-01360-2 | PCOS as exposure: P < 1E−5 for main MR; sensitivity analysis used P < 1E−6 | 仅报告 LD independent SNPs；proxy SNPs with r2 > 0.8 when target SNPs unavailable；其余不确定 | 不存在 | MR-Egger intercept |  | Steiger test; weighted median | 不存在 | 不存在 | 中 |
| Hu | 2025 | International Journal of Diabetes in Developing Countries | 10.1007/s13410-024-01360-2 | PCOS as exposure: P < 1E−5; sensitivity analysis used P < 1E−6 | 不确定 | 不存在 | MR-Egger intercept | 不存在 | Steiger test; weighted median | 不存在 | 不存在 | 低 |
|  |  |  |  |  |  |  |  |  |  |  |  |  |
